# Harmine derivative B-9-3 inhibits non-small cell lung cancer via the VEGFA/PI3K/AKT pathway

**DOI:** 10.3389/fphar.2025.1526952

**Published:** 2025-05-13

**Authors:** Yuche Wu, Bing Wang, Xuwen Mao, Wei Chen, Haji Akber Aisa

**Affiliations:** 1 Xinjiang Technical Institute of Physics and Chemistry Chinese Academy of Sciences, Urumqi, Xinjiang, China; 2 Xinjiang Huashidan Pharmaceutical Co., Ltd., Urumqi, Xinjiang, China; 3 The Fourth Affiliated Hospital of Xinjiang Medical University, Urumqi, Xinjiang, China; 4 College of Pharmacy, Xinjiang Medical University, Urumqi, Xinjiang, China

**Keywords:** harmine derivative B-9-3, non-small cell lung cancer, apoptosis, angiogenesis, VEGFA/PI3K/AKT

## Abstract

**Background:**

This study aimed to investigate the molecular mechanism by which the Harmine derivative B-9-3 inhibits angiogenesis and promotes apoptosis in non-small cell lung cancer (NSCLC).

**Methods:**

Three non-small cell lung cancer (NSCLC) models (human NSCLC cell line A549, human lung squamous cell carcinoma cell line H226, human large cell lung carcinoma cell line H460) were established. Cell proliferation was assessed using CCK-8 assays and colony formation assays. Cell motility was evaluated through scratch wound healing, invasion, and migration assays. Cell apoptosis was analyzed by Hoechst 33258 staining, AO/EB fluorescence staining, and flow cytometry. Real-time PCR was used to measure the mRNA expression of B-cell lymphoma/leukemia-2 (Bcl-2), Bcl-2-associated X protein (Bax), and Caspase-3, while Western blotting was performed to assess the protein levels of vascular endothelial growth factor A (VEGFA), phosphatidylinositol 3-kinases p110 Beta (PI3K), phospho-phosphatidylinositol 3-kinases (p-PI3K), protein kinase B (AKT), phosphorylated protein kinase B (p-AKT), Bax, Bcl-2, and Caspase-3.

**Results:**

Compared to the control group, B-9-3 (50, 100, 200 μg/mL) inhibited the growth and motility of the three types of lung cancer cells, suppressed cell invasion and migration, and promoted cell apoptosis and necrosis. The apoptosis rates in three types of non-small cell lung cancer (NSCLC) cells were significantly increased. The mRNA expressions of Bax and Caspase-3 were markedly upregulated, while that of Bcl-2 was significantly downregulated. Additionally, the protein levels of VEGFA, p-PI3K/PI3K, p-AKT/AKT, and Bcl-2 were notably reduced, whereas the protein levels of Bax and Caspase-3 were significantly elevated.

**Conclusion:**

The harmine derivative B-9-3 may exert its anti-NSCLC effects by inhibiting angiogenesis and promoting lung cancer cell apoptosis via the VEGFA/PI3K/AKT signaling pathway.

## Graphical Abstract

**Figure:**
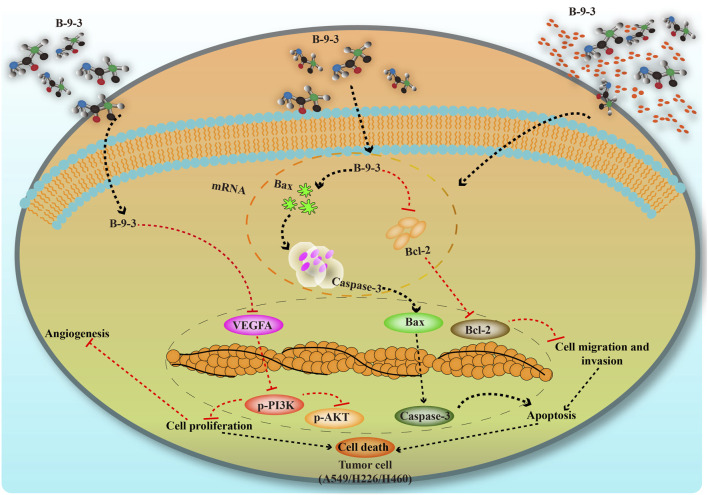

## Highlights

- Harmine derivative B-9-3 inhibits angiogenesis and promotes apoptosis in non-small cell lung cancer (NSCLC).
- Harmine derivative B-9-3 by regulate the expression of apoptosis gene and protein family play a role of promoting apoptosis.
- Harmine derivative B-9-3 by regulate the expression of vascular growth factor related protein play a role of anti-angiogenesis.
- Harmine derivative B-9-3 adjustable VEGFA//PI3K/AKT signaling pathway.

## 1 Introduction

Lung cancer is one of the malignant tumors with leading incidence and mortality rates globally. Non-small cell lung cancer (NSCLC) is the major pathological type of lung cancer, accounting for approximately 80% (Xia et al., 2022). NSCLC primarily encompasses three subtypes: lung adenocarcinoma (LUAD) (constituting 50%–60%), lung squamous cell carcinoma (LUSC) (occupying 25%–30%), and large cell lung cancer (accounting for 5%–10%) (Guo et al., 2022; Bray et al., 2018). Despite advances in the medical treatment of NSCLC, its clinical survival rate remains low, making the search for effective therapeutic drugs a current research focus (GUO et al., 2019).

In recent years, increasing research has revealed that tumor angiogenesis is a critical process in NSCLC progression, with vascular endothelial growth factor (VEGF) being a key mediator in this process (Lee et al., 2016; Jayakumar et al., 2018; Wu and Zhang, 2016). VEGFA, a potent angiogenic factor within the VEGF family, stimulates endothelial cell proliferation and accelerates new blood vessel formation, thereby providing a foundation for tumor cell growth and proliferation (Seyedmirzaei et al., 2020). Widely expressed in damaged vascular endothelial cells, VEGFA increases endothelial progenitor cell migration and significantly promotes angiogenesis in lung cancer (Yz et al., 2022). When the number of lung cancer cells reaches a certain threshold, they secrete various VEGF factors to form a new vascular network around their tissue, promoting intratumoral vascular circulation (Gu et al., 2021). Therefore, inhibiting angiogenesis in lung cancer has become an important therapeutic strategy.

Harmine is a naturally sourced β-carboline alkaloid with broad pharmacological activities, and its antitumor activity has attracted extensive attention in recent years (Nan, 2022). Harmine inhibits tumor cell angiogenesis and exerts anticancer effects (Tang, 2020). Studies have shown that harmine can reduce tumor-directed capillary formation in melanoma tumors in mice (Zhang et al., 2023). Ruan (2018) proposed a mechanism whereby harmine inhibits angiogenesis and tumor growth by activating the p53 protein pathway. However, harmine can also have neurotoxic effects through inhibition of amine neurotransmitters or direct interactions with specific receptors in the central nervous system (Yu et al., 2022). Wei et al. (2022) synthesized the harmine derivatives 2DG-Har-0 and MET-Har-02 by modifications at positions 2, 7, and 9, finding that these derivatives exhibited better anticancer effects than unmodified harmine. In our previous studies, we found that modifications to the substituent at position nine of harmine resulted in reduced toxicity and enhanced efficacy. Based on these findings, we synthesized a novel anti-tumor compound, harmine derivative B-9-3. Subsequent investigations revealed significant alterations in its anti-tumor activity and neurocytotoxicity (Li et al., 2015; Piekarski et al., 2014; Phalen et al., 2013).

We have previously reported that B-9-3 regulated the recruitment of immune cells in the tumor microenvironment and inhibited tumor angiogenesis by blocking the transforming growth factor receptor 1 (TGFβRI) and vascular endothelial growth factor receptor 2 (VEGFR2) signaling pathways, demonstrating significant anticancer effects *in vitro* and *in vivo* (Meixin and Youran, 2024; Zhang, 2020). However, the downstream mechanism by which B-9-3 regulates VEGF signaling remains unclear and requires further exploration. Therefore, this study selected three NSCLC cell lines, namely, the A549 (LUAD), NCL-H226 (LUSC), and NCL-H460 (large cell lung carcinoma) cell lines, to evaluate the anti-NSCLC effects of harmine derivative B-9-3 and investigate its roles in blocking angiogenesis and proliferation while promoting apoptosis in tumor cells, and thereby elucidate the molecular mechanism by which B-9-3 regulates the VEGFA/PI3K/AKT pathway in NSCLC.

## 2 Methods

### 2.1 Chemical reagents and experimental equipment

RPMI-1640 medium, fetal bovine serum, phosphate buffer solution, 0.25% trypsin, and penicillin-streptomycin (Gibco, Catalog No: 12633012, 10091-148, AM-9624, 25200056, 10378016); Cell-Counting-Kit-8--MedChemExpress (CCK8), Efficient RIPA tissue/cells fast pyrolysis liquid, protease inhibitors, 10% SDS-PAGE gel preparation kit, Hoechst 33258, 5 * protein sample buffer, 20*TBST, DNase/RNase-Free Water, Trizol Substitute, Protein Phosphatase Inhibitor (All-in-one,100x) (Solarbio, Catalog No: CA1210, R0010, P6730, P1203, IH0060, P1040, T1082, R1600, R1100, P1260); Acridine Orange(AO)/EB Double Stain Kit (Sangon, Catalog No:E607308); TaqMan™MicroRNA Reverse Transcription Kit, Micro BCA™Protein Assay Kit, Prestained Protein Ladder(Thermo Scientific™, Catalog No: 4366596, 23235, 26616); glycine, Tris-HCL(Biotopped, Catalog No: G6200A, T6230T); SDS(BIOFROX, Catalog No: 3250); Difco TM Skim Milk(BD, Catalog No: 232100); GAPDH Polyclonal antibody, VEGFA Polyclonal antibody, Phospho-AKT antibody, Caspase-3 Polyclonal antibody, Bcl-2 Polyclonal antibody, Bax Polyclonal antibody, PI3K p110 Polyclonal antibody, AKT Polyclonal antibody, HRP-conjugated Goat Anti-Rabbit IgG(H + L) (Proteintech, Catalog No: 10494-1-AP,19003-1-AP,19677-1-AP,66444-1-Ig,26593-1-AP,50599-2-Ig,20584-1-AP,10176-2-AP,SA00001-2); Phospho PI3K p110 antibody (Cell Signaling Technology, Catalog No: 4255S); ECL (Biosharp, Catalog No: BL523A); Annexin V PE Apoptosis kit (BD, Catalog No: 559763).

CO_2_ incubator, Flow cytometer (Thermo Fisher, No: Midi 40, Attune CytPix); Electronic balance (Sartorius, No: SQP); Benchtop refrigerated centrifuge (Eppendorf, No: 5424R); Vortex mixer (Thermo, No: IKA-VORTEX3); Full wavelength microplate reader, Vertical electrophoresis system, Gel imager, Real-time PCR instrument (Bio-Rad, No: Benchmark PLUS, 165-8033, GS-900, PCR-5115); Inverted fluorescence microscope (OLYMPUS, No: IX73).

### 2.2 Experimental drugs

The harmine derivative B-9-3 was provided by Xinjiang Huashidan Pharmaceutical Co., Ltd. The appearance of the sample is a yellow powder. It was diluted to 2 mg/mL with sterile saline for *in vitro* cell experiments.

### 2.3 Cell culture

Three human lung cancer cell lines, A549 (adenocarcinoma), NCL-H226 (squamous cell carcinoma), and NCL-H460 (large cell carcinoma), were purchased from the National Collection of Authenticated Cell Cultures (Shanghai). The cells were cultured in RPMI-1640 medium supplemented with 10% fetal bovine serum (FBS), 100 U/mL penicillin, and 100 U/mL streptomycin at 37°C in a 5% CO_2_ incubator. Passaging was performed every 2–3 days using 0.25% (w/v) trypsin.

### 2.4 Cell proliferation

Log-phase A549, H226, and H460 cells were seeded at 1 × 10^4^ cells/mL in 96-well plates with 100 μL per well. After 24 h, the original culture medium was replaced. The cells were divided into 6 groups, namely, the control group (cultured with complete medium, with medium change every 24 h) and the B-9-3 treatment groups (50, 100, 150, 200, 250 μg/mL). After 72 h of pre-treatment with B-9-3 solutions prepared in complete medium, 10 μL of CCK-8 reagent was added to each well. Plates were incubated at 37°C in a 5% CO_2_ incubator for 2 h, and optical density (OD) was measured at 450 nm using a microplate reader.

### 2.5 Observations of cell morphology

Log-phase A549, H226, and H460 cells were adjusted to 1 × 10^5^ cells/mL, seeded at 2 mL per well in 6-well plates, and cultured at 37°C in a 5% CO_2_ incubator. After 24 h, cells were treated according to the results of Section 2.4 (50, 100, 200 μg/mL) and observed and imaged under an inverted microscope.

### 2.6 Colony formation assay

Log-phase A549, H226, and H460 cells were adjusted to 2 × 10^3^ cells/mL, seeded at 2 mL per well in 6-well plates, and cultured at 37°C in a 5% CO_2_ incubator. After 7 days of culture and treatment as per Section 2.4 (50, 100, 200 μg/mL), cells were further cultured for 14 days, fixed with methanol, and stained with crystal violet. Colonies in each well were counted under an inverted microscope.

Colony forming efficiency = Number of colonies/Number of seeded cells × 100%.

### 2.7 Cell scratch assay

Log-phase A549, H226, and H460 cells were adjusted to 2 × 10^5^ cells/mL, seeded at 2 mL per well in 6-well plates, and cultured at 37°C in a 5% CO_2_ incubator. When the cells reached near 90% confluence, the medium was replaced with serum-free RPMI-1640 for 12 h to induce starvation. Scratches were gently made using a 10 μL pipette tip, and cell debris was washed off with phosphate-buffered saline (PBS). The initial scratch was photographed as the 0-h time-point image. After treatment according to Section 2.4 (50, 100, 200 μg/mL), cells were incubated for 24 and 72 h, and images were taken under a microscope. The width of scratches was analyzed using ImageJ software.

Cell migration rate = Migration distance in treatment group/Migration distance in control group × 100%.

### 2.8 Cell invasion and migration assays

Transwell chambers (Millipore, Burlington, MA, United States) were used to assess invasion and migration in the three types of lung cancer cells. Following the procedures outlined in Section 2.4, A549, H226, and H460 cells were grouped and treated accordingly. After culturing, cells from each group were resuspended in serum-free RPMI-1640 medium and adjusted to a cell density of 2 × 10^5^ cells/mL. One 24-well Transwell chamber plate was used for invasion experiments, while another plate was used for migration experiments. The upper chamber was precoated with Matrigel (Corning, NY, United States) before cell seeding. One hundred microliters of cell suspension were placed in the upper chamber of both plates while medium containing 10% FBS was added to the lower chamber. The chambers were incubated at 37°C with 5% CO_2_ for 48 h. After incubation, cells on the upper side of the membrane that failed to cross the membrane were wiped off with a cotton swab, fixed with 4% paraformaldehyde for 10 min, stained with 10% crystal violet for 15 min, and counted under an optical microscope. The number of cells that penetrated the membrane was counted in six random fields, with the average number used to calculate the invasion and migration capabilities of the cells.

Percentage of invasive cells (%) = (number of cells penetrating the membrane in treatment group/number of cells penetrating the membrane in control group) * 100%.

Percentage of migratory cells (%) = (number of cells penetrating the membrane in treatment group/number of cells penetrating the membrane in control group) * 100%.

### 2.9 Hoechst 33258 staining

A549, H226, and H460 cells were grouped and treated following the procedures outlined in Section 2.4. After discarding the supernatant, the A549, H226, and H460 cells were washed twice with pre-chilled PBS for 5 min each time. The cells in each well were fixed with 1 mL of 4% paraformaldehyde for 1 h at 4°C, washed twice with PBS for 10 min each time, and stained with 1 mL of Hoechst 33258 fluorescent dye in the dark for 15 min. After removing the staining solution, the cells were washed twice with PBS for 10 min each time in the dark. Fluorescence microscopy (OLYMPLUS IX73, Japan) was used for observation and imaging. The ultraviolet excitation wavelength of the fluorescence microscope was 350 nm, and the emission wavelength was 460 nm.

### 2.10 AO/EB staining method for detecting apoptosis

A549, H226, and H460 cells were seeded in 6-well plates at a density of 1 × 10^6^ cells per well. Following the grouping and treatment procedures outlined in Section 2.4, the cells were washed twice with pre-cooled PBS for 10 min each time. Subsequently, 10 μL of a 1:1 mixture of AO and EB staining solution was added to each well, and the cells were incubated in the dark at room temperature for 5 min. Morphological features of apoptosis and necrosis were then examined under a fluorescence microscope, and images were captured. The number of apoptotic and necrotic cells in each group was quantified by counting cells from 10 randomly selected fields across 5 distinct regions. Changes in cellular morphology were documented through photographic records.

Apoptosis rate (%) = (Apoptotic cell numbers)/(Apoptotic cell numbers + Normal cell numbers).

### 2.11 Flow cytometry for detecting cell apoptosis

A549, H226, and H460 cells were seeded in 6-well plates at a density of 1 × 10^5^ cells per well and cultured until the cell density reached approximately 80%. The medium was then replaced with RPMI-1640 medium containing different concentrations (50, 100, 200 μg/mL) of B-9-3, and cells were further incubated in a CO_2_ incubator for 72 h. The cells were then collected, digested, centrifuged, washed, and placed in flow cytometry tubes. Next, the cells were suspended in 400 μL 1×Annexin V binding solution, together with 5 μL of Annexin V dye, shaken and mixed, and incubated in the dark at 4°C for 15 min. Then, 10 μL of propidium iodide (PI, Annexin V-FITC/PI Apoptosis detection kit, Abcam, AB-r4532) was added and incubated in the dark at 4°C for 5 min. Apoptosis was detected using flow cytometry (ThermoFisher, Attune CytPix).

### 2.12 Determination of Bax, Bcl-2, Caspase-3 mRNA levels

Total RNA was extracted using TRIzol, according to the provided instructions, and the concentration and purity of total RNA were determined spectrophotometrically at A260/280 nm, the ratio of which was in the range of 1.8-2.0. RNA was then reverse transcribed to cDNA using the RevertAid First-Strand cDNA Synthesis Kit. According to the manufacturer about SYBR ^®^ premixed Ex TaqII rt-pcr kit instructions using 1 μg cDNA preparation qRT-PCR reaction system. Specific primers were used to amplify Bax, Bcl-2, Caspase-3, and the reference gene GAPDH in A549, H226, and H460 cells. The primer sequences are listed in Table 1. The 25 μL fluorescence quantitative PCR reaction system included SYBR Premix (12.5 μL), ddH_2_O (8.5 μL), and 1.0 μL of each upstream and downstream primer. The reaction was performed on an IQ5 fluorescence quantitative PCR instrument (Bio-Rad, Hercules, CA, United States) with the following program: initial denaturation at 95°C for 35 s, followed by 40 cycles of denaturation at 95°C for 8 s, annealing at 58°C for 40 s, and extension at 72°C for 35 s. Finally, each sample was analyzed in triplicate by the Real-Time PCR System (Bio-Rad PCR5115). β-actin was used as a housekeeping gene for normalization. Relative gene expression was analyzed using the 2^−ΔΔCT^ method (ΔΔCt = ΔCt [treated]-ΔCt[control]).

**TABLE 1:** Primers used for qRT-PCR analysis.

Gene	Primer sequences	TM	Length/bp
β-actin	F:CGT TGA CAT CCG TAA AGA CC	52.5	20
	R:AAC AGT CCG CCT AGA AGC AC		21
Bax	F:CACTCGTTCAGGTCAACACAAA	58.5	22
	R:TCCCTGTTCGAGCAGCACACTG		23
Bcl-2	F:GTACAAACGCCCAGGAGACCAG	62.9	25
	R:GACGAGTAACACTGCGAGACCT		26
Caspase-3	F:CACCTCTCTGAACAACGGAGGG	61.2	22
	R:CTTTGGCCTGGACGAAGAAGAT		22

### 2.13 Western blotting analysis of VEGFA, PI3K (p110 Beta), AKT, p-PI3K, p-AKT, Bax, Bcl-2, Caspase-3 protein expression

A549, H226, and H460 cells in the logarithmic growth phase were seeded in 100 mm culture dishes and cultured for 24 h. Following the procedures outlined in Section 2.4, cells were grouped and treated. After rinsing three times with pre-chilled PBS. Add 200 μL of RIPA lysis buffer and gently pipette up and down until the solution becomes homogeneous. Vortex to ensure thorough mixing, then incubate on ice for 60 min. Following this, centrifuge at 4°C at 14,000 rpm for 20 min and collect the supernatant. The protein concentration was determined using a BCA assay kit. Samples were prepared by adding 4× SDS loading buffer to the proteins and boiling for 10 min. Proteins (20 μg of total protein) were separated on SDS-PAGE and transferred to PVDF membranes. The membranes were blocked with rapid protein-free blocking solution for 15 min, washed three times with 1×TBST for 10 min each time, and cut according to the molecular weight. The membranes were separately incubated overnight at 4°C on a shaker with primary antibodies against GAPDH (1:10,000), VEGFA (1:1000), PI3K p110 Beta (1:1000), p-PI3K (1:500), AKT (1:1000), p-AKT (1:1000), Bax (1:500), Bcl-2 (1:1000), and Caspase-3 (1:500). After washing three times with 1×TBST for 10 min each time, the membranes were incubated with the corresponding secondary antibodies (1:10,000) at room temperature on a shaker in the dark for 1 h. The membranes were washed three times with 1×TBST for 10 min each time, followed by ECL (1:1) exposure and color development. The gel imaging system images were taken and saved, and Image Lab software (Bio-Rad) was used for grayscale analysis of the bands in the images.

### 2.14 Statistical analysis

The data were analyzed using the Graph Pad Prism 11.0 software. Values were presented as the means ± SD deviation. Differences among multiple groups were compared by one-way analysis of variance (ANOVA) with Dunnett’s post-tests or two-way ANOVA with Bonferroni’s post-tests. *P* < 0.05 or *P* < 0.01 was considered statistically significant.

## 3 Results

### 3.1 Effects of harmine derivative B-9-3 on cell proliferation

**FIGURE 1:**
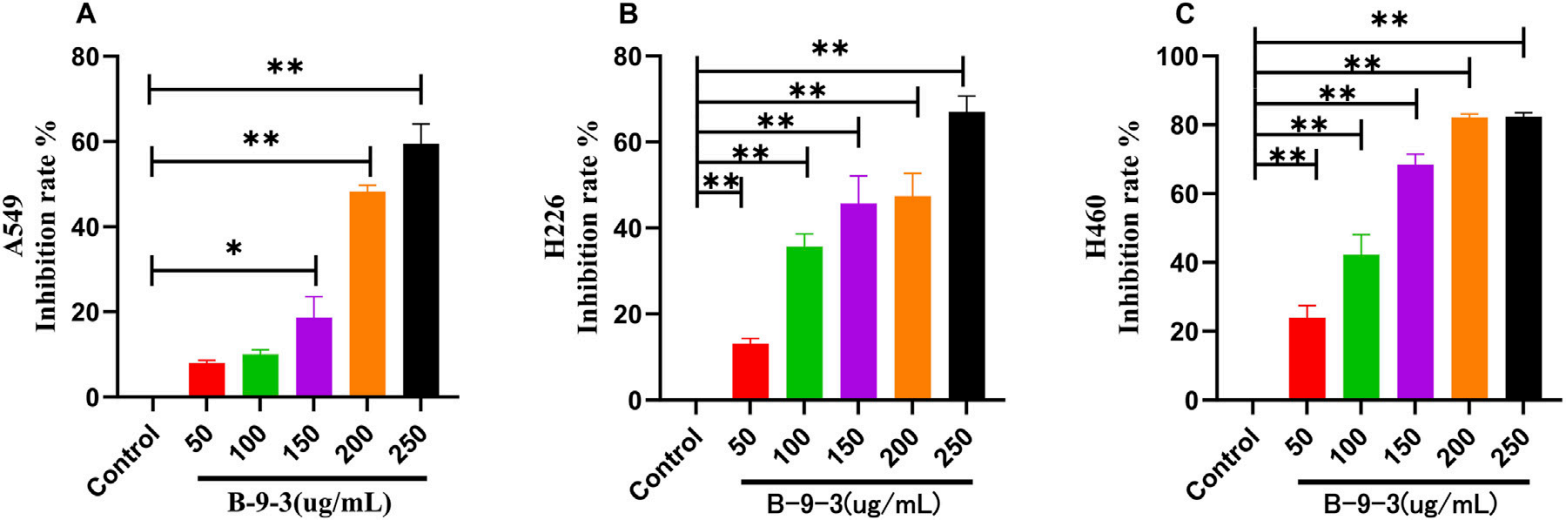
Effect of Harmine derivative B-9-3 on the proliferation of A549, H226 and H460 cells. [**(A–C)**: The number of cells was 1*10^4^/well, Cell inhibition rate after 72 h intervention with B-9-3 administration(%)]. Note: compared with control group, **P < 0.01, *P < 0.05).

The experimental results showed that after 72 h of incubation with B-9-3, the proliferation of A549 cells treated with 250 μg/mL B-9-3 was significantly inhibited compared to the control group, with an IC50 value (%) of 59.42 ± 11.47 (*P* < 0.01). Similarly, the proliferation of H226 cells treated with 200 μg/mL B-9-3 was significantly inhibited, with an IC50 value (%) of 47.46 ± 5.23 (*P* < 0.01), and that of H460 cells treated with 100 μg/mL B-9-3 showed marked inhibition, with an IC50 value of 42.30 ± 5.86 (*P* < 0.01). The rates of inhibition of proliferation in all groups were statistically significant (Figures 1A–C). The experiment was verified by triplicate repetitions.

Based on experimental findings and the proliferation characteristics of tumor cells *in vivo*, as well as the results of CCK-8 assays, a seeding density of 1 × 10^4^ cells/well and concentrations of 50, 100, and 200 μg/mL of B-9-3 were selected for further experiments on the three types of lung cancer cells.

### 3.2 Effect of harmine derivative B-9-3 on cell morphology

**FIGURE 2:**
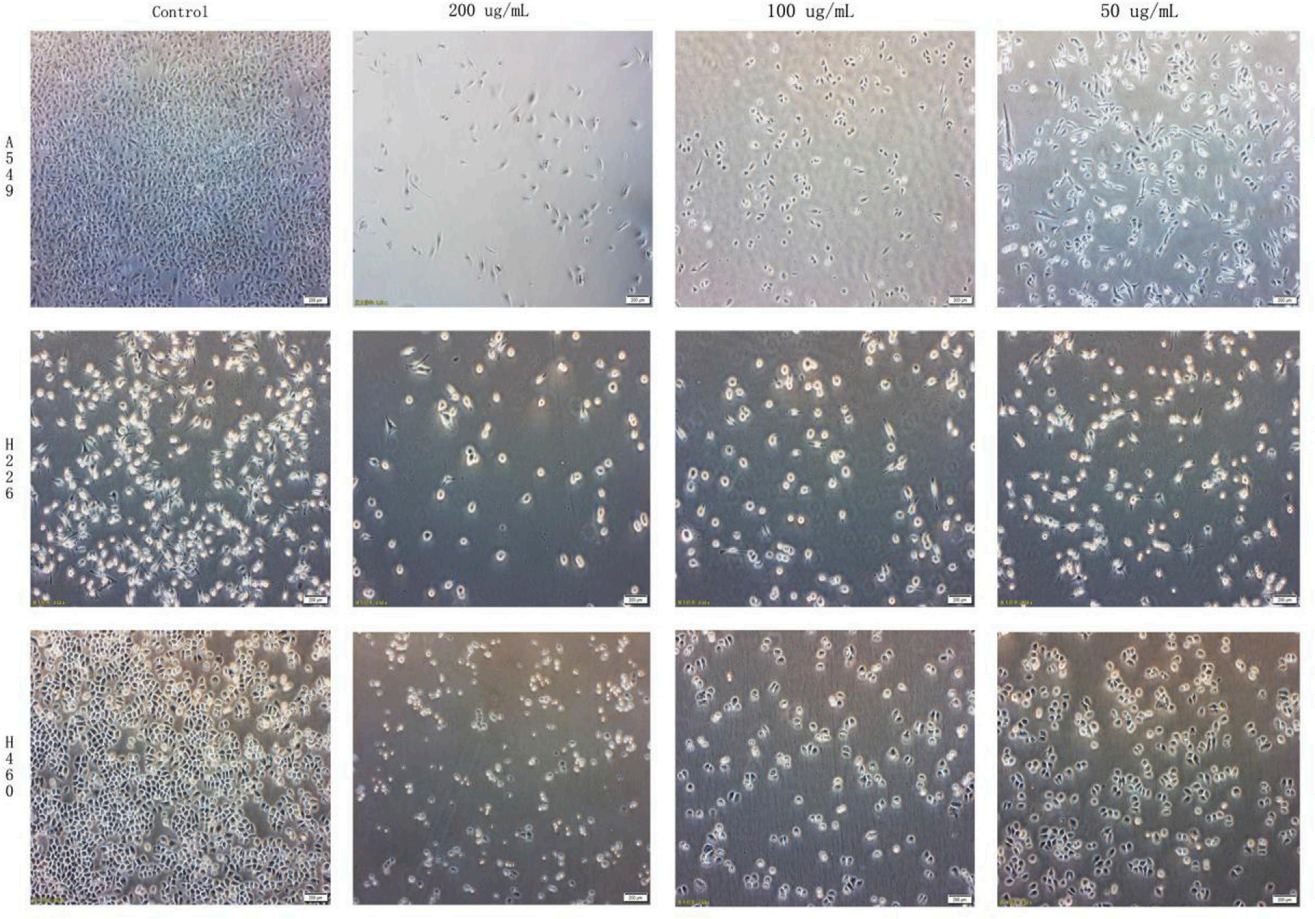
Effects of harmaline derivative B-9-3 on the morphology of A549, H226 and H460 lung cancer cells. After 24 h, cells were treated according to the results of Section 2.4 (50, 100, 200 μg/mL). Note: Scale bar = 200 μm.

Cells in the control group showed good adhesion to the plate surface, exhibiting elongated spindle shapes with clear and smooth edges. However, compared to the control group, cells in all the experimental groups showed evidence of significant cellular damage and death, with varying numbers of suspended cells observed under microscopy. The low and medium concentration groups showed cells with intact morphology but significant numbers of floating and fragmented cells. Cells in the high concentration groups appeared shortened or shriveled into star shapes, with numerous suspended cells and cell fragments visible. This suggests that B-9-3 induced apoptosis in A549, H226, and H460 cells and inhibited cell proliferation (Figure 2). The experiment was verified by triplicate repetitions.

### 3.3 Effect of harmine derivative B-9-3 on cell colony formation

**FIGURE 3:**
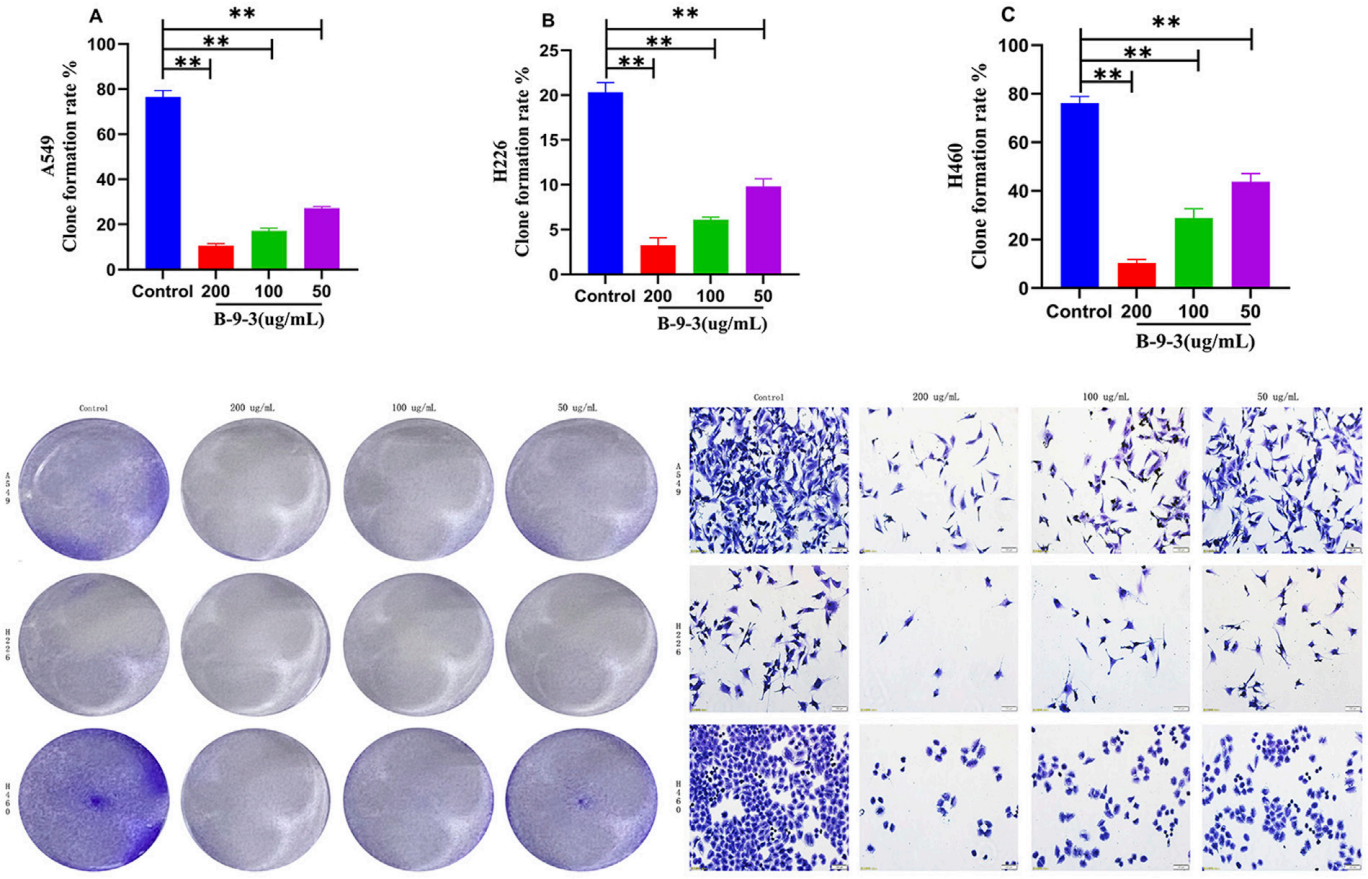
Effect of Harmine derivative B-9-3 on A549, H226 and H460 cell clones. **(A–C)** The number of cells was 2*10^3^/well, Colony forming efficiency after 7 days intervention with B-9-3 administration (%). Note: compared with control group, **P < 0.01, *P < 0.05, the representative images shown on the right in the lower panel correspond to cells within the same colony. Scale bar = 100 μm.

Compared to the control group, colony formation in the three lung cancer cell lines was significantly reduced (*P* < 0.05). With increasing concentrations of B-9-3, the number of colonies formed in each concentration group decreased significantly (*P* < 0.01). Specifically, the number of colonies formed by the A549, H226, and H460 cells in the treated groups was markedly reduced compared to the control group. This result indicates that B-9-3 significantly inhibited colony formation by A549, H226, and H460 cells (Figure 3). The experiment was verified by triplicate repetitions.

### 3.4 Effect of harmine derivative B-9-3 on cell motility

**FIGURE 4:**
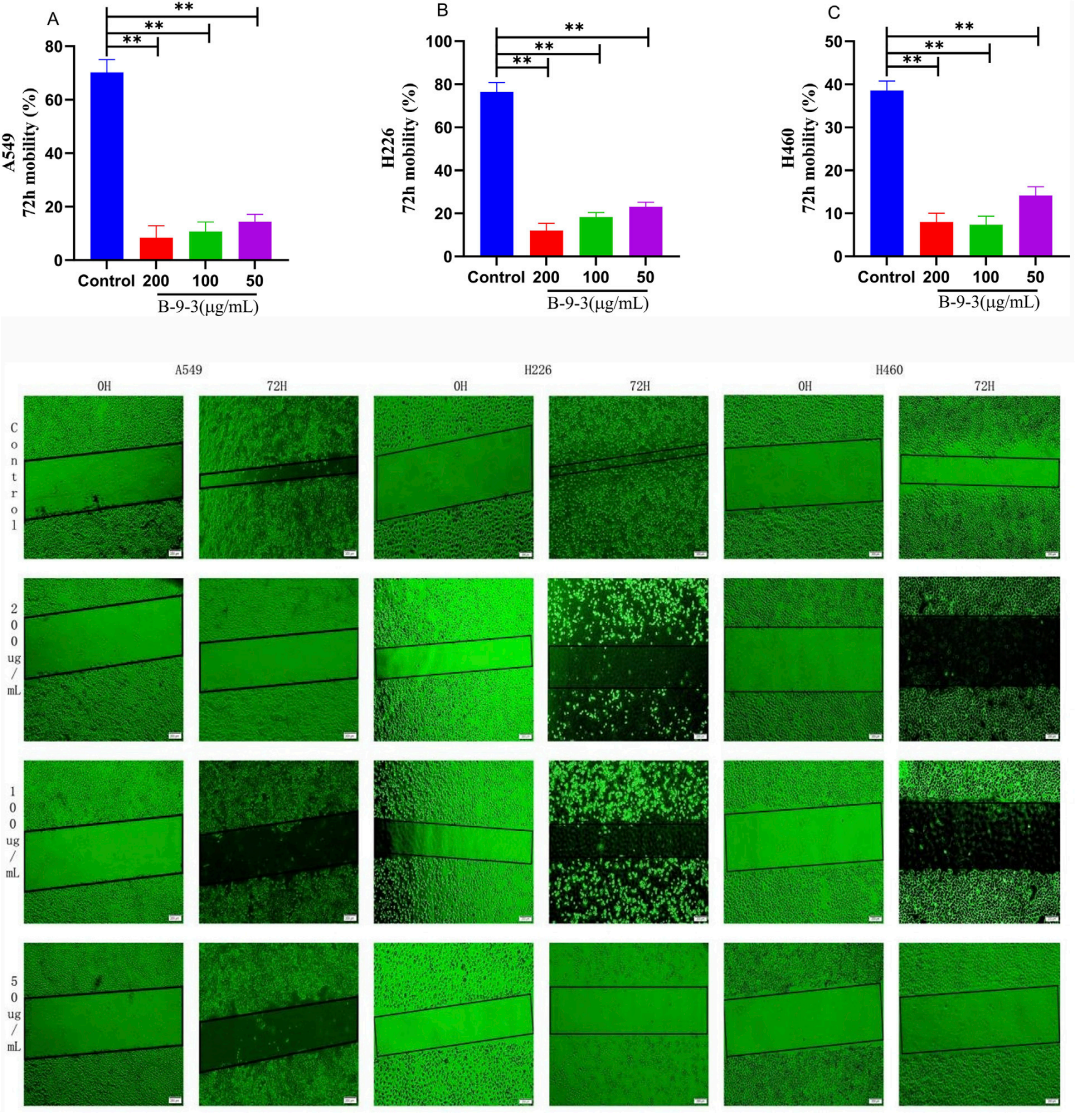
Effects of Harmine derivative B-9-3 on the movements of A549, H226 and H460 cells. **(A–C)** The number of cells was 2*10^5^/well, Cell migration rate after 0 h, 24 h, 72 h intervention with B-9-3 administration (%). Note: compared with control group, **P < 0.01, *P < 0.05, scale bar = 200 μm.

Compared to the control groups, of all three lines treated with B-9-3 (50, 100, 200 μg/mL) showed significantly wound healing after 72 h (*P* < 0.01) (Figure 4). The experiment was verified by triplicate repetitions.

### 3.5 Effect of harmine derivative B-9-3 on cell invasion and migration

The results of the Transwell invasion and migration assays indicated that the percentages of invasion and migration of A549 cells decreased (9.73, 32.11, and 53.62% for invasion; 11.15, 20.69, and 35.57% for migration) in the high, medium, and low B-9-3 concentration groups (200, 100, 50 μg/mL), respectively. Similar reductions were observed for H226 cells (9.64, 31.96, and 53.38% for invasion; 7.09, 16.93, and 25.59% for migration) and H460 cells (16.73, 38.95, and 52.29% for invasion; 9.58, 23.62, and 41.51% for migration). These differences were statistically significant (*P* < 0.01).

**FIGURE 5:**
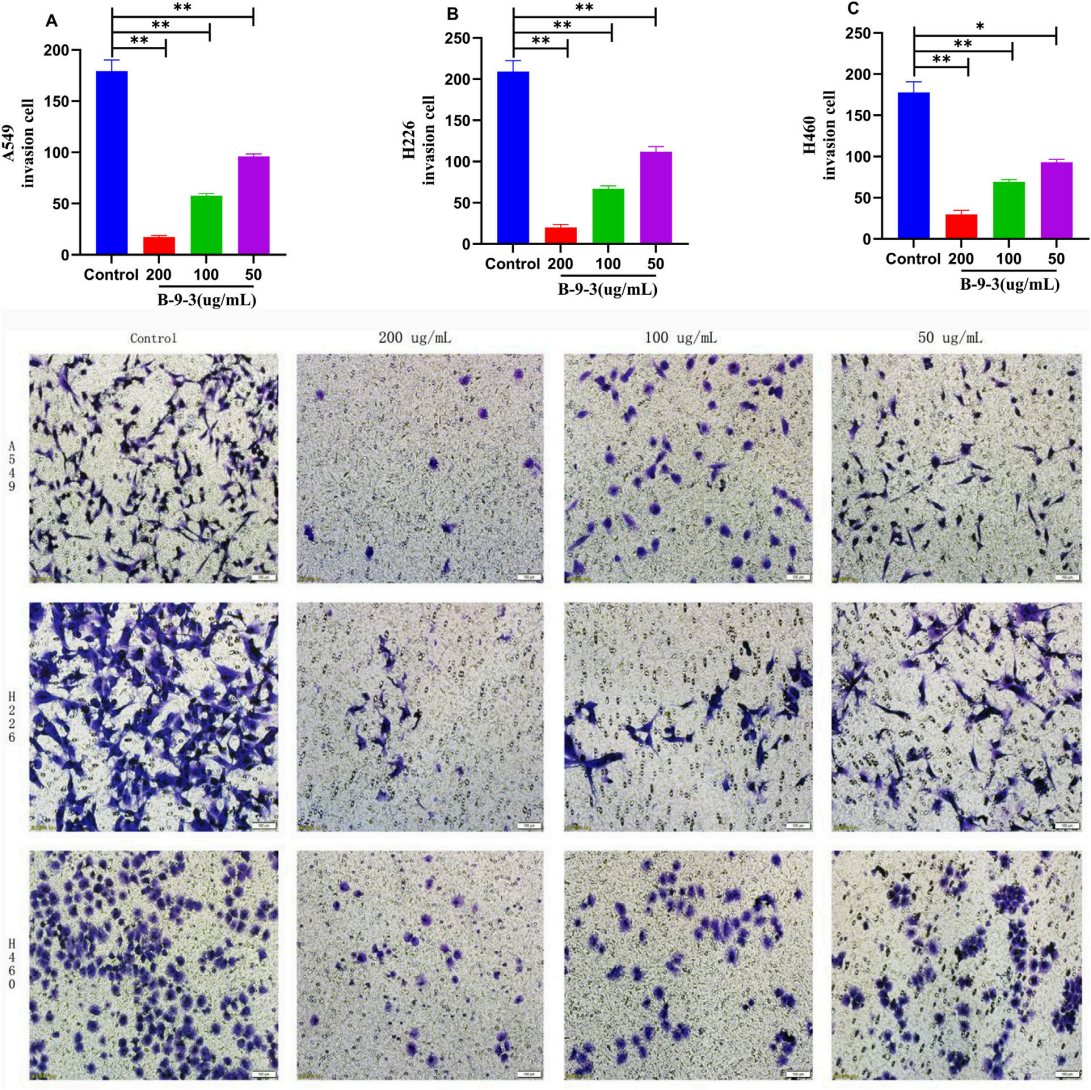
Effects of Harmine derivative B-9-3 on the Invasion ability of A549, H226 and H460 cells. **(A–C)** The number of cells was 2*10^5^/well, percentage of invasive cells after 48 h intervention with B-9-3 administration (%). Note: compared with control group, **P < 0.01, *P < 0.05, scale bar = 100 μm.

**FIGURE 6:**
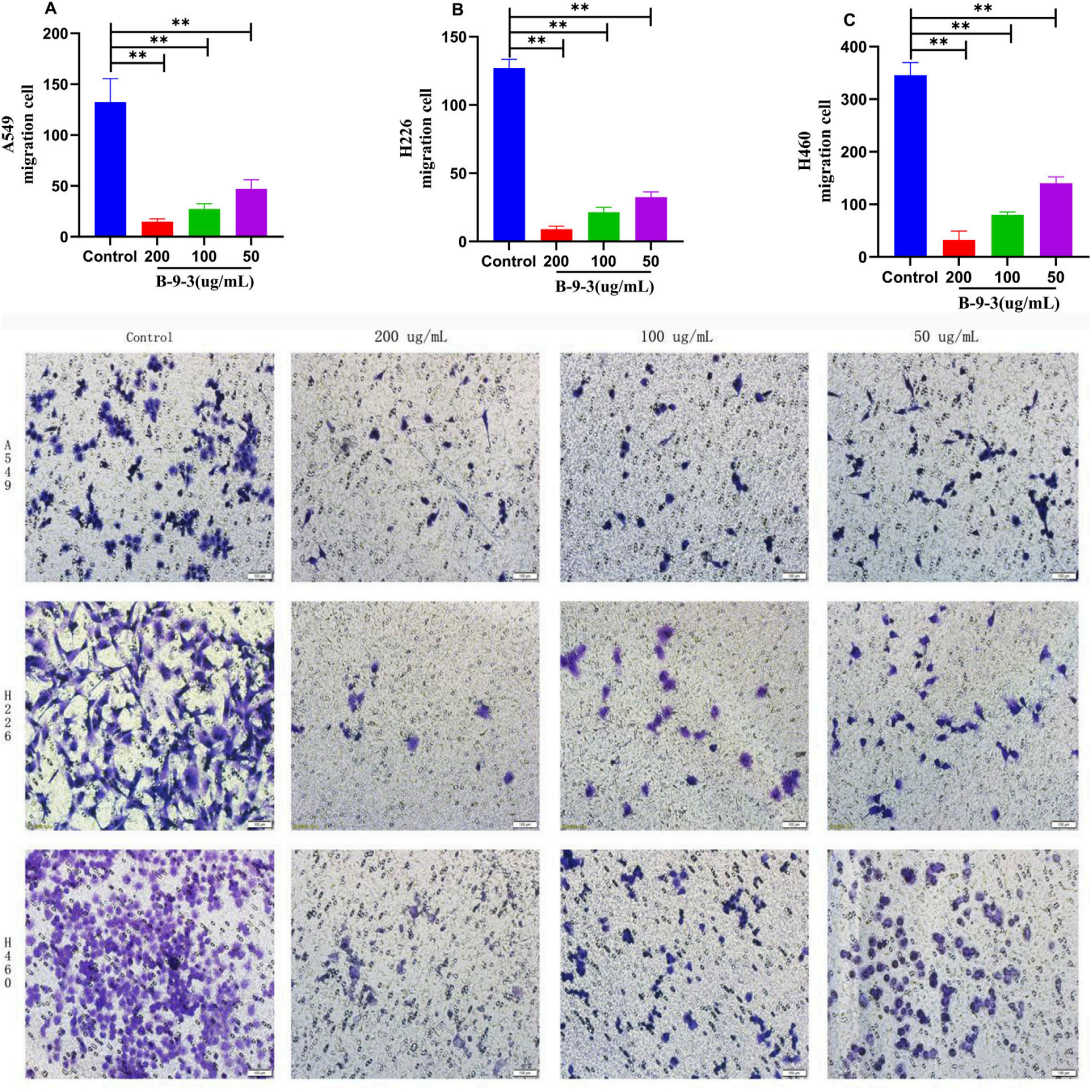
Effects of Harmine derivative B-9-3 on the migration ability of A549, H226 and H460 cells. **(A–C)** The number of cells was 2*10^5^/well, percentage of migratory cells after 48 h intervention with B-9-3 administration (%). Note: compared with control group, **P < 0.01, *P < 0.05, scale bar = 100 μm.

These data indicate that 48 h after B-9-3 treatment, the numbers of invading and migrating cells decreased significantly compared to the control group. B-9-3 significantly inhibited the invasion and migration capabilities of A549, H226, and H460 lung cancer cells, with the number of cells penetrating the membrane in the B-9-3 concentration groups being lower than that in the control groups (*P* < 0.01) (Figures 5, 6). The experiment was verified by triplicate repetitions.

### 3.6 Effect of harmine derivative B-9-3 on cell apoptosis detected by Hoechst 33258 staining

Hoechst staining was utilized to evaluate the nuclear morphology of cells in each group and determine whether apoptosis had occurred. In Hoechst stained samples, normal cell nuclei appeared with regular shapes, uniform staining intensity, and a pale blue color. By contrast, apoptotic cell nuclei displayed irregular shapes, often crescent-shaped or fragmented, and emitted an intense blue fluorescence due to chromatin condensation.

**FIGURE 7:**
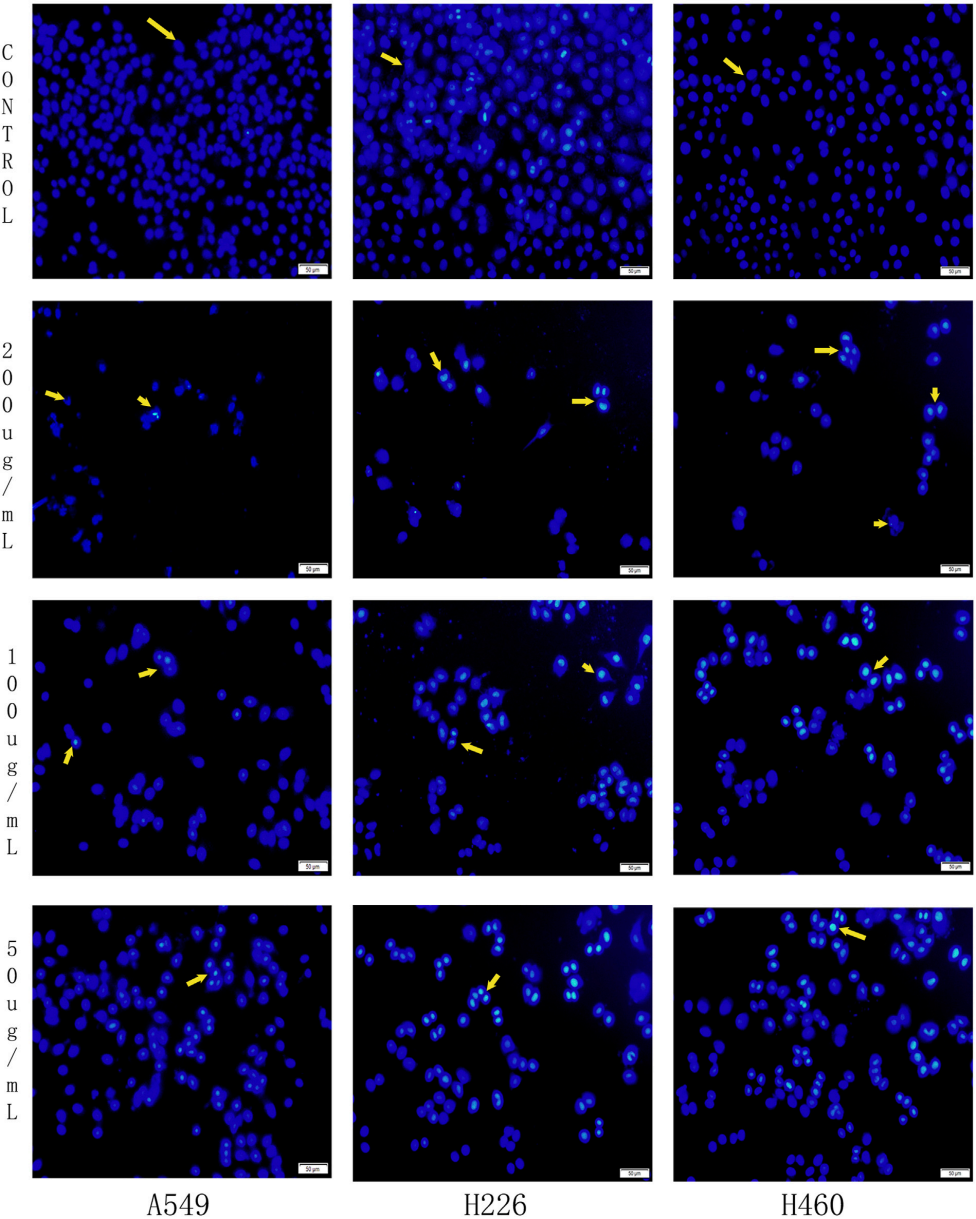
Effects of Harmine derivative B-9-3 on Hoechst 33258 in A549, H226 and H460 cells. Note:Scale bar = 50 μm.

The experimental results demonstrated that the nuclei of the control group cells (A549, H226, and H460) exhibited a uniform light blue color and were predominantly round or oval in shape, indicating the absence of significant apoptosis. In contrast, following treatment with low, medium, and high concentrations of B-9-3 (50, 100, and 200 μg/mL), some cell nuclei displayed intense blue fluorescence, crescent-shaped or fragmented morphology, and a reduction in cell count, suggesting the induction of apoptosis. Notably, in the high-concentration B-9-3 group (200 μg/mL), the nuclei exhibited markedly enhanced blue fluorescence and a significantly reduced cell count (Figure 7).

### 3.7 Effect of harmine derivative B-9-3 on cell apoptosis detected by AO/EB staining

Mitochondrial apoptosis represents a key pathway in the process of cell apoptosis. To further investigate the promoting effects of the Harmine derivative B-9-3 on apoptosis, A549, H226, and H460 cells were treated with B-9-3 for 72 h under different concentration conditions and subsequently stained using the AO/EB staining method. Cell morphology was examined under a fluorescence microscope. Based on the overall morphological features and plasma membrane integrity, the cells were classified into three categories: necrotic cells (red), apoptotic cells (yellow/orange), and viable cells (green).

**FIGURE 8:**
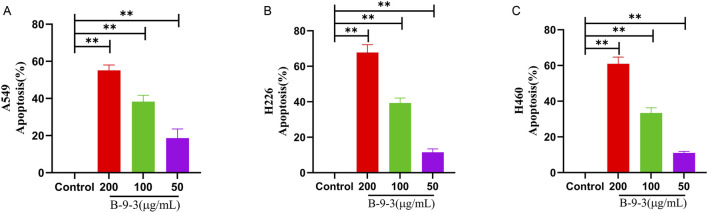
Effects of Harmine derivative B-9-3 on apoptosis of A549, H226 and H460 cells. **(A–C)** The number of cells was 1*10^6^/well, the rate of apoptotic and necrotic cells after intervention with B-9-3 administration(%). Note: compared with control group, **P < 0.01, *P < 0.05.

The experimental results demonstrated that, compared with the control group, following treatment with low, medium, and high concentrations of B-9-3 (50, 100, and 200 μg/mL), an increase in dose was associated with enhanced apoptosis in A549, H226, and H460 cells. This was accompanied by a significant rise in the number of necrotic and apoptotic cells, as well as marked morphological changes in cell structure (P < 0.01) (Figure 8).

**FIGURE 9:**
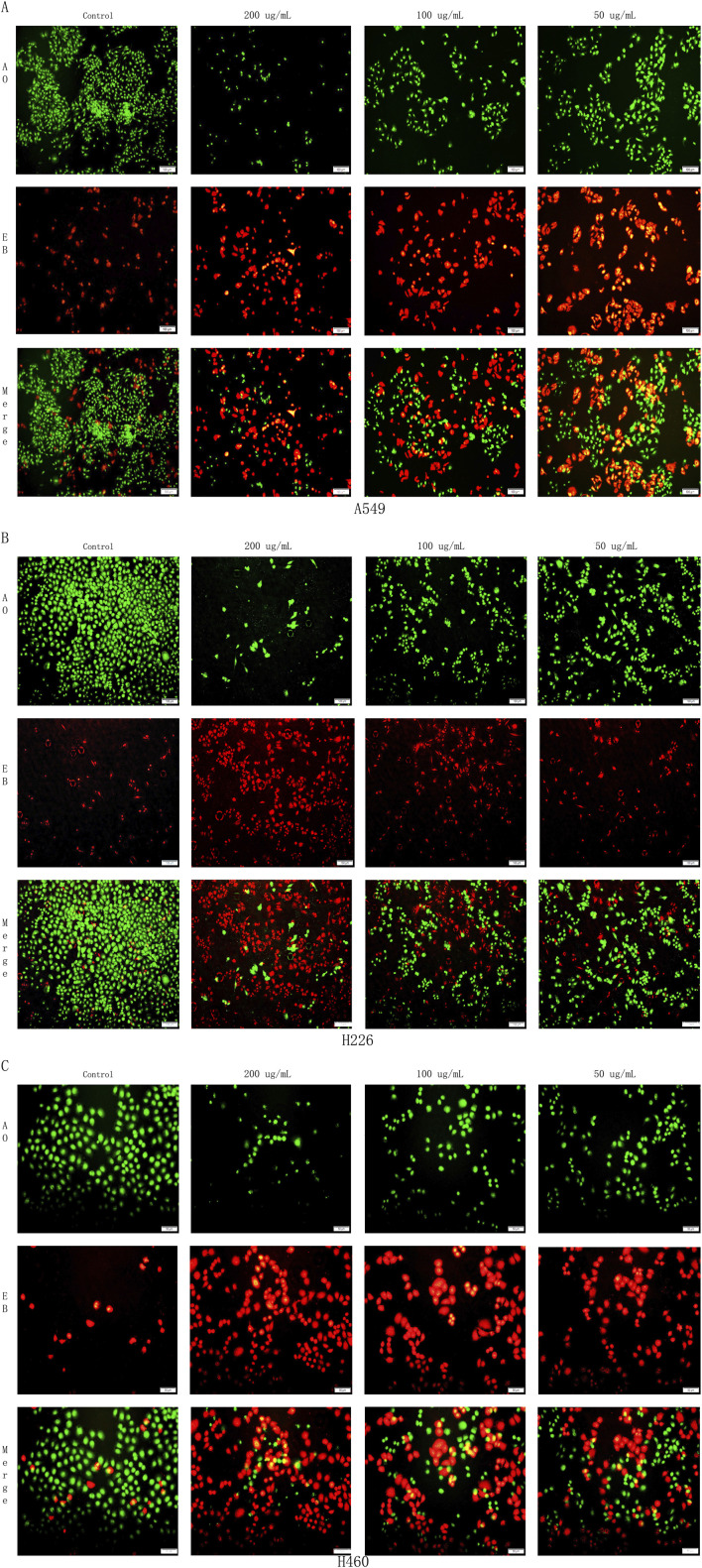
Effects of Harmine derivative B-9-3 on apoptosis of A549, H226 and H460 cells. **(A)** Effects of Harmine derivative B-9-3 on apoptosis of A549; **(B)** Effects of Harmine derivative B-9-3 on apoptosis of H226; **(C)** Effects of Harmine derivative B-9-3 on apoptosis of H460. Note: Scale bar = 100 μm.

The overall morphology of the cells in the A549 control group was intact and displayed an irregular long spindle shape. The overall morphology of the cells in the H226 control group was intact and presented an irregular quadrilateral shape. The overall morphology of the cells in the H460 control group was intact and exhibited an irregular form. Compared with the control groups of the three lung cancer cells, in the low-concentration B-9-3 group: part of the cell morphology was shrunken and necrotic, and apoptotic cells could be observed upon staining. In the medium-concentration B-9-3 group: a small number of cell morphologies were shrunken and necrotic, and the numbers of apoptotic and necrotic cells were significantly increased compared with the low-concentration group, aggregating on one side of the cells in the form of granules and fragments. In the high-concentration B-9-3 group: the cell morphology was shrunken and presented as spherical, irregular sheet-like shapes, and the number of viable cells was significantly reduced, with a large number of apoptotic and necrotic cells (Figures 9A,B,C).

### 3.8 Effect of harmine derivative B-9-3 on cell apoptosis detected by flow cytometry

**FIGURE 10:**
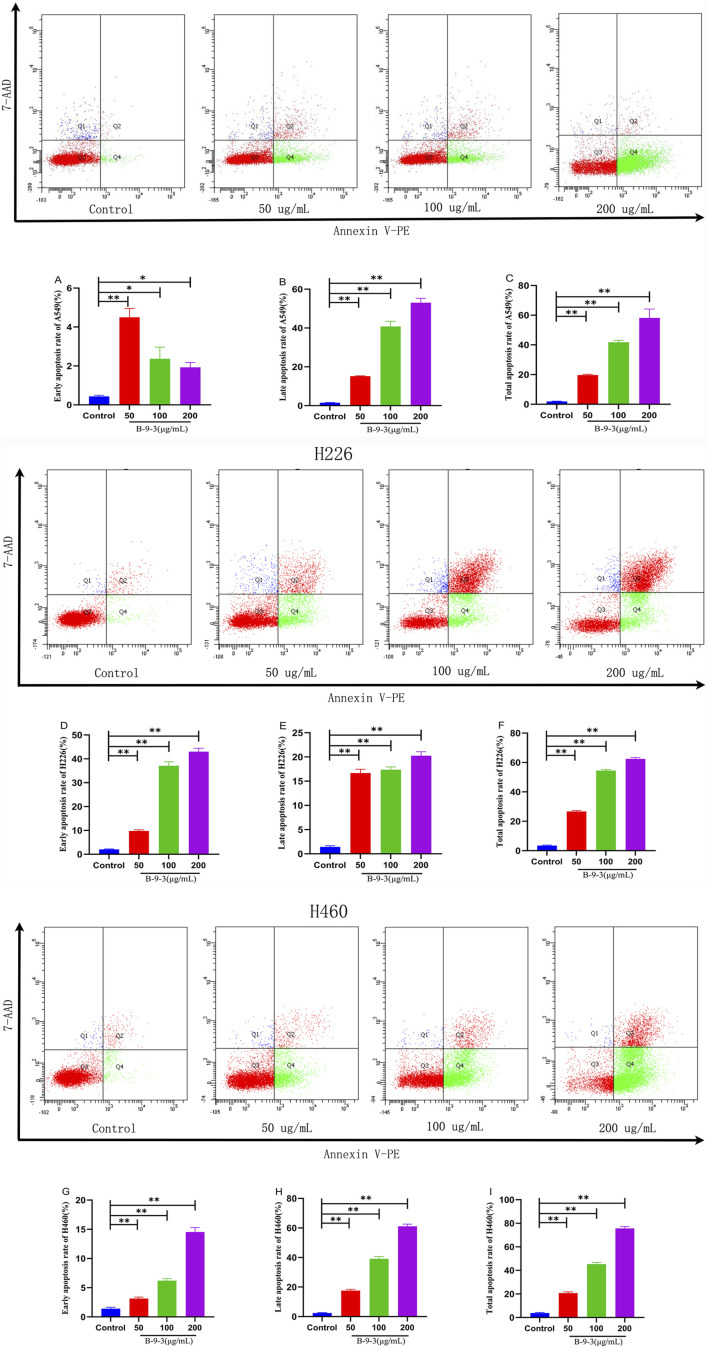
Detection of apoptosis in A549, H226 and H460 cells by Annexin V-PE/7-AAD staining. **(A–C)** Early apoptosis rate, late apoptosis rate, total apoptosis rate of A549; **(D–F)** Early apoptosis rate, late apoptosis rate, total apoptosis rate of H226; **(G–I)** Early apoptosis rate, late apoptosis rate, total apoptosis rate of H460. Q1 The cells in this region are either naked nuclei or cells with mechanical damage. Q2 The cells in this region are either late apoptotic cells or necrotic cells. Q3 The cells in this region are viable cells. Q4 The cells in this region are early apoptotic cells. Note: compared with control group, **P < 0.01, *P < 0.05, One-way analysis of variance was used for comparisons among multiple groups. X-axis: Annexin V-PE, Y-axis: 7-AAD.

Flow cytometry analysis of A549, H226, and H460 cells treated with B-9-3 at low, medium, and high concentrations (50, 100, 200 μg/mL) for 72 h revealed total apoptosis rates of 19.67, 43.07, and 58.17% for A549 cells, respectively, 26.67, 54.47, and 62.40% for H226 cells, respectively, and 20.66, 45.30, and 75.60% for H460 cells, respectively. Compared to the control groups of the three cell lines, the B-9-3 low and medium concentrations showed significant differences in the early and late apoptosis rates in A549 cells (*P* < 0.01), with significantly increased total apoptosis rates (t = 17.77, 41.17, 56.27, *P* < 0.01). B-9-3 treatment at different concentrations also led to significantly different early and late apoptosis rates in H226 cells (*P* < 0.01), with significantly increased total apoptosis rates (t = 23.20, 51.20, 58.93, *P* < 0.01). Similarly, medium and high concentrations of B-9-3 resulted in markedly increased early and late apoptosis rates in H460 cells (*P* < 0.01), with significantly increased total apoptosis rates (t = 16.87, 41.5, 71.8, *P* < 0.01) (Figure 10). The experiment was verified by triplicate repetitions.

### 3.9 Effect of harmine derivative B-9-3 on expression levels of apoptosis-related genes

The amplification efficiencies of Bax, Bcl-2, Caspase-3, and β-actin were all above 95%, with single-peaked dissolution curves and strong specificity. Subsequent sample amplification analysis was performed using the 2^−△△Ct^ method with β-actin used as an internal reference to calculate the relative expression levels of Bax, Bcl-2, and Caspase-3 mRNA in A549, H226, and H460 cells.

**FIGURE 11:**
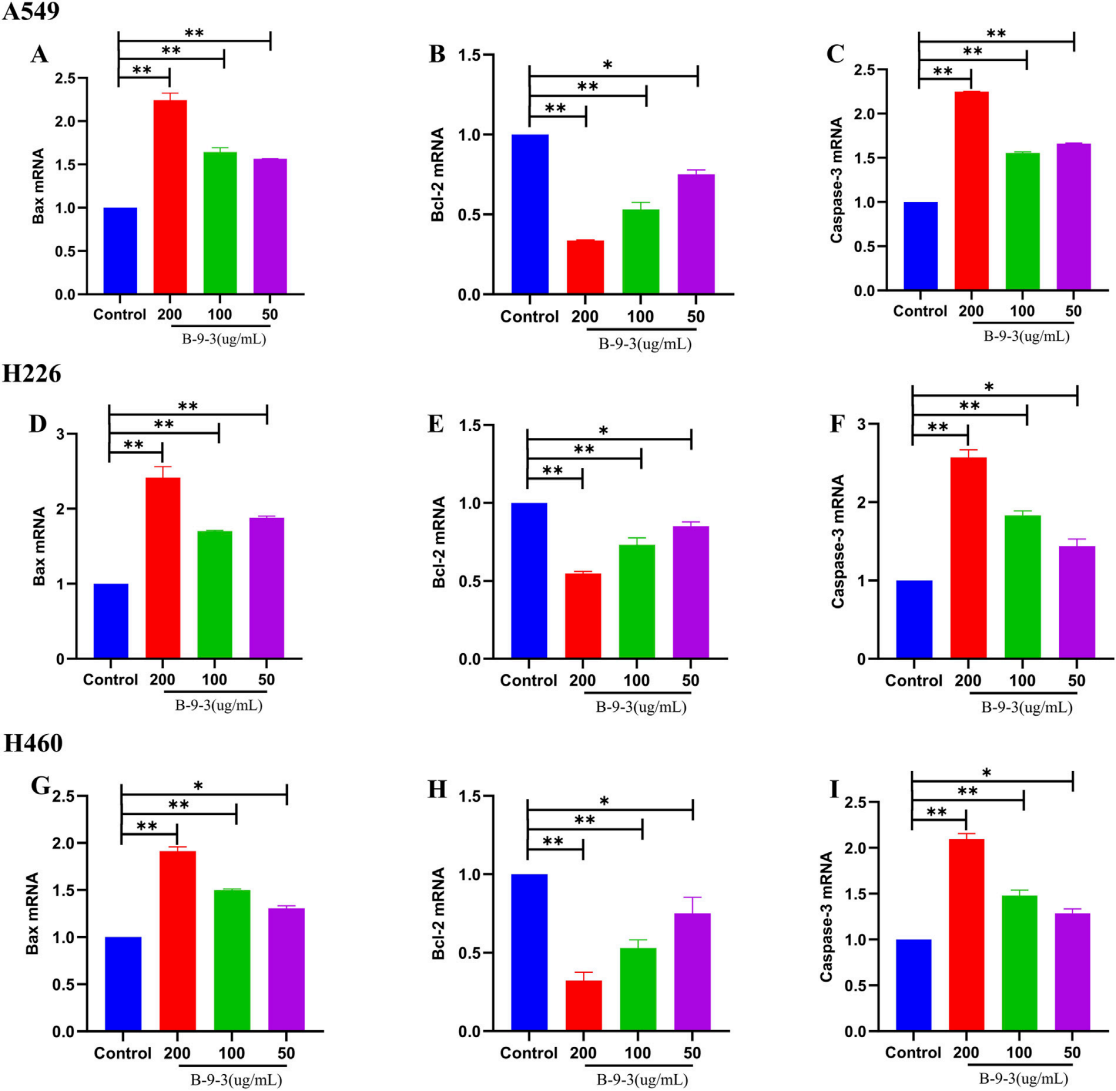
Effect of Harmine derivative B-9-3 on Bax, Bcl-2, Caspase-3 mRNA expression of A549, H226 and H460 cells. **(A–C)** Bax, Bcl-2, Caspase-3 mRNA expression of A549; **(D–F)** Bax, Bcl-2, Caspase-3 mRNA expression of H226; **(G–I)** Bax, Bcl-2, Caspase-3 mRNA expression of H460. Note: compared with control group, **P < 0.01, *P < 0.05.

Compared with the control groups of the three types of cells, after pretreatment with low, medium, and high concentrations of B-9-3 (50, 100, 200 μg/mL), the expression levels of Bax and Caspase-3 mRNA in the three types of cells increased significantly (P < 0.05 or P < 0.01), while the levels of Bcl-2 mRNA were significantly decreased (P < 0.05 or P < 0.01) (Figure 11). The experiment was verified by triplicate repetitions.

### 3.10 effects of harmine derivative B-9-3 on protein expression of VEGFA, PI3K p110 Beta, p-PI3K, AKT, p-AKT, Bax, Bcl-2, and Caspase-3

**FIGURE 12:**
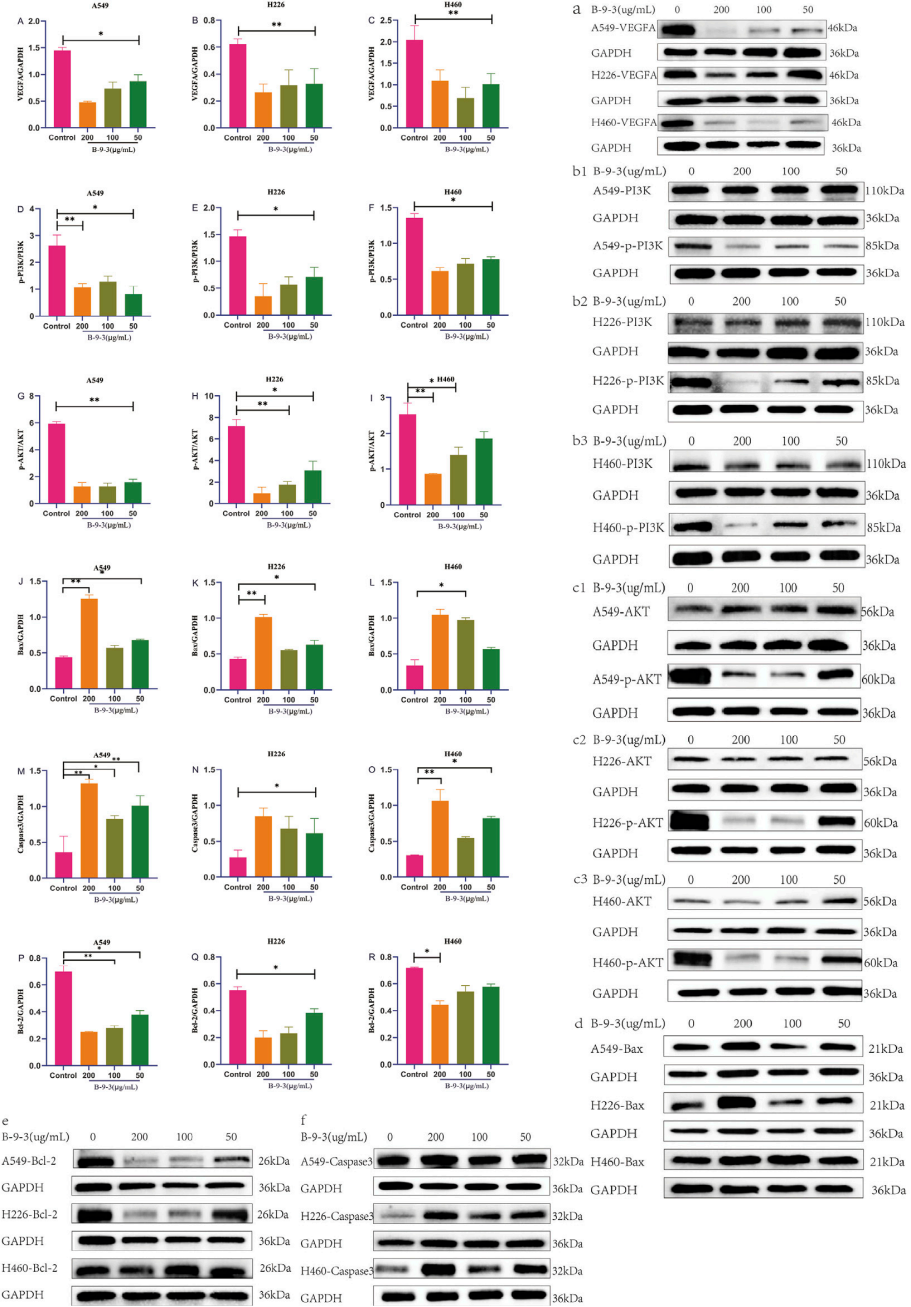
Effects of Harmine Derivative B-9-3 on Protein Expression of VEGFA, PI3K p110 Beta, p-PI3K, AKT, p-AKT, Bax, Bcl-2, and Caspase-3 in A549, H226, and H460 cells. **(A–C) **a Effect of Harmine derivative B-9-3 on expressions of VEGFA protein in A549, H226 and H460 cells. (D–F) b1–b3 Effect of Harmine derivative B-9-3 on expressions of PI3K/p-PI3K protein in A549, H226 and H460 cells. (G–I) c1–c3 Effect of Harmine derivative B-9-3 on expressions of AKT/p-AKT protein in A549, H226 and H460 cells. **(J–L)** d Effect of Harmine derivative B-9-3 on expressions of Bax protein in A549, H226 and H460 cells. **(M–O)** e Effect of Harmine derivative B-9-3 on expressions of Caspase-3 protein in A549, H226 and H460 cells. **(P–R)** f Effect of Harmine derivative B-9-3 on expressions of Bcl-2 protein in A549, H226 and H460 cells. Note: compared with control group, **P < 0.01, *P < 0.05.

Western blotting results showed that compared with the control groups of the three types of cells, after pretreatment with low, medium, and high concentrations of B-9-3 (50, 100, 200 μg/mL), the protein levels of VEGFA were significantly reduced (P < 0.05 or P < 0.01) in each concentration group. These findings indicate that B-9-3 had an inhibitory effect on angiogenesis in the three lung cancer cell lines, possibly related to the reduction in the VEGFA protein levels (Figures 12A–C,a).

In A549 cells, the protein levels of PI3K/p-PI3K were observed to be significantly decreased in each concentration group (P < 0.05 or P < 0.01), while in H226 cells, the protein levels of PI3K/p-PI3K were significantly decreased in the high and medium concentration groups (P < 0.05) and in H460 cells, the protein levels of PI3K/p-PI3K were markedly reduced in each concentration group (P < 0.05 or P < 0.01). These experiments indicate that B-9-3 may downregulate PI3K/p-PI3K protein expression in the three lung cancer cell lines (Figures 12D–F,b1–b3).

In A549 cells, the protein levels of AKT/p-AKT were markedly reduced in each concentration group (P < 0.01), while in H226 cells, AKT/p-AKT protein expression was significantly decreased in each concentration group (P < 0.01 or P < 0.05) and in H460 cells, the protein levels of AKT/p-AKT were significantly decreased in the high and medium concentration groups (P < 0.05 or P < 0.01). These results indicate that B-9-3 may downregulate AKT/p-AKT protein levels in the three lung cancer cell lines (Figures 12G–I,c1–c3).

In A549 cells, the protein levels of Bax were found to be significantly increased in the high and low concentration groups (P < 0.01 or P < 0.05), while in H226 cells, Bax protein levels were markedly increased in the high and low concentration groups (P < 0.01 or P < 0.05) and in H460 cells, Bax levels were elevated in the high and medium concentration groups (P < 0.05). These data indicate that B-9-3 treatment may lead to the upregulation of the pro-apoptotic protein Bax in the three lung cancer cell lines (Figures 12J–L,d).

The protein levels of Caspase-3 were significantly increased in each concentration group in A549 cells, (P < 0.01 or P < 0.05), as well as in H226 cells (P < 0.05), while in H460 cells, Caspase-3 levels were only elevated in the high and low concentration groups (P < 0.05 or P < 0.01). This suggests that B-9-3 upregulated the pro-apoptotic protein Caspase-3 in the three lung cancer cell lines (Figures 12M–O,e).

In terms of Bcl-2 protein expression, both A549 and H226 cells showed markedly reduced levels of the protein in each concentration group (P < 0.01 or P < 0.05 for A549, P < 0.05 for H226), while levels in H460 cells were only significantly decreased in the high concentration group (P < 0.05). These findings indicate that B-9-3 downregulated the anti-apoptotic protein Bcl-2 in the three lung cancer cell lines (Figures 12P–R,f ).

## 4 Discussion

The uncontrolled proliferation of tumor cells is one of the core features of malignant tumors, and inhibition of proliferation is an important strategy in anti-tumor therapy (Meixin and Youran, 2024). The derivative B-9-3 of harmine exhibited a significant inhibitory effect on the proliferation of A549, H226, and H460 cells at a low concentration (50 μg/mL), with the IC50 values being 59.42% ± 11.47%, 47.46% ± 5.23%, and 42.30% ± 5.86% respectively. Moreover, this inhibitory effect was dose-dependent (P < 0.01). Furthermore, B-9-3 significantly reduced the colony-forming ability of all three cell lines, indicating that it not only inhibits monolayer cell proliferation but also impedes clonal expansion of tumor cells. This result is consistent with the study by Zhang (2020), which indicates that the derivatives of paucimarone inhibit tumor proliferation by suppressing the expression of cell cycle-related proteins, such as Cyclin D1. However, in contrast to the study by Ji et al. (2016), which demonstrated the inhibitory effects of liposomal harmine on cervical cancer cells, B-9-3 exhibits a more pronounced inhibitory effect on NSCLC. This enhanced efficacy may be attributed to the improved targeting properties resulting from its structural modifications (Li et al., 2017).

Tumor metastasis is a primary cause of clinical treatment failure, with invasion and migration being critical steps in the metastatic process (Ganesh and Massagué, 2021; hanahan and Weinberg, 2021). In this study, both scratch assays and Transwell assays demonstrated that B-9-3 dose-dependently inhibited the scratch healing rate and invasion/migration capability of three NSCLC cell lines. Specifically, B-9-3 achieved a maximum inhibition rate of 53.62% for invasion and 35.57% for migration (P < 0.01). This finding is similar to the inhibitory effect of the HDAC inhibitor HBC on the migration of liver cancer cells reported by Miao et al. (2018). However, the innovation of this study resides in clarifying the regulation of B-9-3 on the specific metastatic behavior of lung cancer. Notably, B-9-3 exhibits a particularly significant inhibitory effect on the invasion of H460 cells, with an invasion inhibition rate of 52.29%. This suggests that B-9-3 may exert its effects by targeting unique signaling pathways in highly metastatic lung cancer subtypes, such as those related to epithelial-mesenchymal transition (EMT). Further validation is warranted to confirm these findings.

Apoptosis is the core mechanism of action for anti-tumor drugs, and its regulation depends on the balance between pro-apoptotic factors (Bax, Caspase-3) and anti-apoptotic factors (Bcl-2) (Zhang et al., 2019; Choudhary et al., 2019; Carmeliet, 2015). In this study, multi-dimensional experiments confirmed that B-9-3 dose-dependently induced apoptosis in three types of NSCLC cells. Morphological evidence and Hoechst 33,258 staining revealed that high concentrations of B-9-3 (200 μg/mL) led to dense granular fluorescence and chromatin condensation in the cell nuclei. Acridine orange (AO) staining further distinguished apoptotic cells (orange) from necrotic cells (red), clearly indicating that apoptosis was the predominant mode of cell death.

The results of Western blot and qPCR showed that B-9-3 significantly upregulated the protein/mRNA expression of Bax and Caspase-3, while down-regulating Bcl-2, suggesting that it exerts its effect by activating the mitochondrial apoptotic pathway. This mechanism is different from the model of p53 pathway activation-induced apoptosis proposed by Dai et al. (2012), indicating that B-9-3 may enhance the pro-apoptotic effect through the synergistic action of multiple targets. It is noteworthy that the activation of Caspase-3 by B-9-3 exhibits a significant dose-dependent effect, achieving a total apoptosis rate of 75.60% in H460 cells. This characteristic may be attributed to the enhanced membrane permeability resulting from its structural modification (Ma et al., 2018). Future studies should employ molecular docking experiments to further validate this hypothesis.

Angiogenesis is a driving force for tumor progression, and VEGFA is a core factor regulating angiogenesis (Folkman, 2020; Verheul and Pinedo, 2020). This study found that B-9-3 could significantly downregulate the expression of VEGFA protein in three NSCLC cell lines, suggesting that it cuts off the nutrient supply by inhibiting tumor-derived angiogenic factors. Furthermore, the inhibitory effect of B-9-3 on the PI3K/AKT signaling pathway (reduced levels of p-PI3K/PI3K and p-AKT/AKT proteins) further clarifies its multi-target anti-tumor mechanism. The aberrant activation of the PI3K/AKT pathway is closely linked to tumor cell survival and drug resistance (Costa et al., 2018; Hoxhai and Manning, 2020). By inhibiting the phosphorylation of PI3K and AKT, B-9-3 likely disrupts downstream mTOR-mediated pro-survival signaling, leading to cell cycle arrest (such as G1 phase arrest) and increased apoptosis sensitivity.

The dual-target synergy observed in this study differs from previous studies that exclusively targeted VEGFR2 (Piekarski et al., 2014; Phalen et al., 2013). B-9-3 concurrently inhibits VEGFA and the PI3K/AKT pathway, potentially enhancing therapeutic efficacy through dual mechanisms of “vascular normalization” and “direct tumor cell killing.” This strategy provides a novel approach to overcoming the drug resistance associated with single-agent anti-angiogenesis therapies (Detmar, 2020; McMahon, 2020).

Although this study systematically revealed the anti-NSCLC effect of B-9-3, the following limitations still exist: all current results are based on *in vitro* cell experiments, and the *in vivo* efficacy and toxicity of B-9-3 need to be evaluated through mouse xenograft tumor models. The specific mechanisms underlying the downregulation of VEGFA, such as transcriptional repression or protein degradation, have not yet been fully elucidated. Future studies should integrate ChIP-seq and ubiquitination assays to further investigate these mechanisms. The current cell model included only three NSCLC cell lines. Future studies should extend this work to incorporate primary tumor cells and additional subtypes, such as EGFR-mutant cells, to enhance the generalizability and robustness of the findings.

This study is the first to demonstrate that the dehydroharmine derivative B-9-3 inhibits proliferation, migration, and induces apoptosis in NSCLC cells by dual-targeting the VEGFA and PI3K/AKT pathways. Its multi-targeting properties make it an ideal candidate for combination therapies (e.g., with PD-1 inhibitors or chemotherapy). Future research should focus on structural optimization to further reduce neurotoxicity through modifications of the nine-position substituents. Based on observed differences in cell sensitivity (such as high sensitivity in H460 cells), molecular markers predictive of B-9-3 efficacy (e.g., PI3K mutation status) can be identified to develop diagnostic biomarkers for NSCLC. Conducting pharmacokinetic and toxicological studies will facilitate the translation of B-9-3 into clinical trials, enabling its application in preclinical evaluation.

## 5 Conclusion

In summary, the findings of this study showed that treatment with B-9-3 inhibited cell invasion and metastasis, enhanced apoptosis, and suppressed angiogenesis in A549, H226, and H460 NSCLC cells. Its mechanism may involve regulation of the VEGFA/PI3K/AKT signaling pathway. This finding also substantiates that B-9-3, by down-regulating the expression of VEGFA and inhibiting its binding to receptors, subsequently blocks the initial activation of pro-angiogenic signals. Further modulation of the phosphorylation status within the PI3K/AKT signaling pathway, specifically by reducing AKT phosphorylation levels, disrupts the propagation of downstream pro-survival and proliferative signals, ultimately leading to the suppression of angiogenesis-related gene expression. Thus, it inhibits the proliferation, migration and tubular structure formation of endothelial cells, induces apoptosis of vascular endothelial cells, and destroys the formed microvascular network. In summary, the discovery that B-9-3 inhibits angiogenesis by targeting the VEGFA/PI3K/AKT signaling pathway provides a robust theoretical basis for the development of novel multi-mechanistic anti-cancer agents. Its dual effects directly inducing tumor cell apoptosis and reshaping the tumor microenvironment may represent a promising strategy for NSCLC therapy. Future research should focus on elucidating its *in vivo* efficacy and exploring the potential synergistic effects with current therapeutic modalities.

## Data Availability

The datasets presented in this study can be found in online repositories. The names of the repository/repositories and accession number(s) can be found in the article/Supplementary Material.
