# Correction: Harmine derivative B-9-3 inhibits non-small cell lung cancer via the VEGFA/PI3K/AKT pathway

**DOI:** 10.3389/fphar.2025.1701287

**Published:** 2025-11-21

**Authors:** Yuche Wu, Bing Wang, Xuwen Mao, Wei Chen, Haji Akber Aisa

**Affiliations:** 1 Xinjiang Technical Institute of Physics and Chemistry Chinese Academy of Sciences, Urumqi, Xinjiang, China; 2 Xinjiang Huashidan Pharmaceutical Co., Ltd., Urumqi, Xinjiang, China; 3 The Fourth Affiliated Hospital of Xinjiang Medical University, Urumqi, Xinjiang, China; 4 College of Pharmacy, Xinjiang Medical University, Urumqi, Xinjiang, China

**Keywords:** harmine derivative B-9-3, non-small cell lung cancer, apoptosis, angiogenesis, VEGFA/PI3K/AKT

**Affiliation 4** ‘Xinjiang Huashidan Pharmaceutical Co., Ltd., Urumqi, Xinjiang, China’ was renumbered as Affiliation 2.

Affiliation 2 was erroneously omitted for author Yuche Wu. The **conflict of interest statement** has been corrected to “Authors YW and WC were employed by Xinjiang Huashidan Pharmaceutical Co., Ltd. The remaining authors declare that the research was conducted in the absence of any commercial or financial relationships that could be construed as a potential conflict of interest.”

The original article has been updated.

