# Supplementary material for: Harmine derivative B-9-3 inhibits non-small cell lung cancer via the VEGFA/PI3K/AKT pathway
Source: Front Pharmacol. 2025 May 13;16:1526952. doi: 10.3389/fphar.2025.1526952 (PMC12107193; doi:10.3389/fphar.2025.1526952)
Supplement: Supplementary file 1 [file Supplementaryfile1.docx]

A549-VEGFA


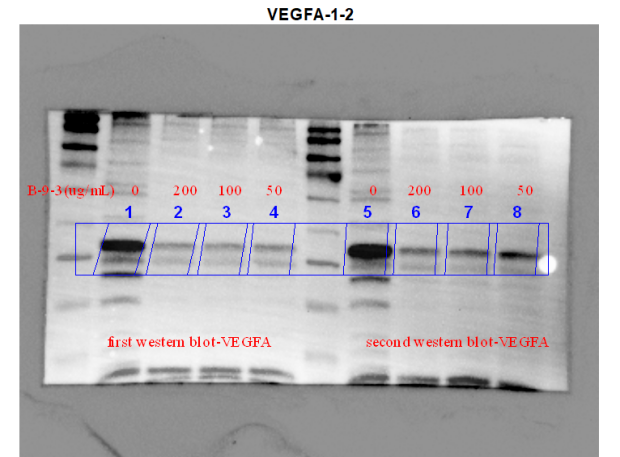

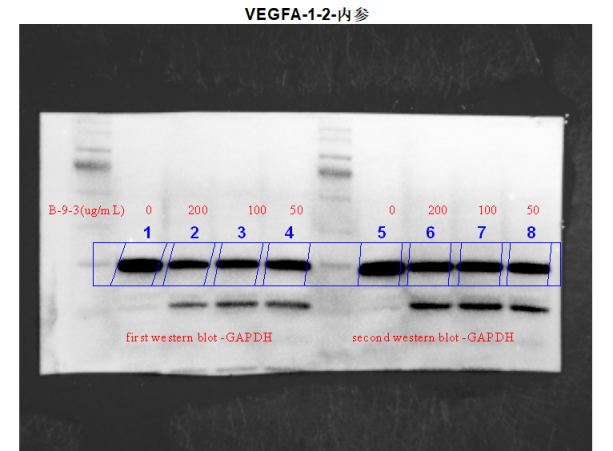


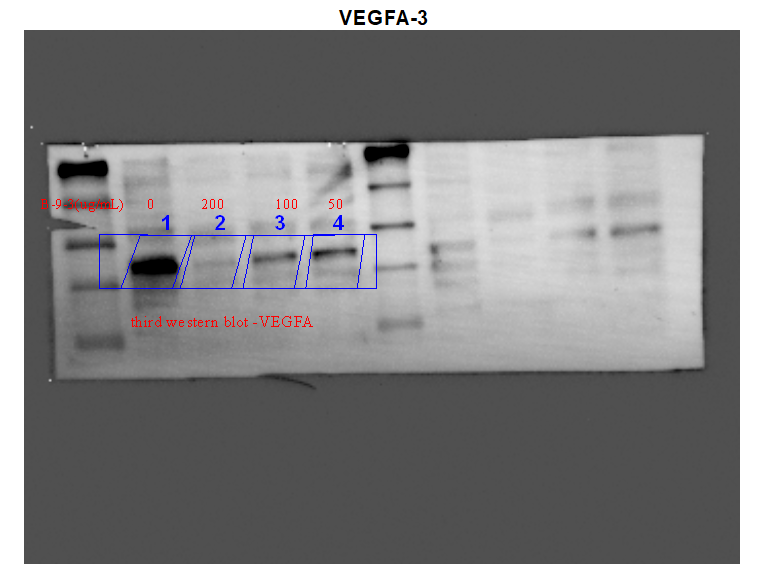


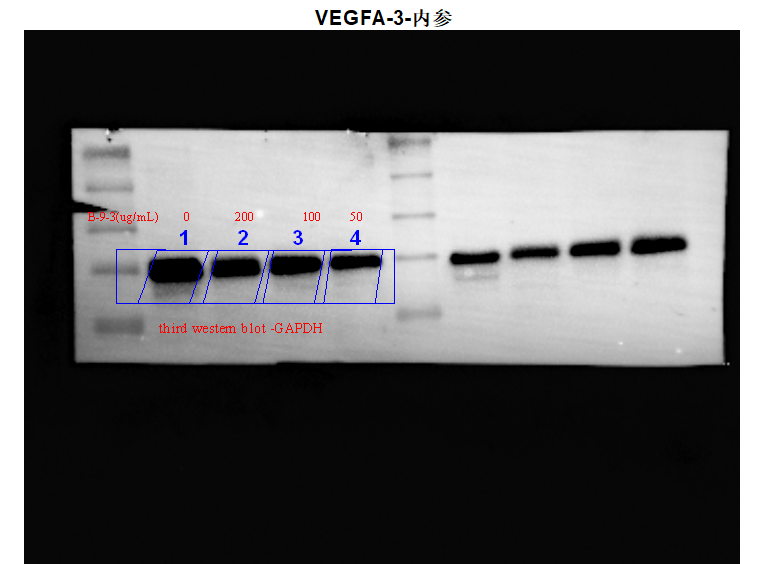


H226-VEGFA


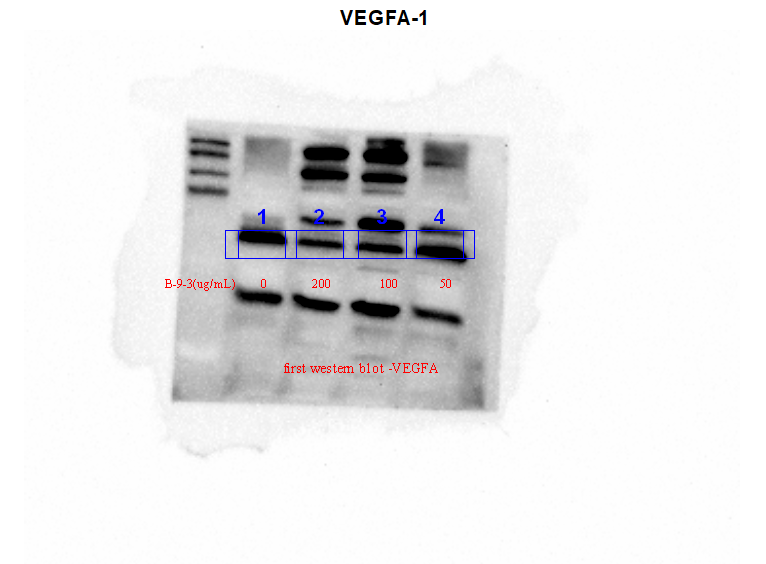

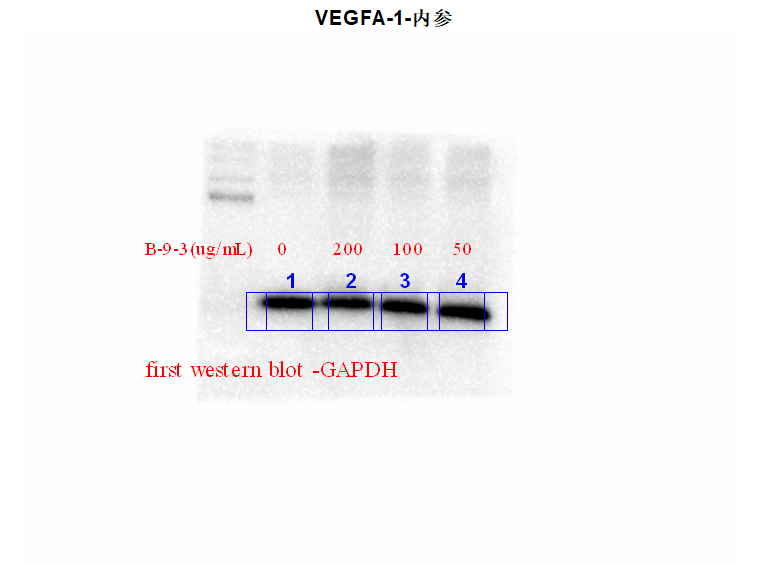


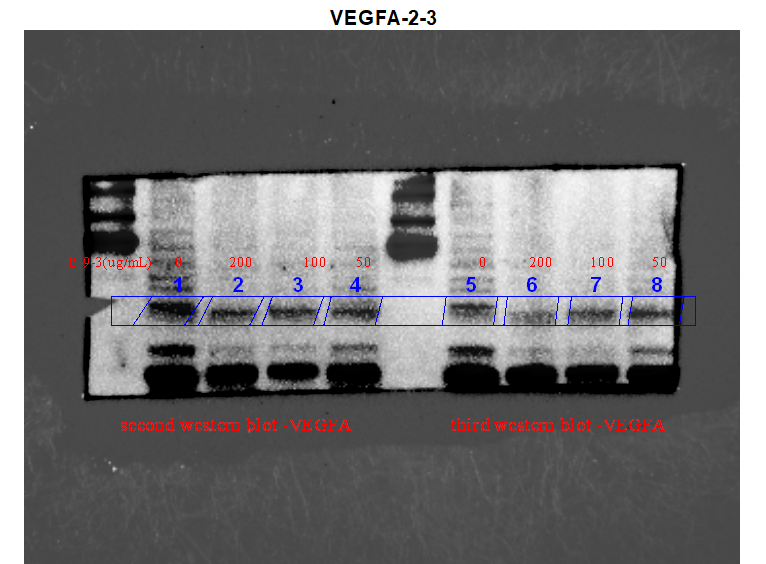


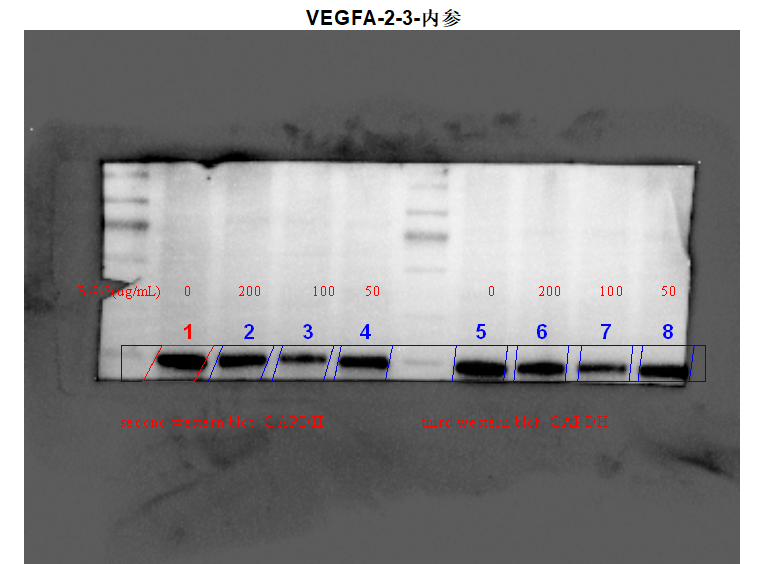


H460-VEGFA


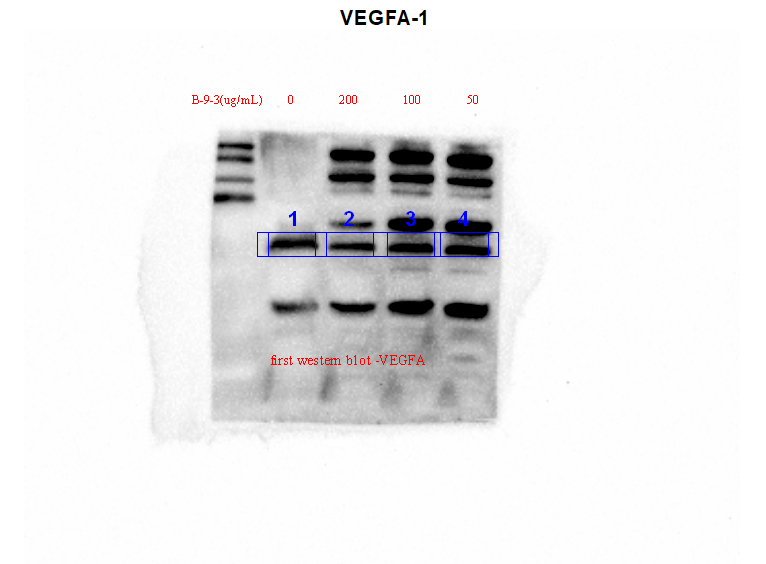

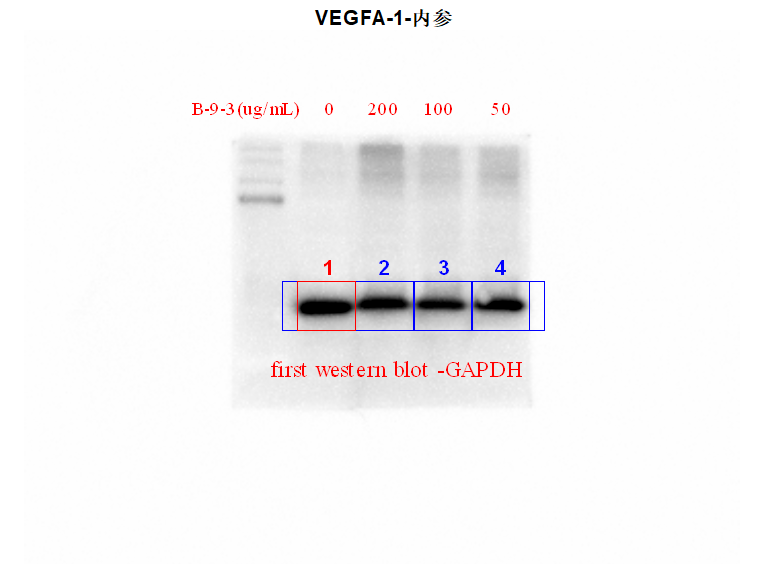


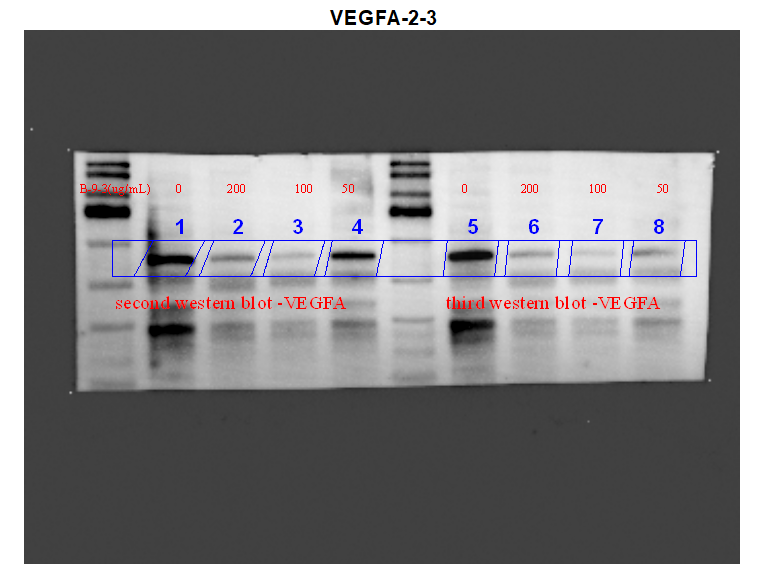


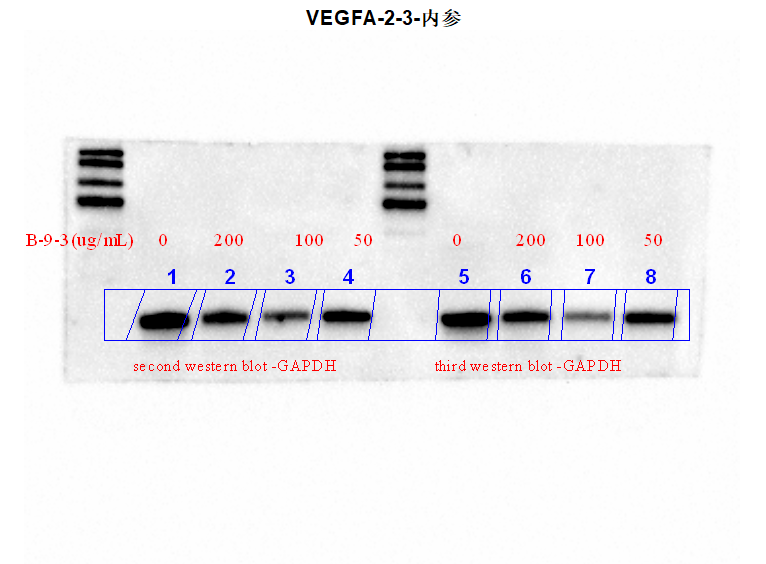


A549-PI3K


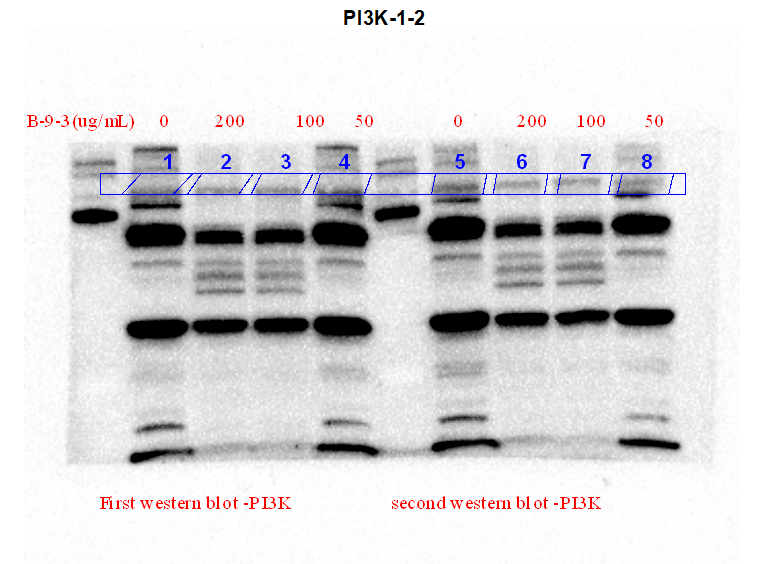

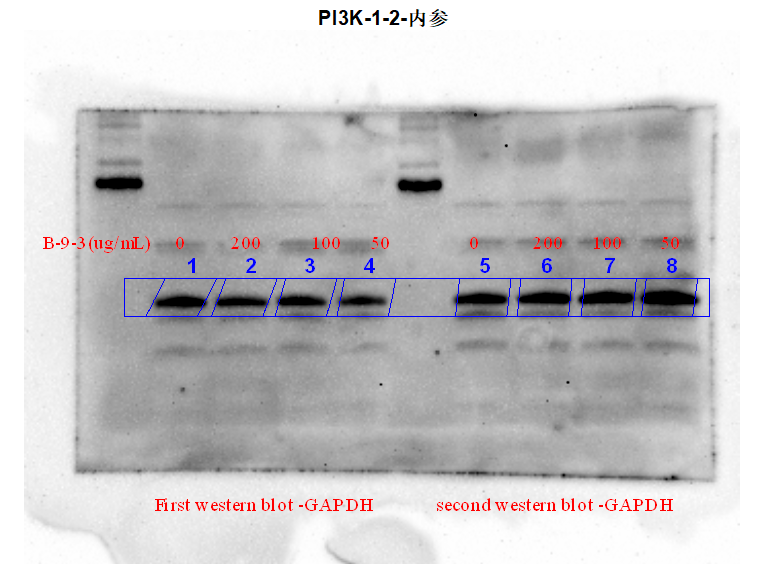


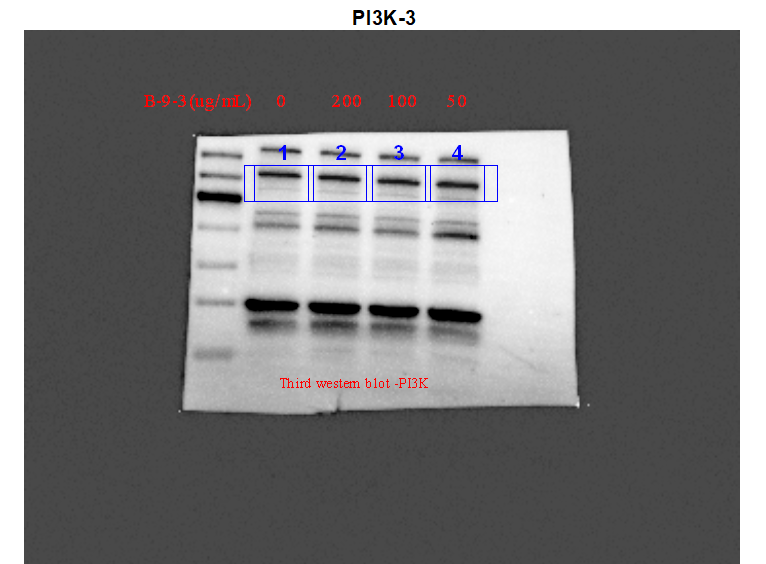

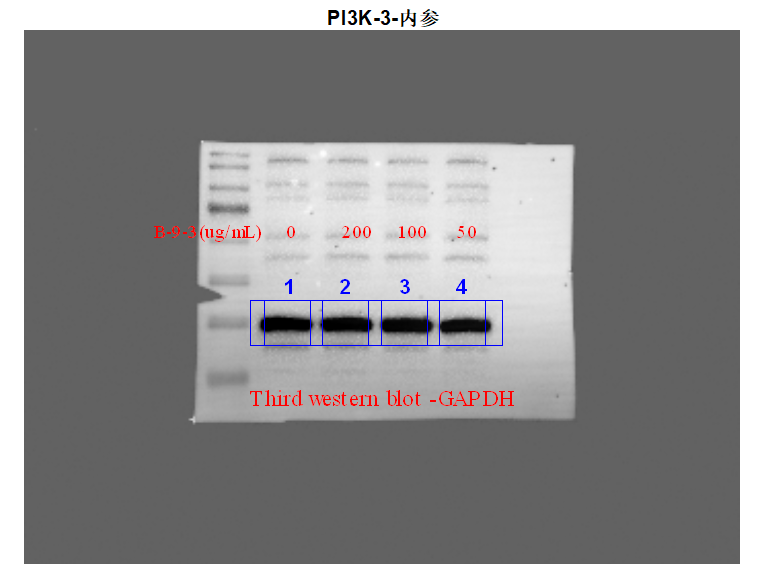


A549-p-PI3K


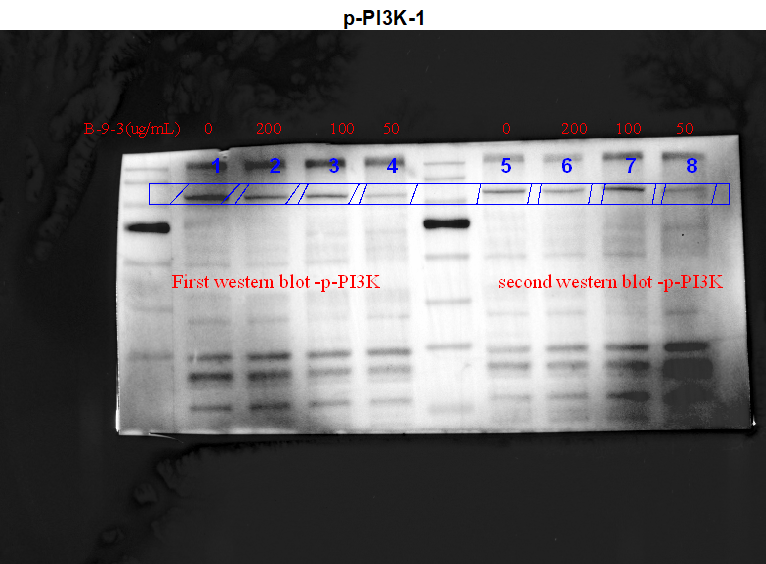

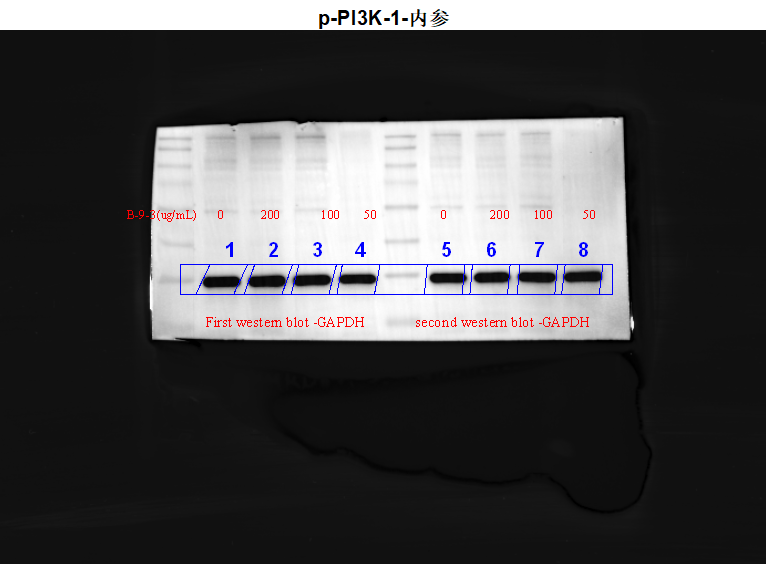


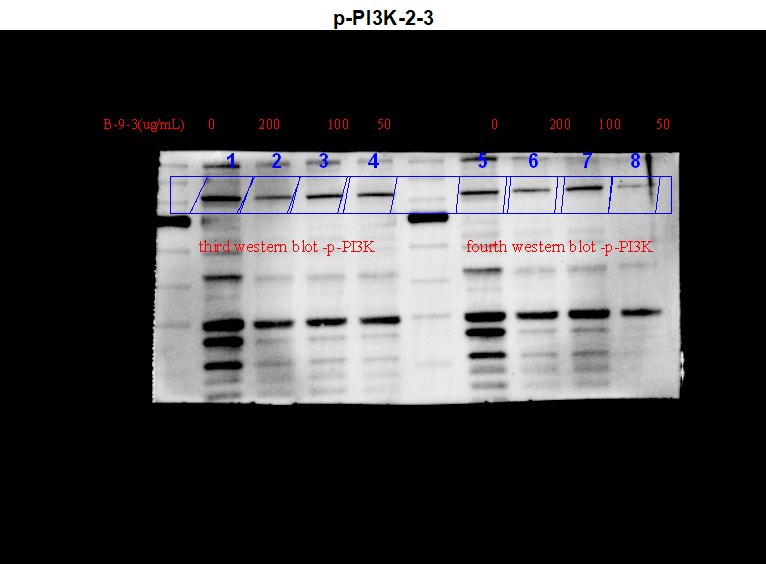

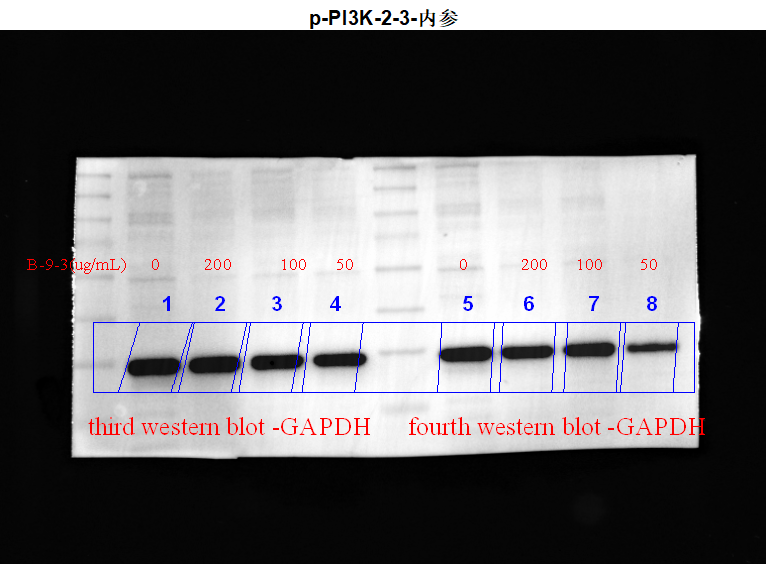


H226-PI3K


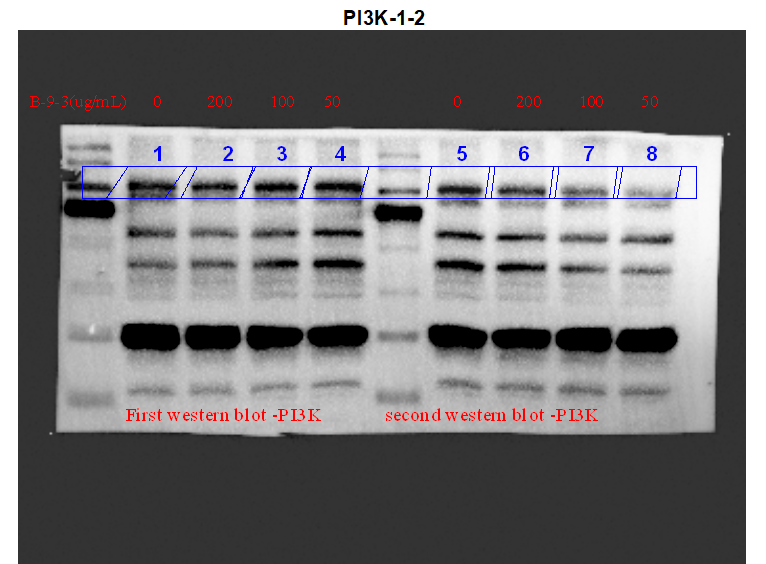

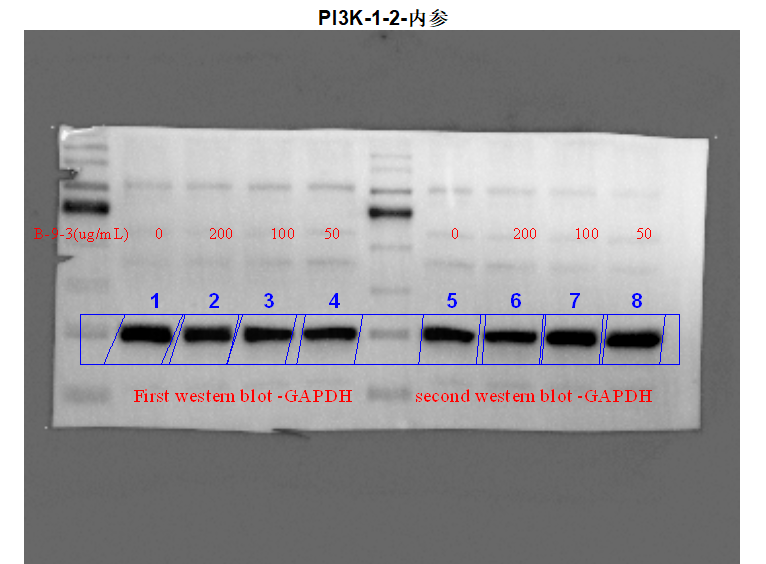


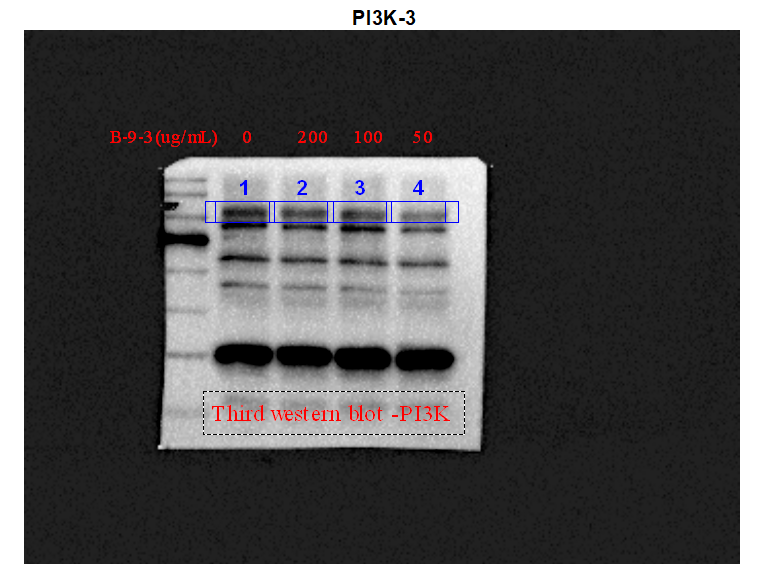

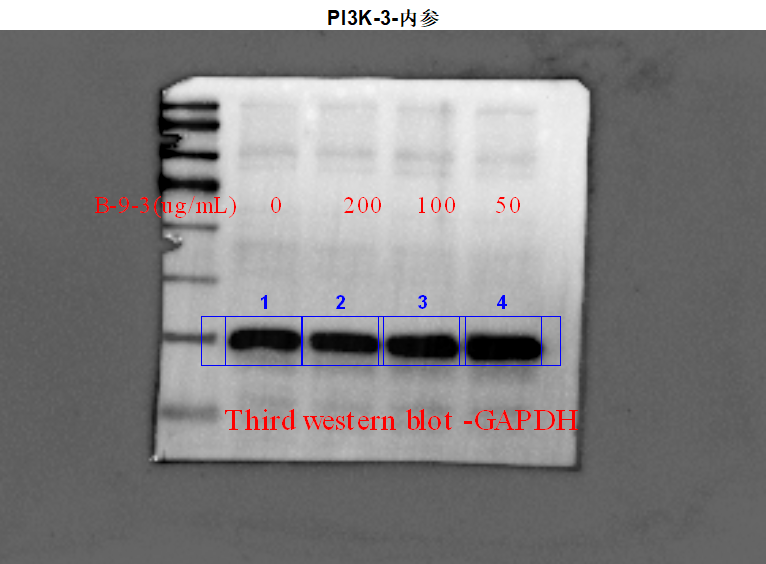


H226-p-PI3K


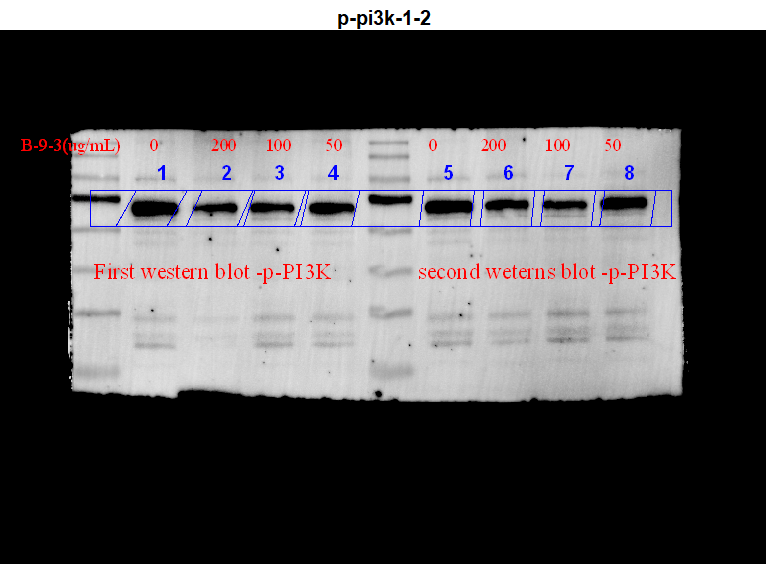

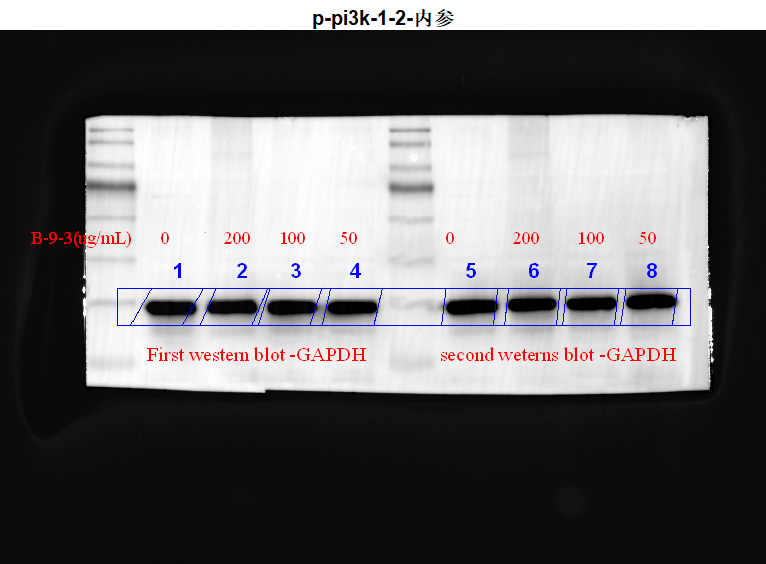


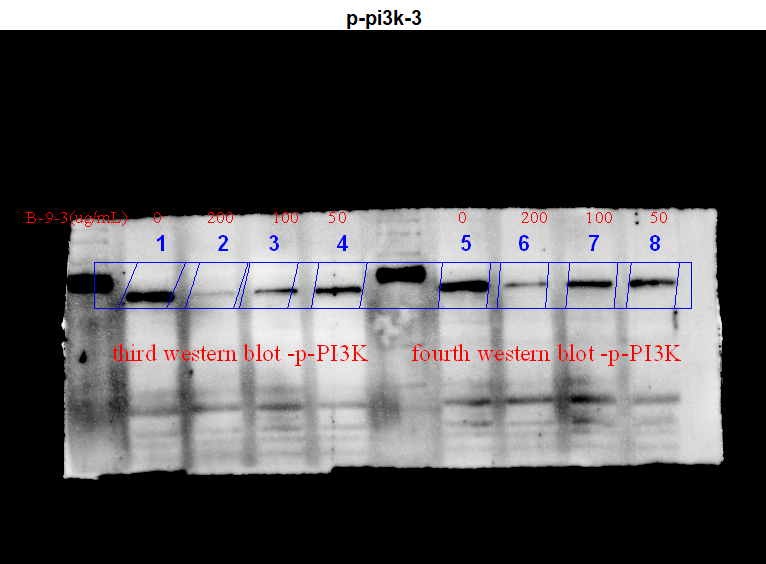

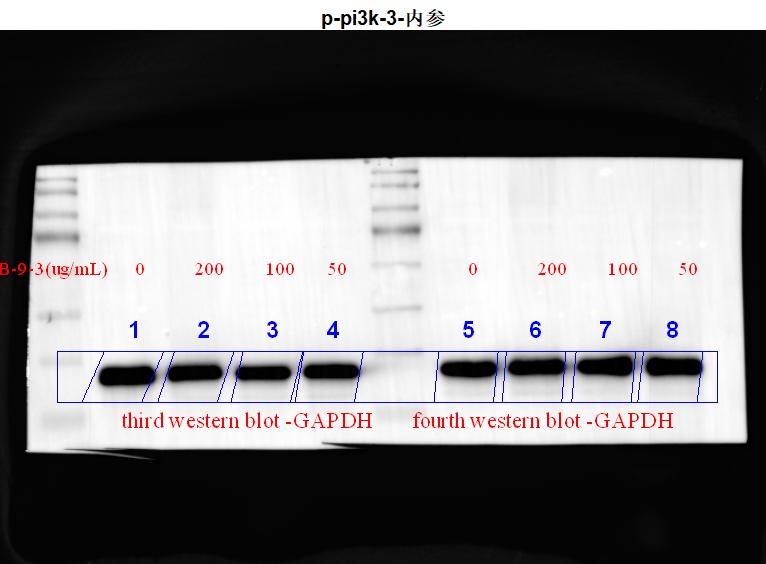


H460-PI3K

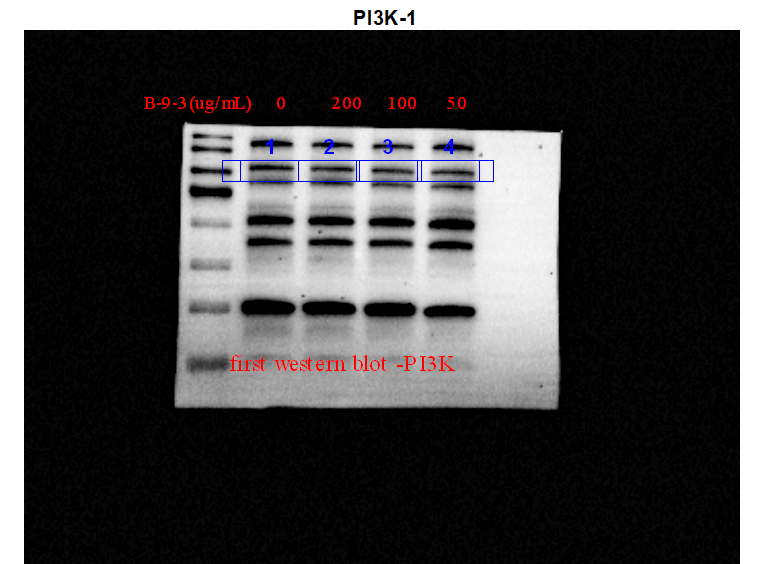

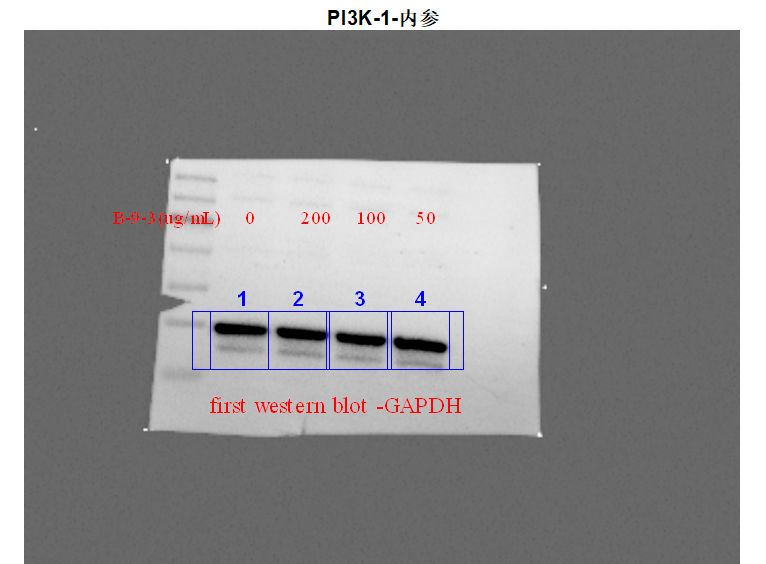


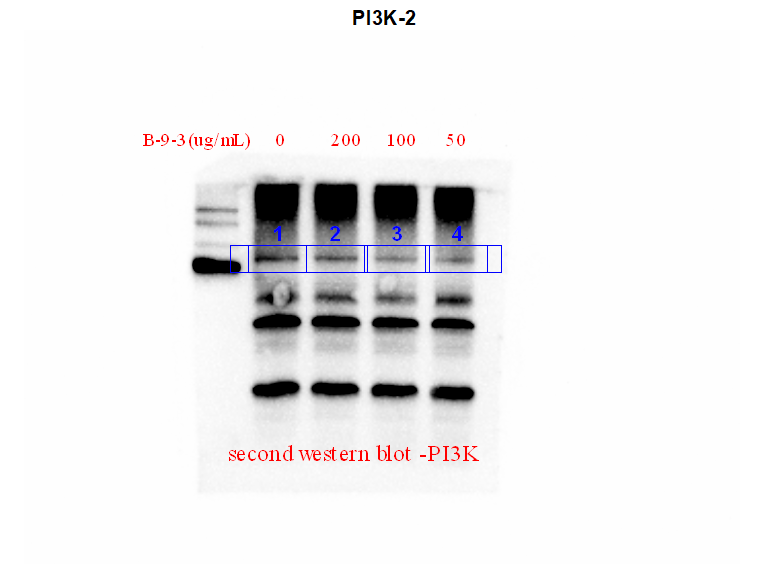

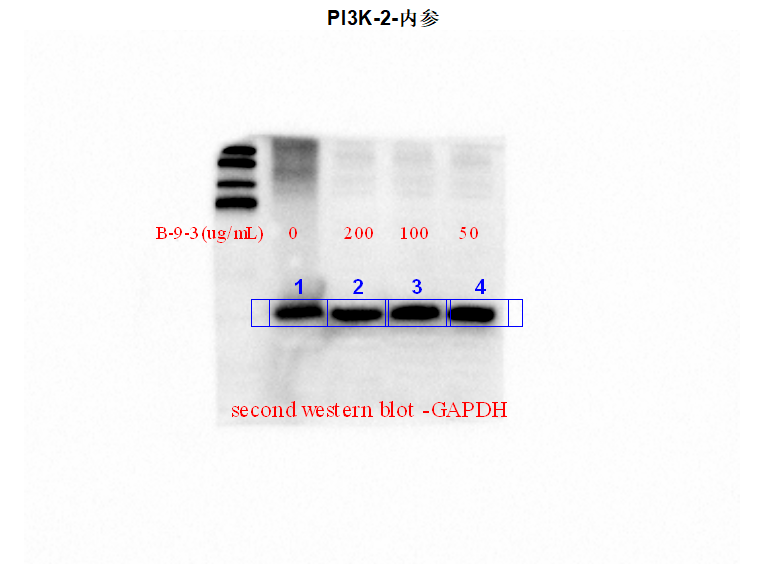


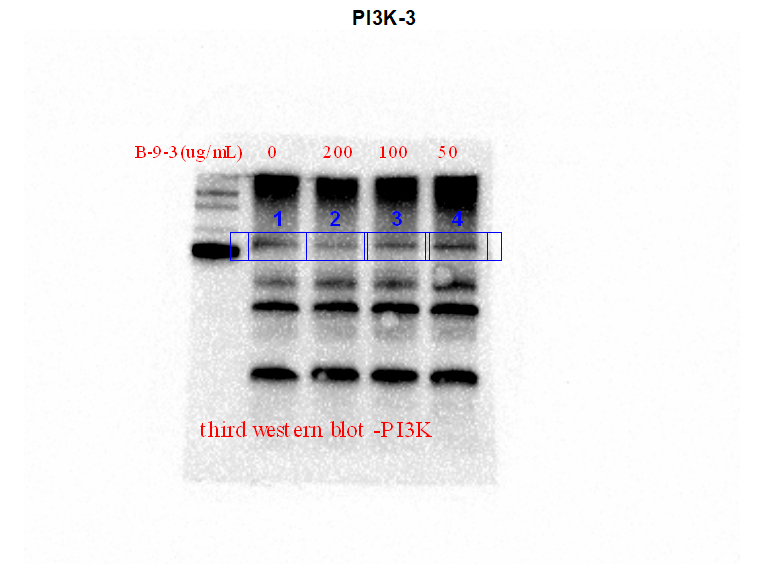

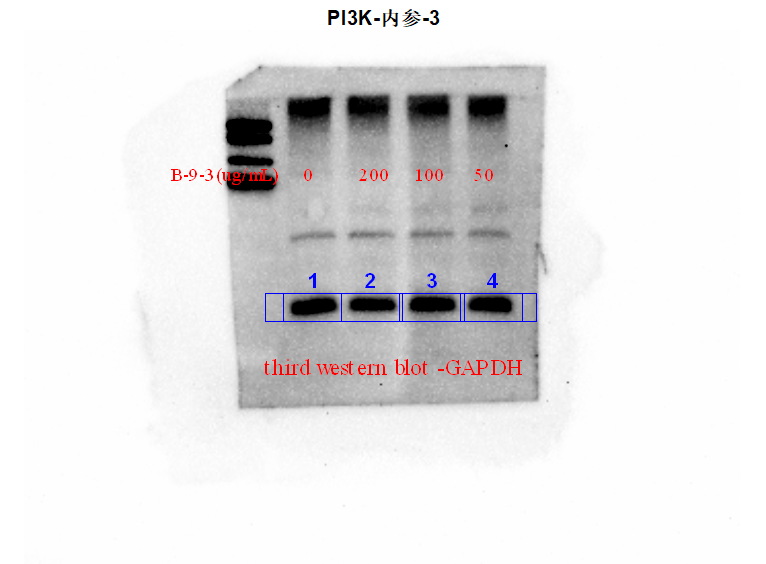


H460-p-PI3K


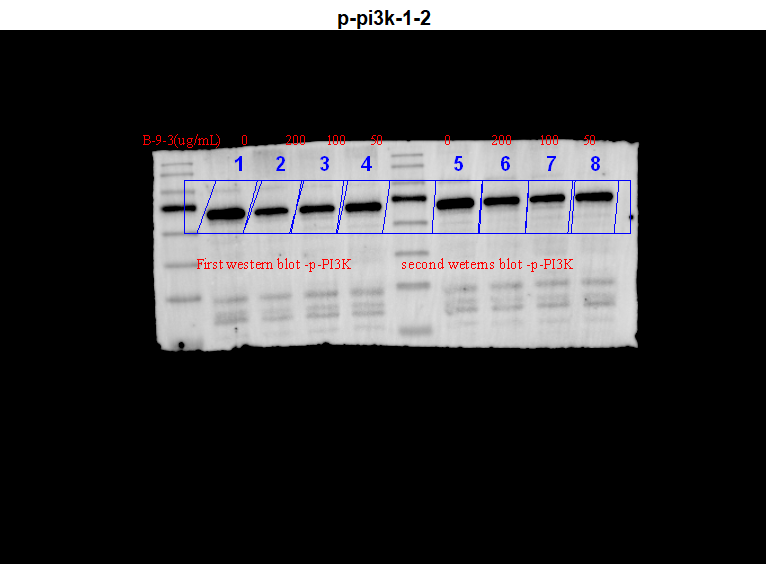

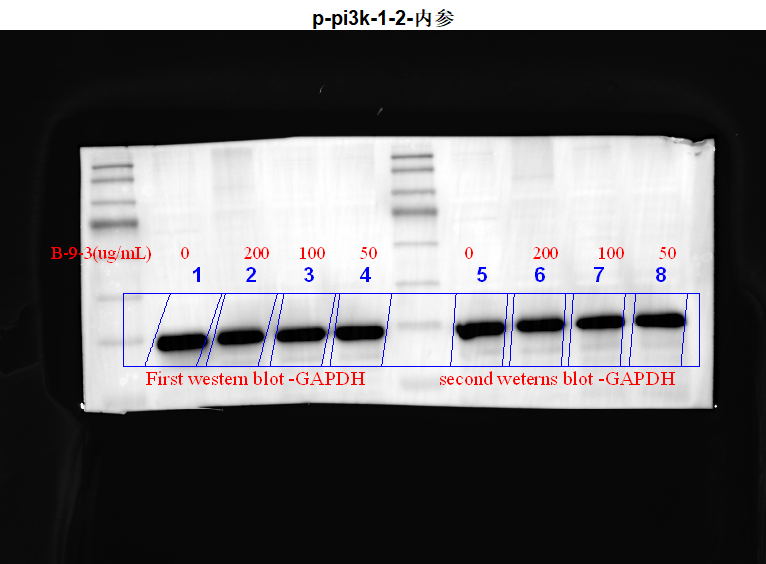


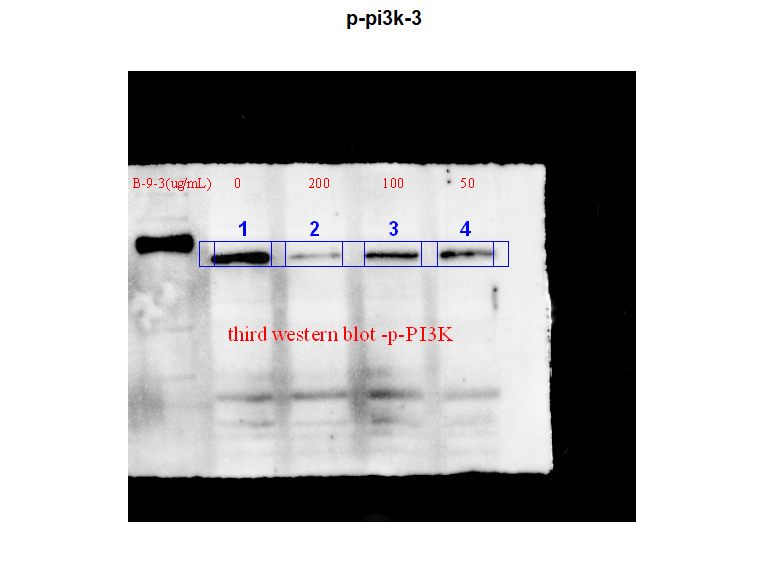

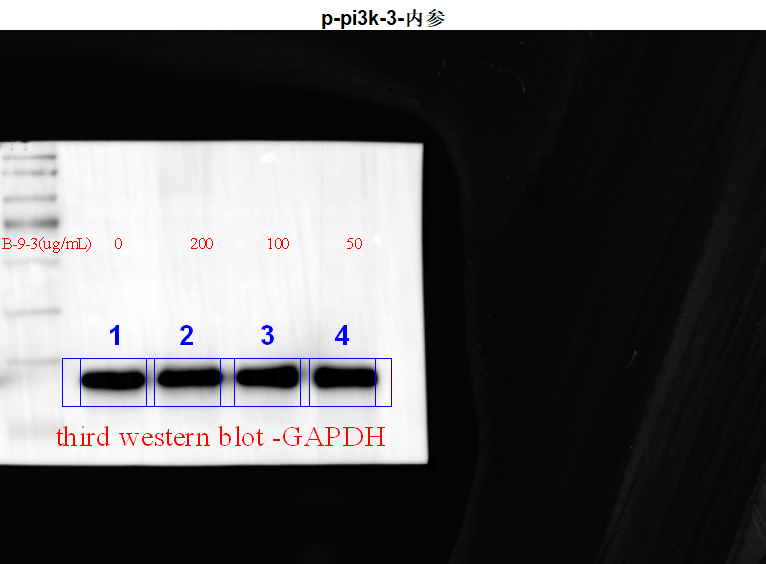


A549-AKT


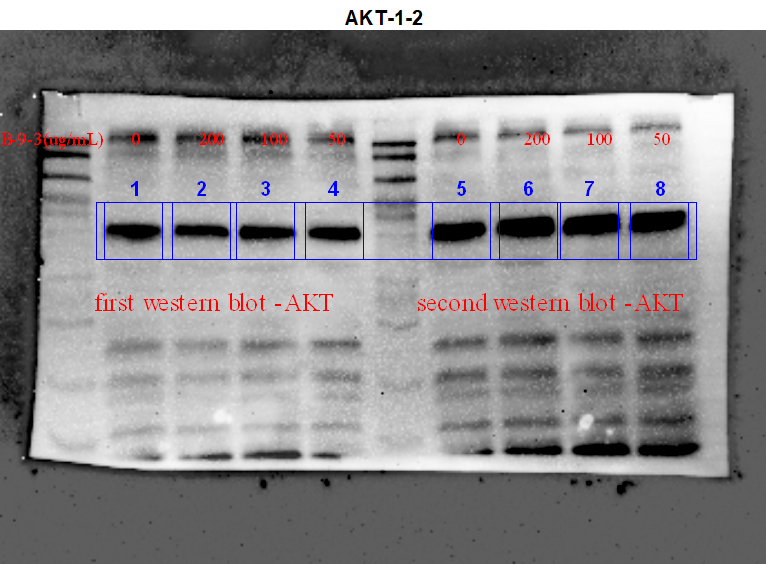

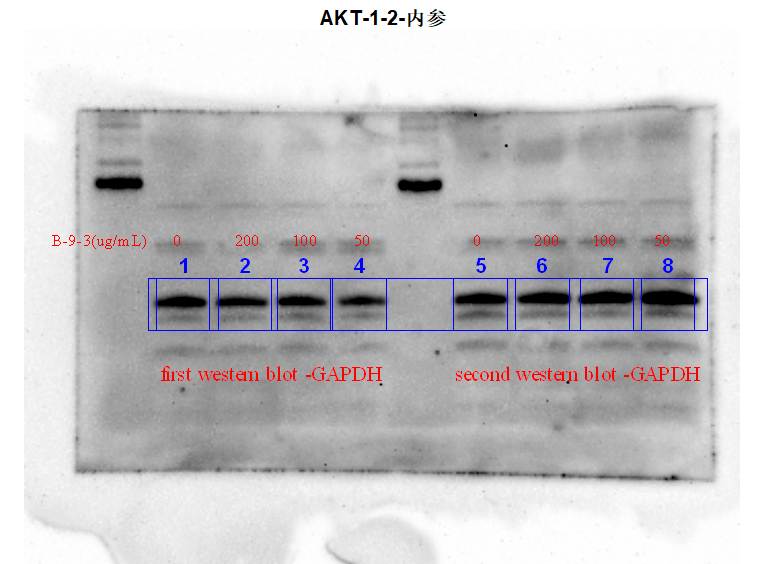


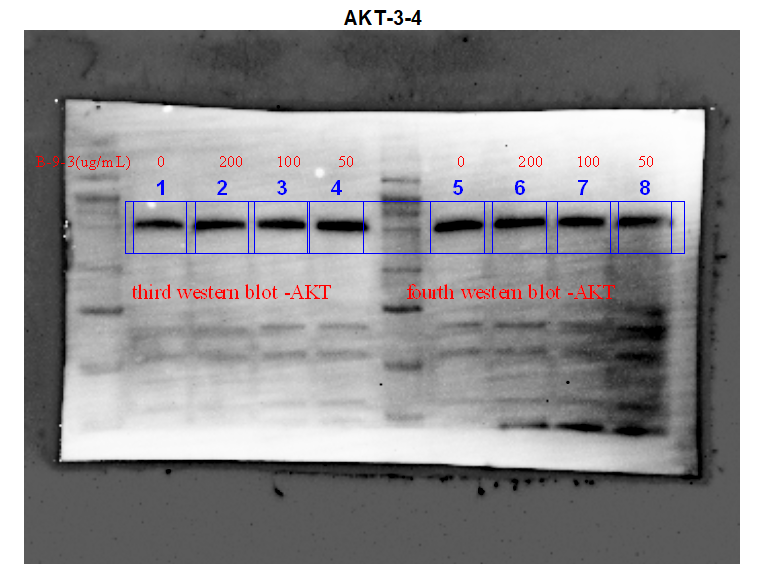

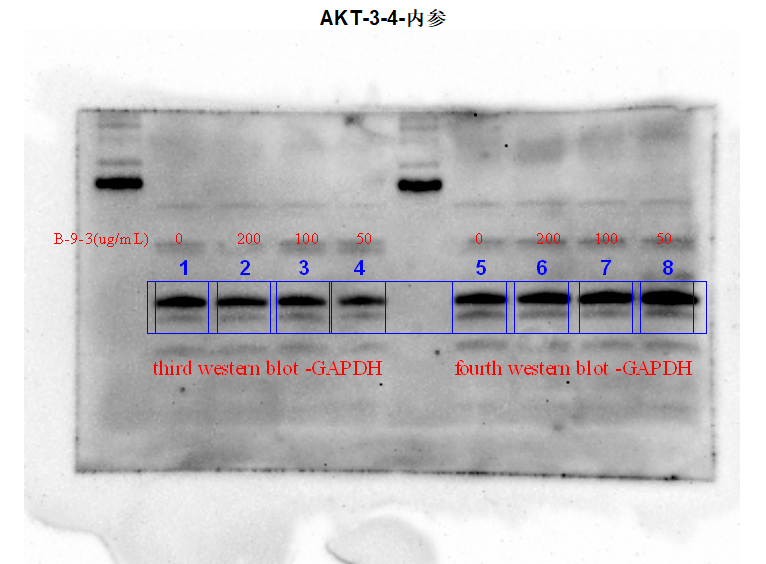


A549-p-AKT


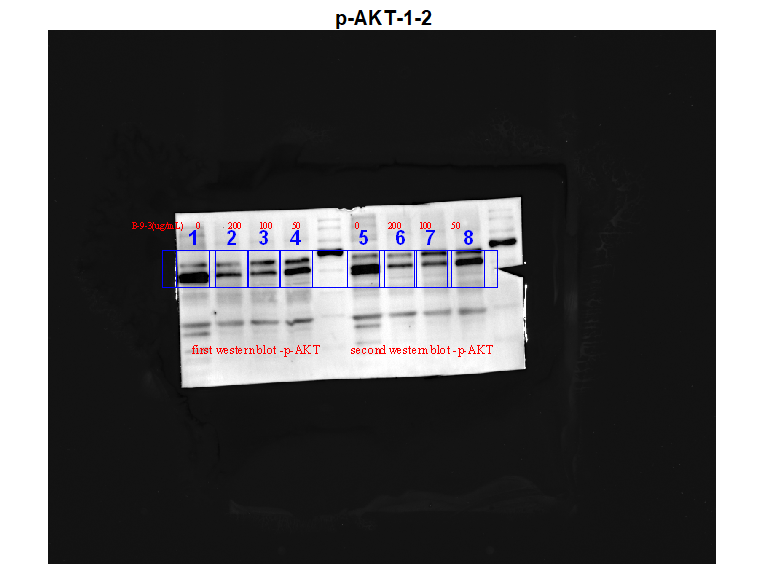

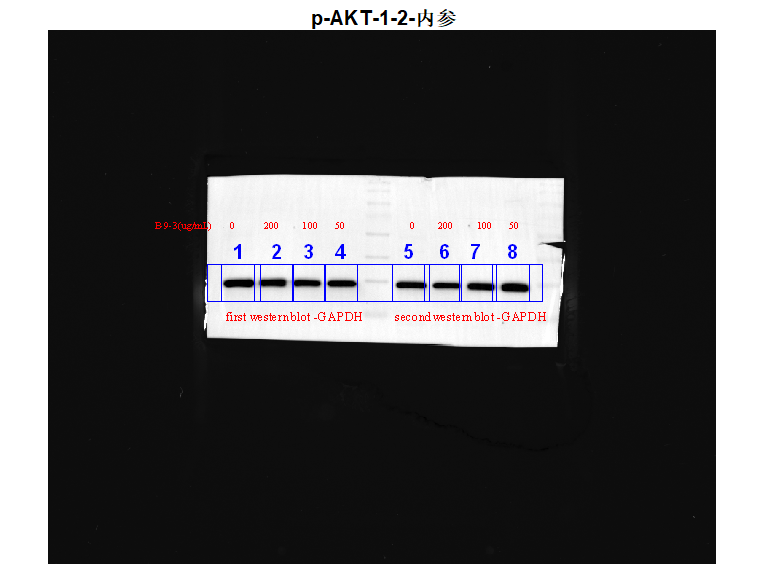


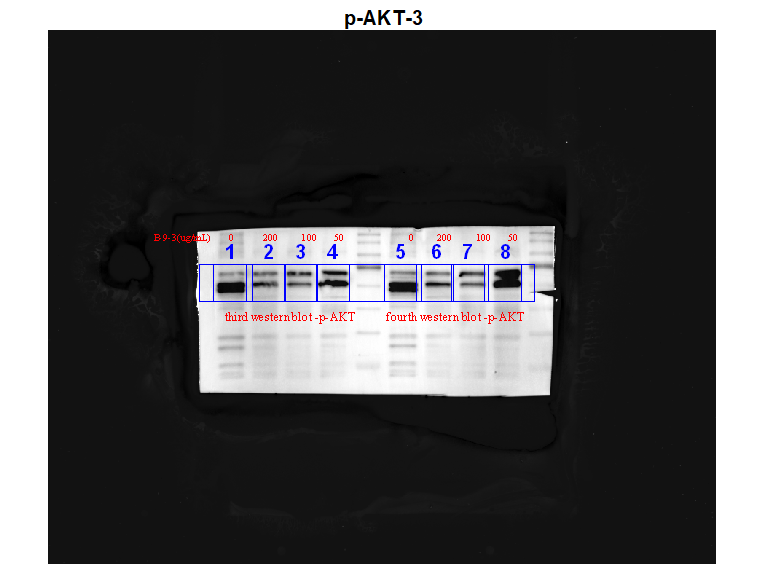

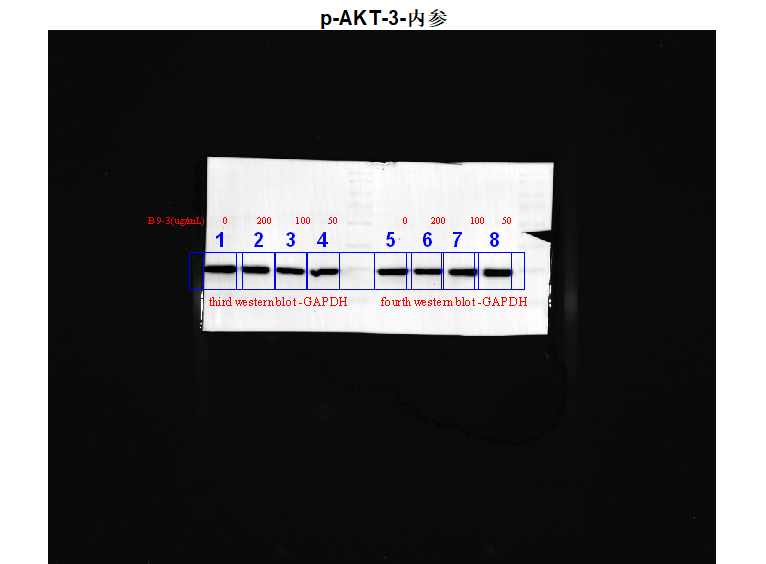


H226-AKT


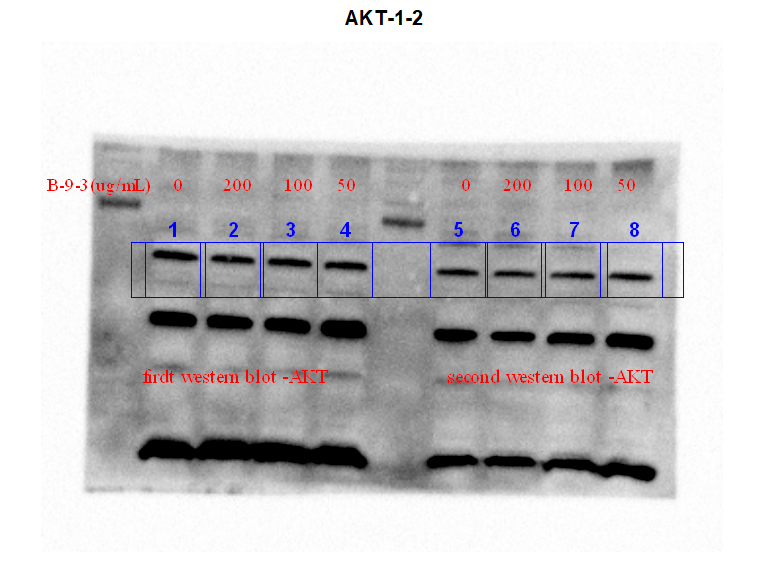

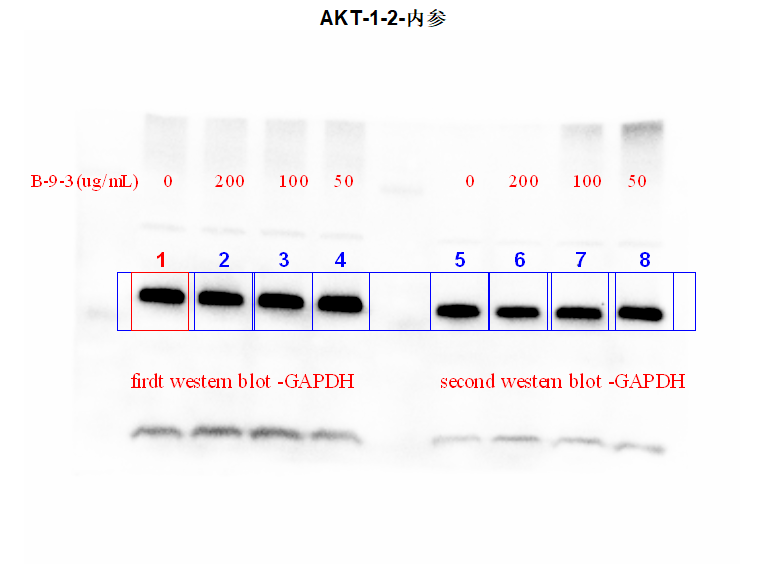


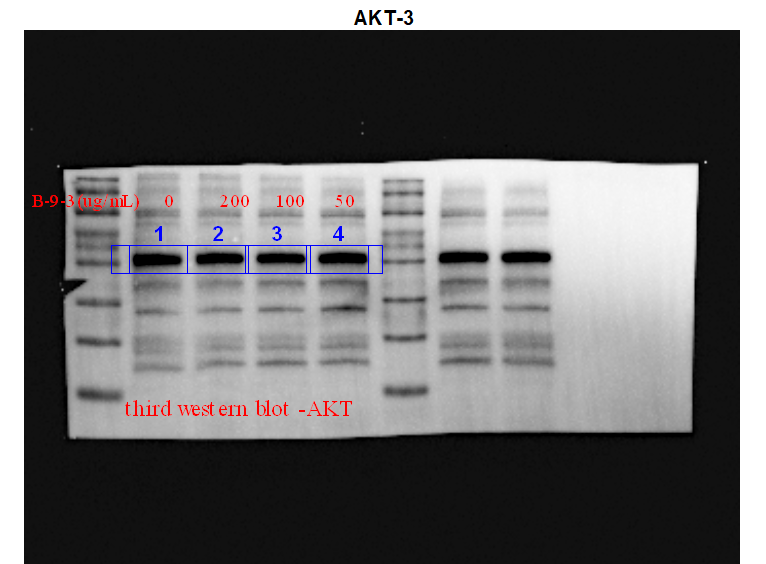

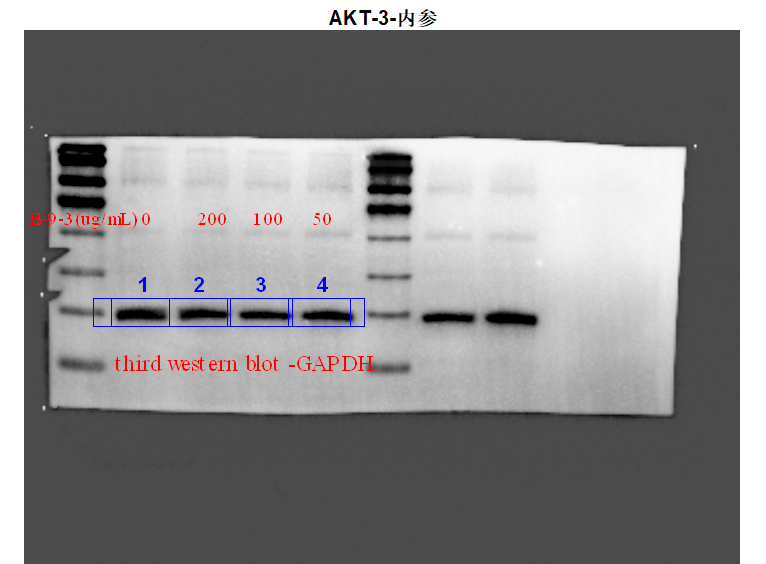


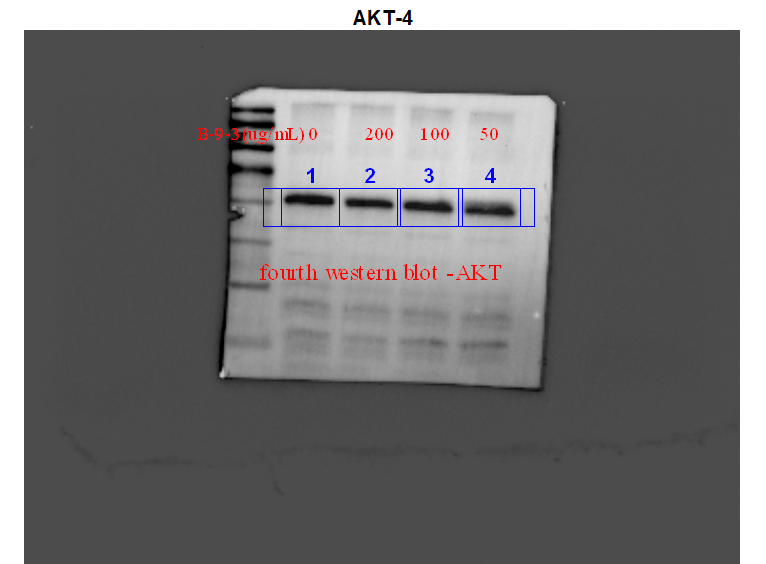

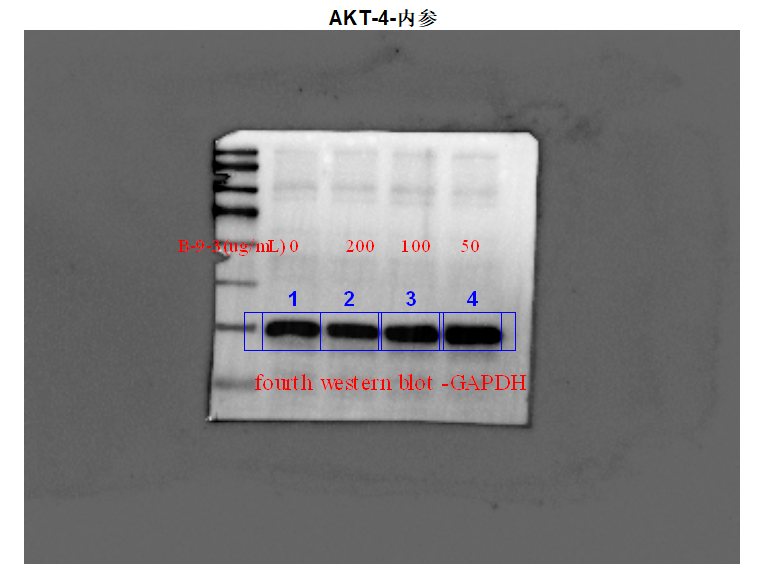


H226-p-AKT


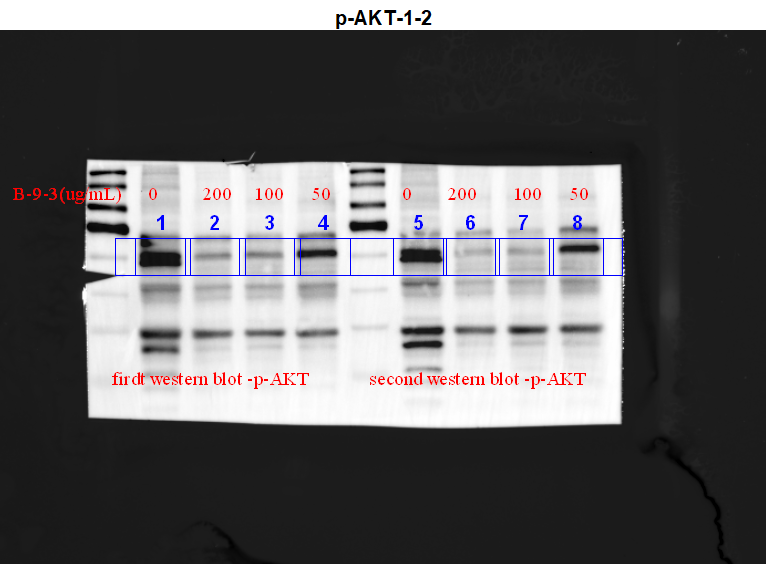

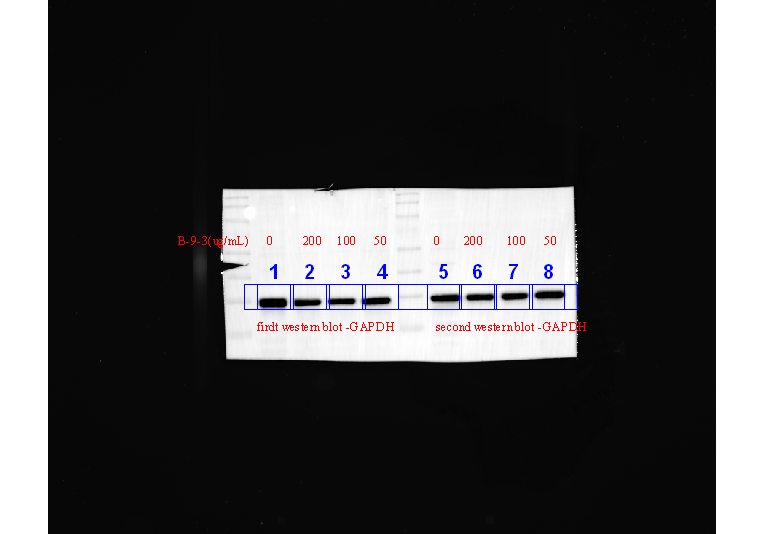


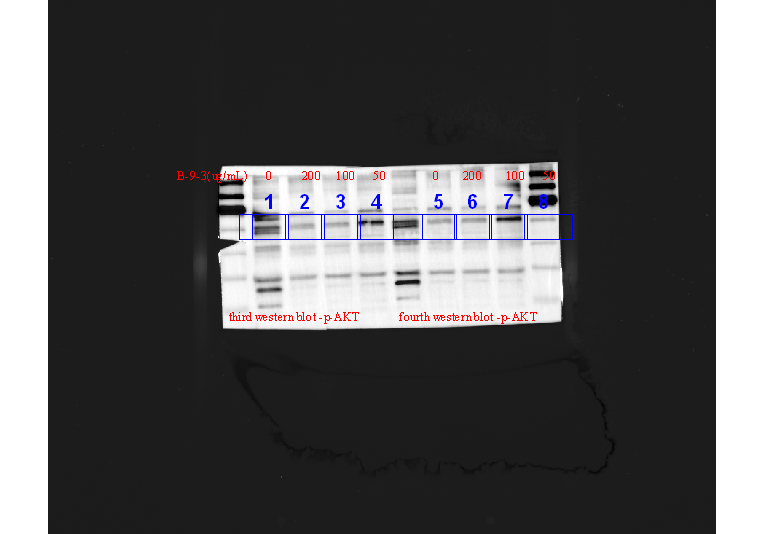

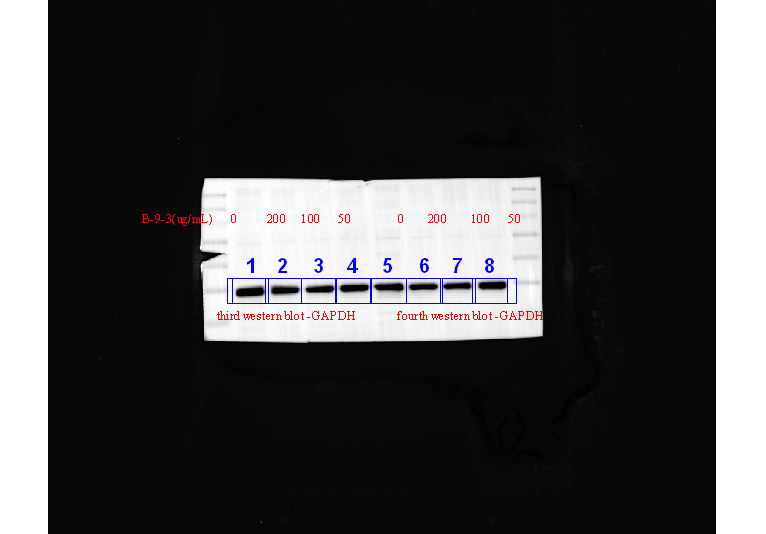


H460-AKT


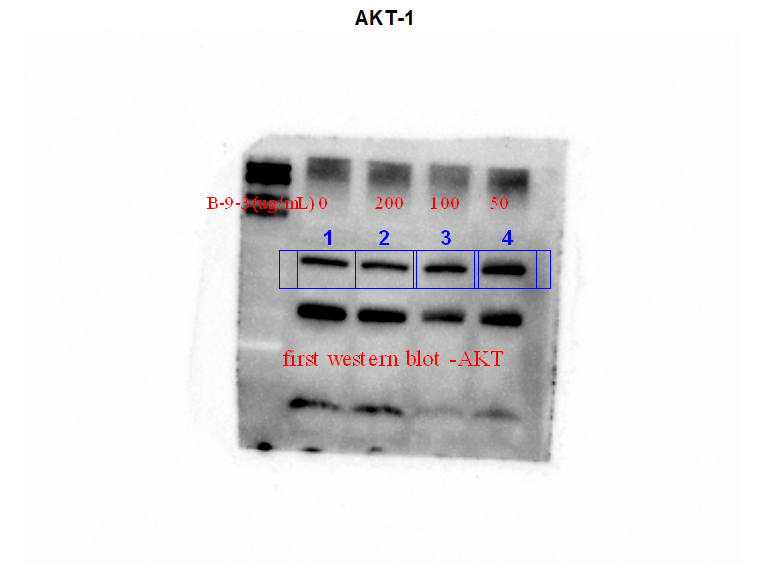

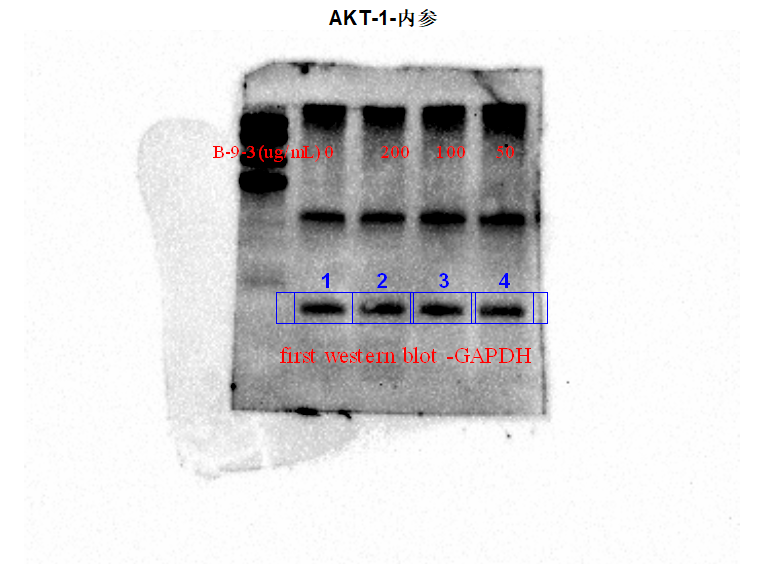


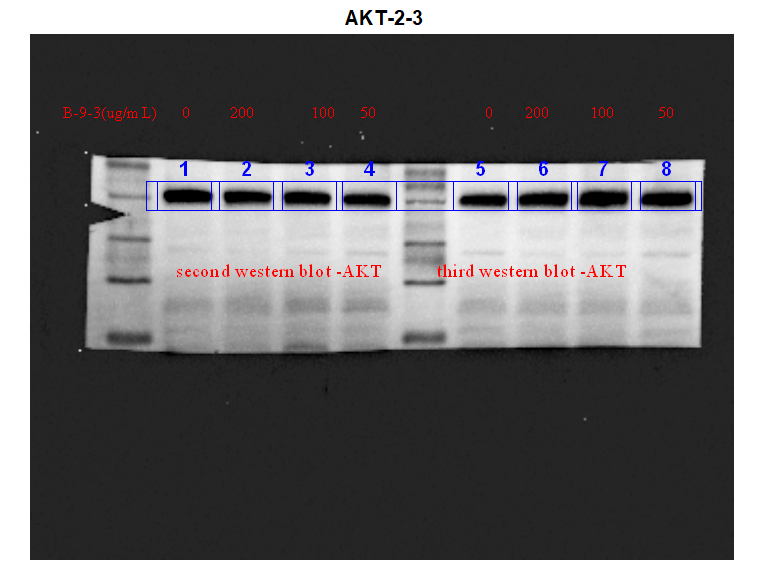


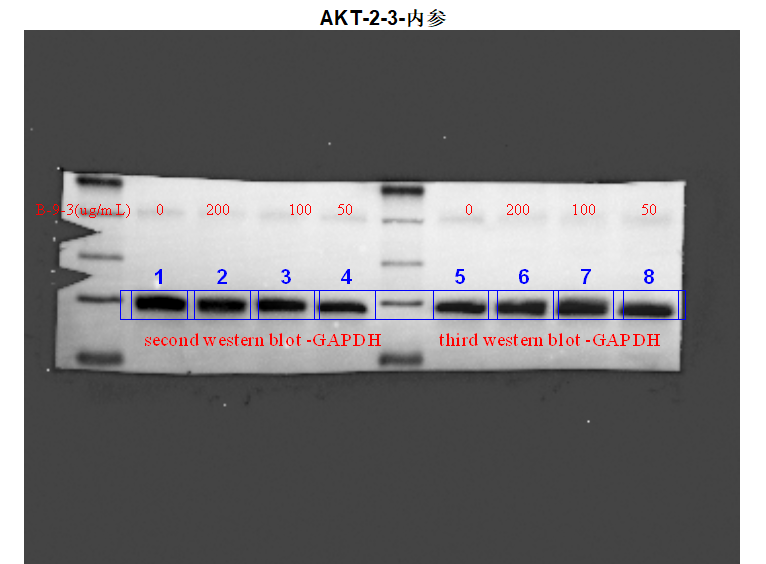


H460-p-AKT


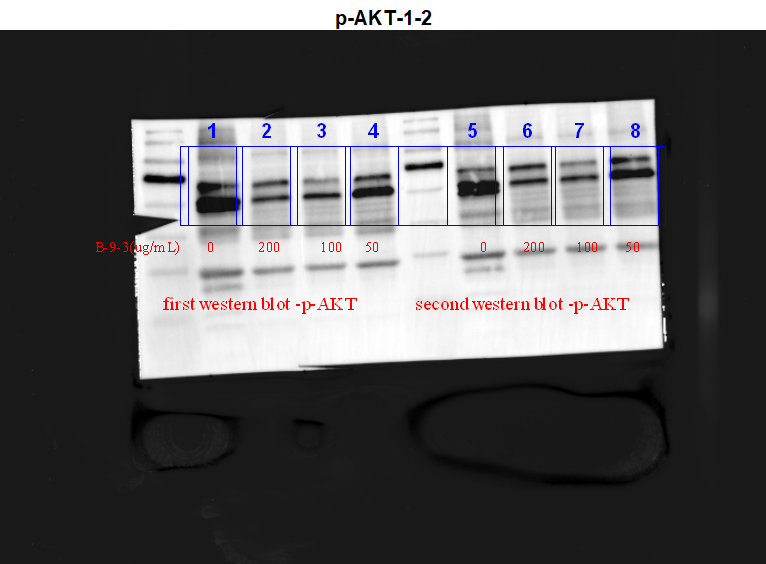


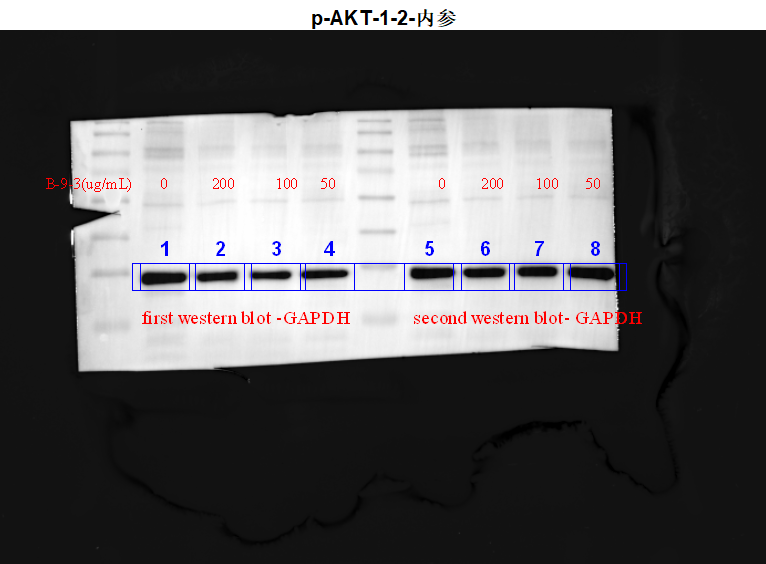


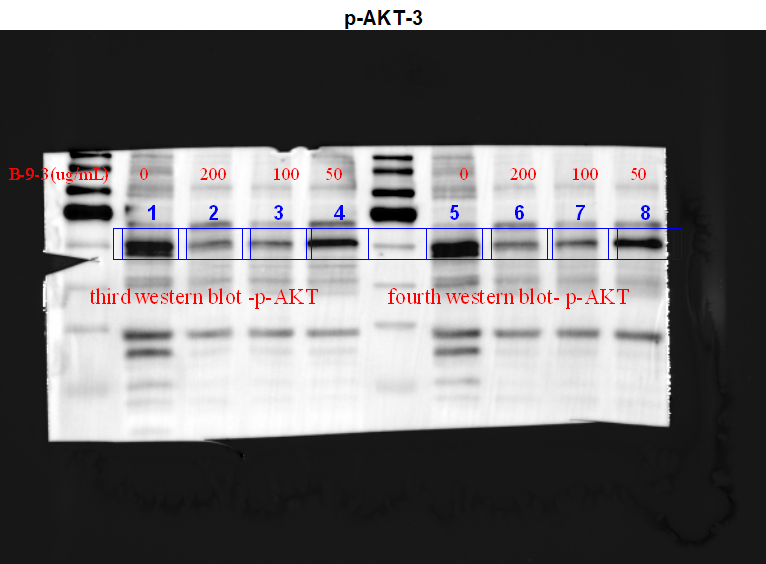


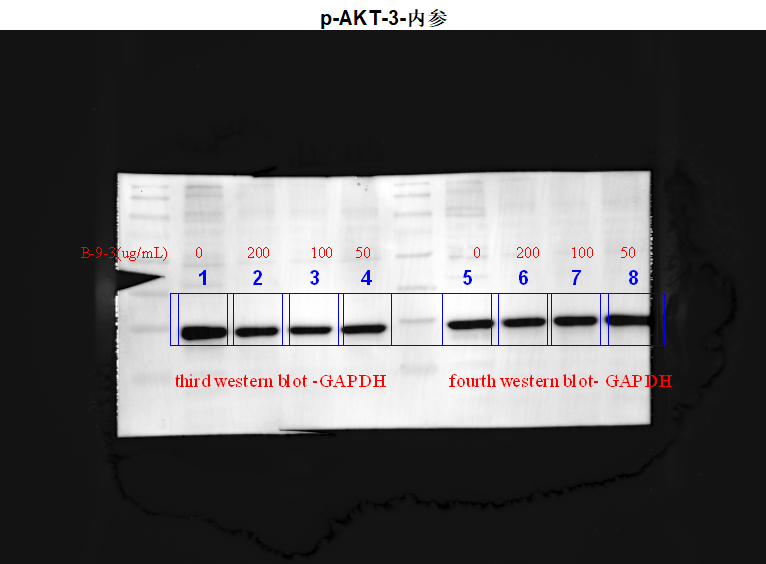


A549-Bax


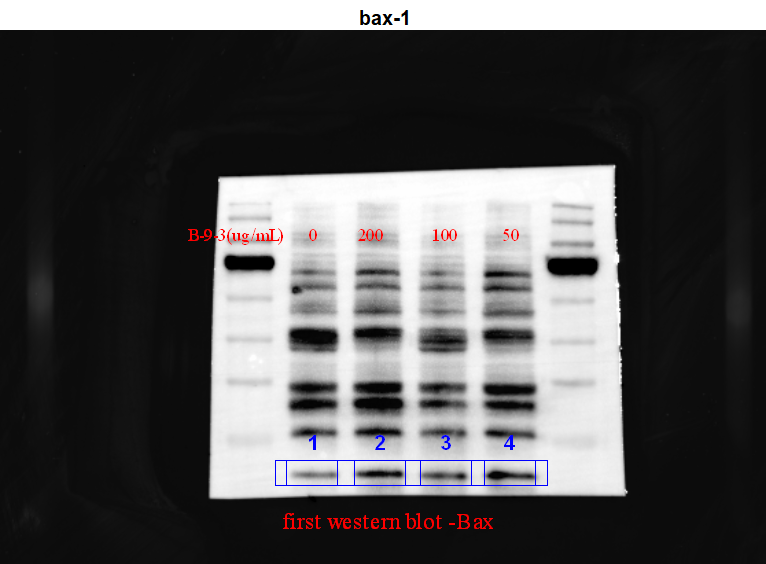

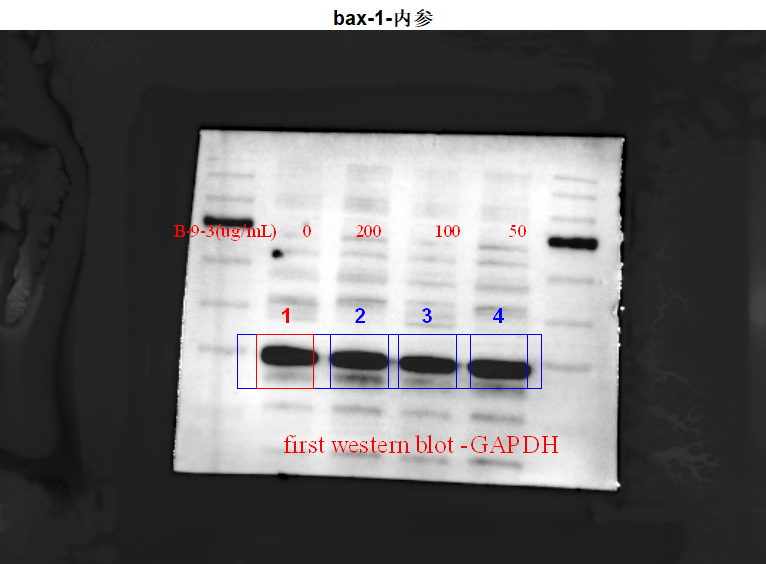


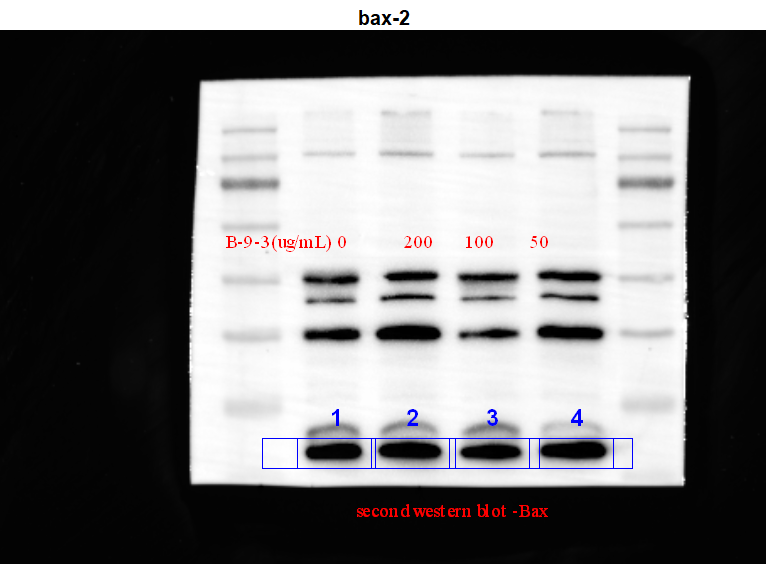

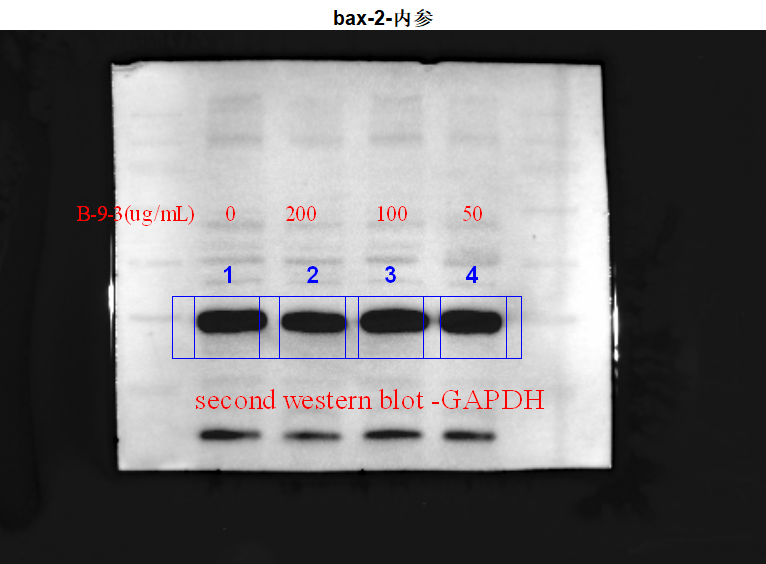


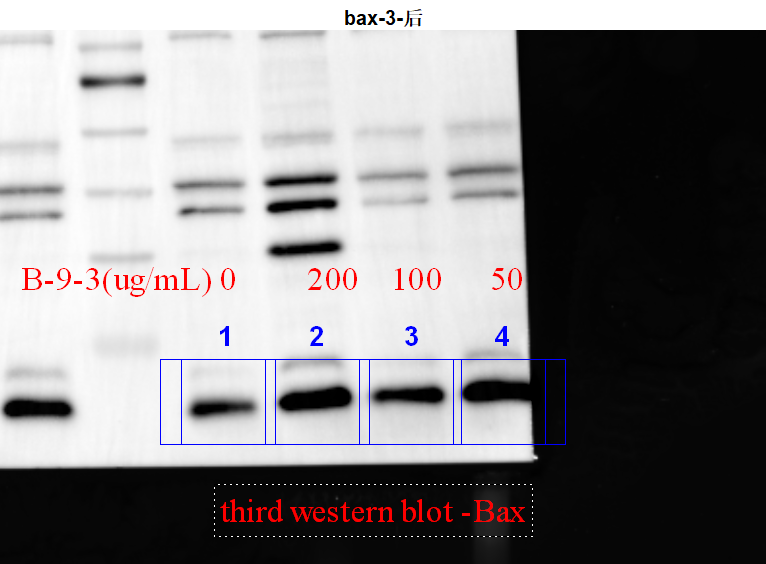

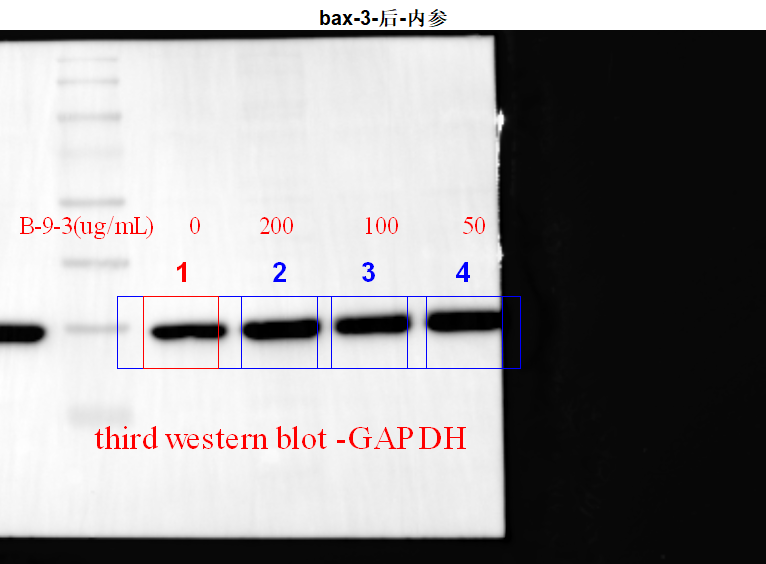


H226-Bax


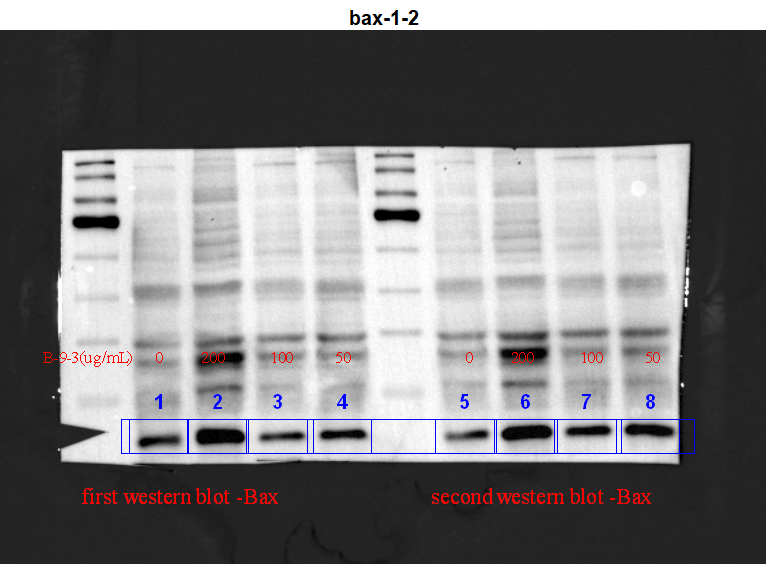

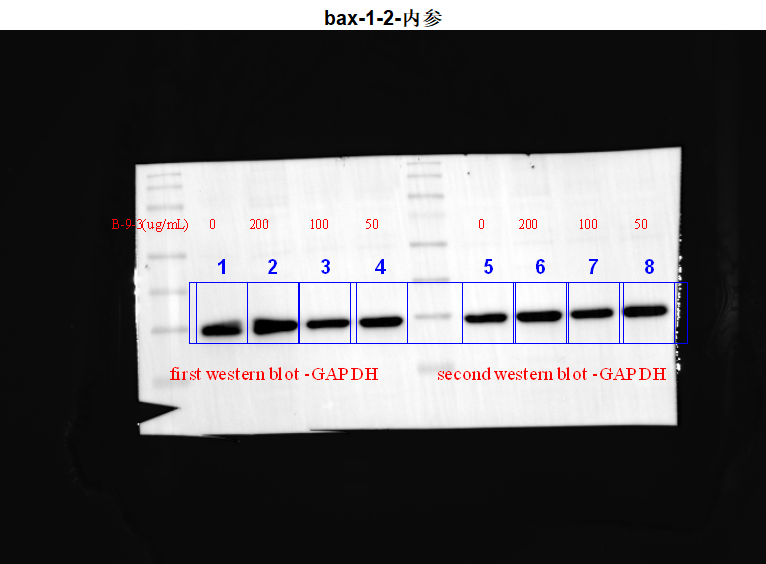


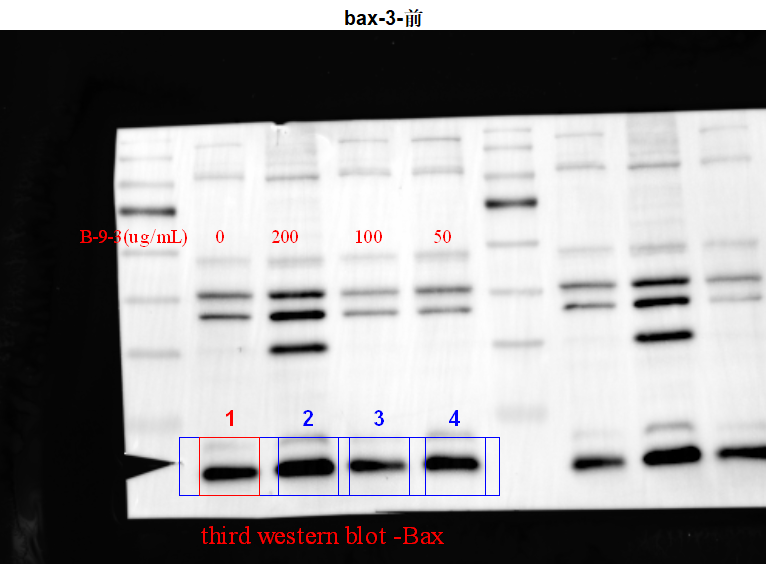

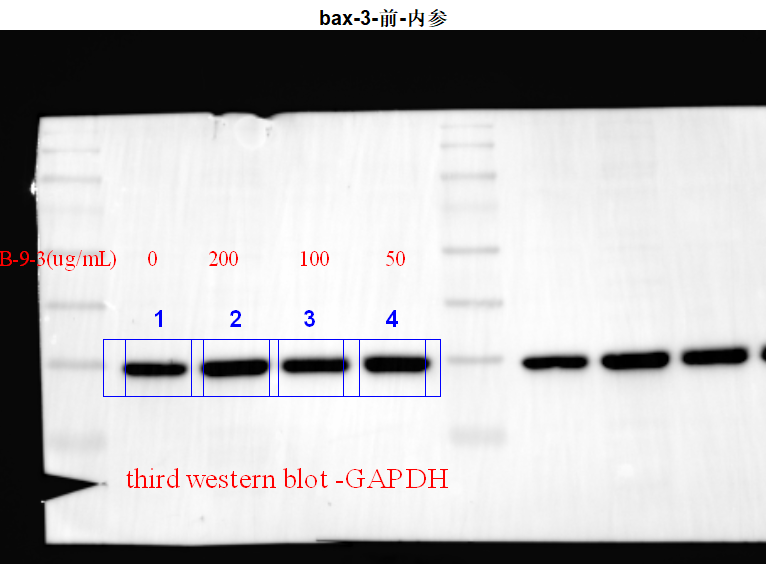


H460-Bax


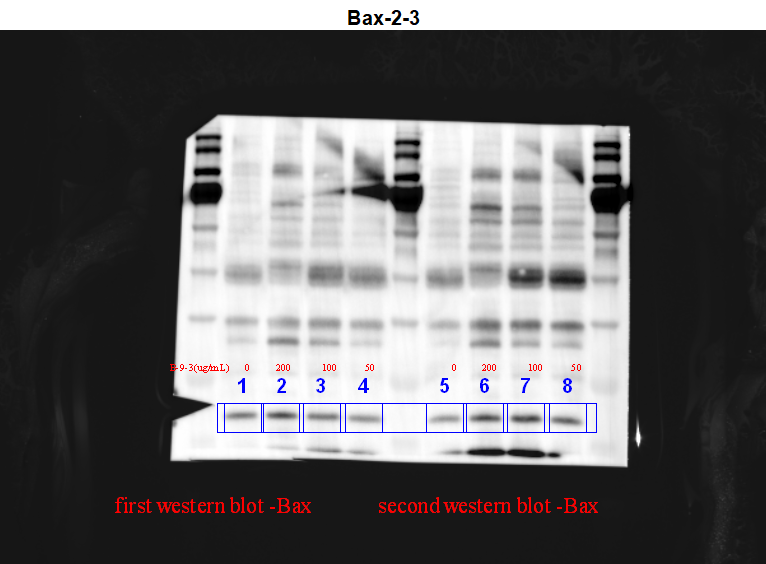

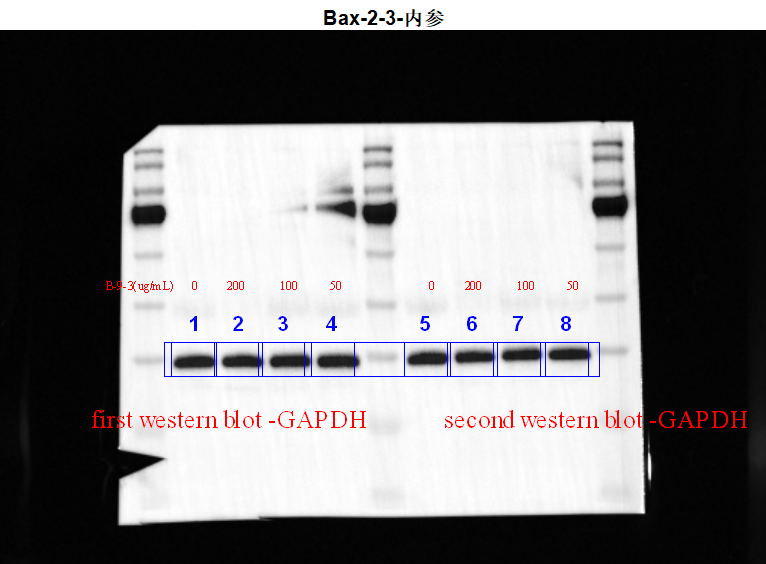


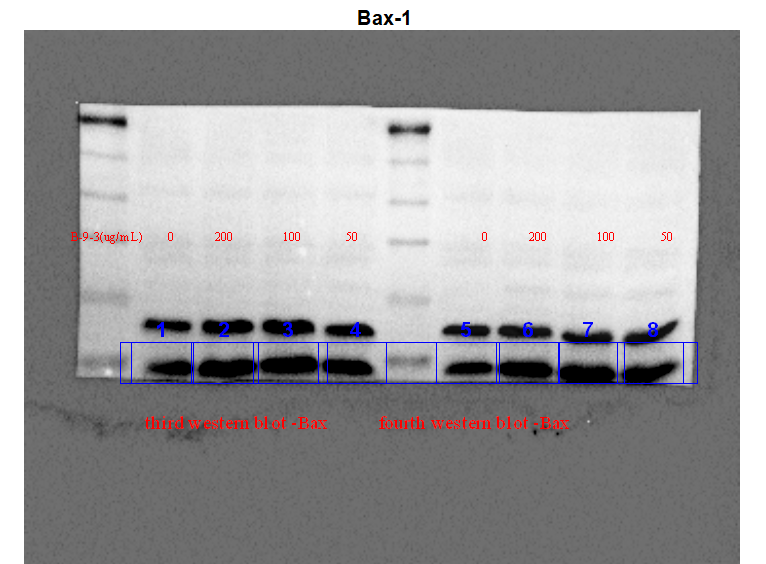

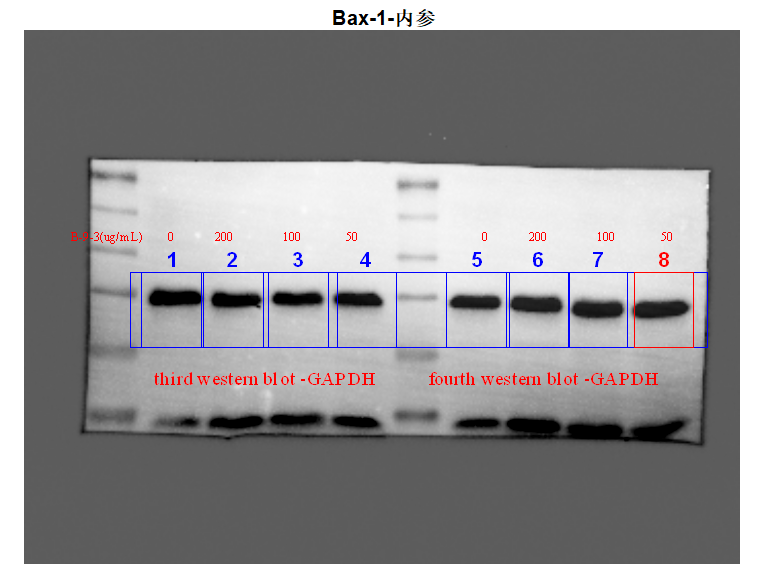


A549-Bcl-2


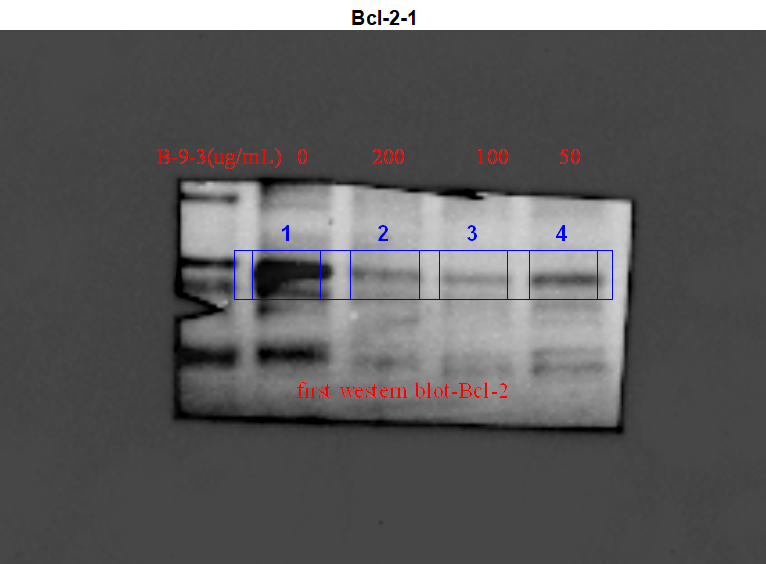

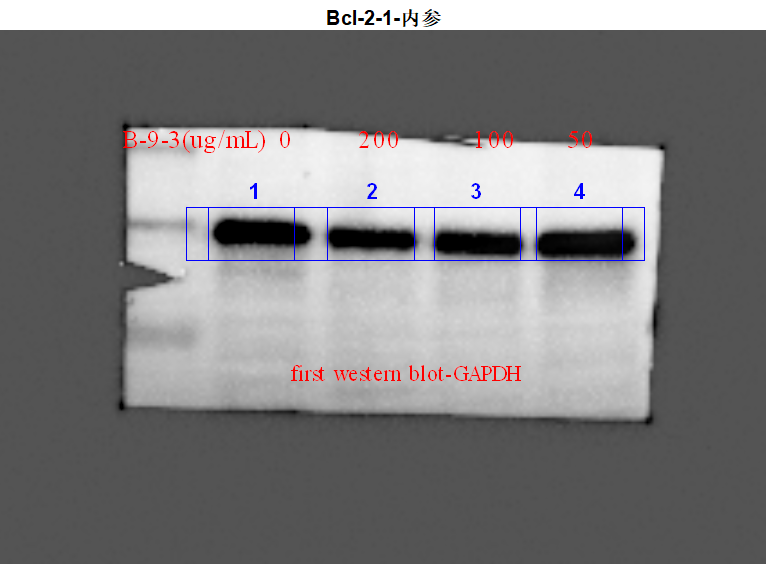


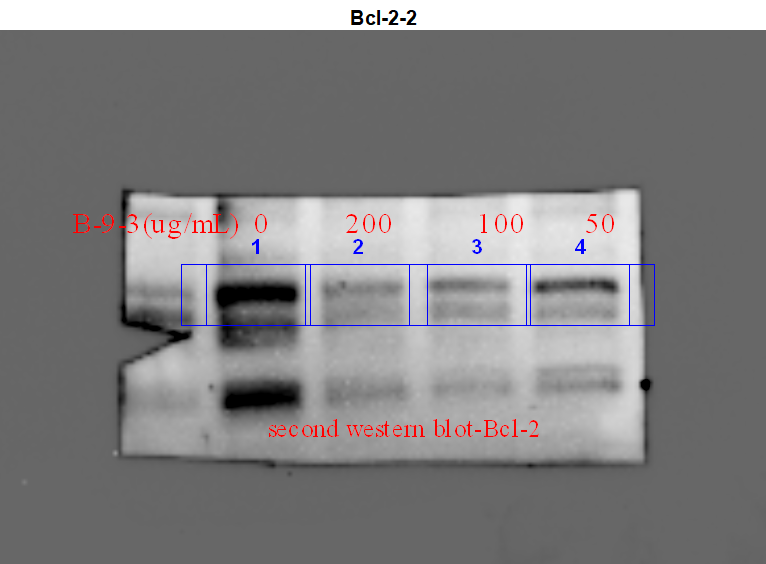

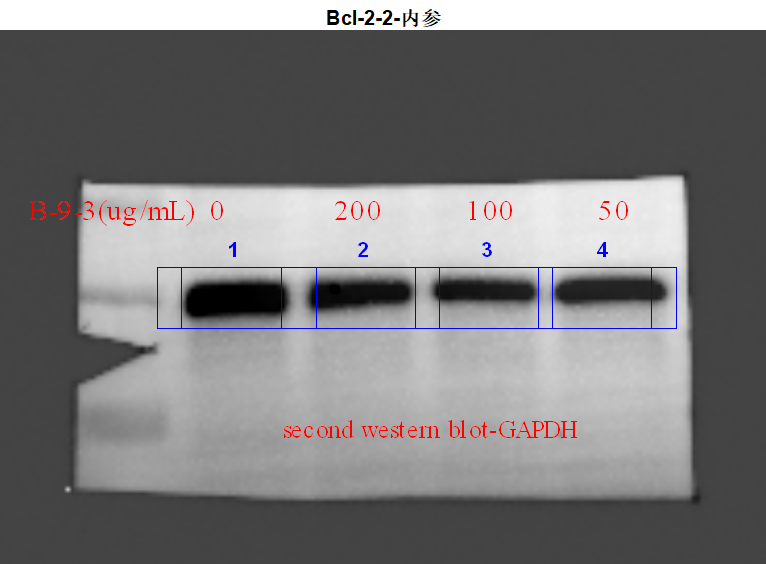


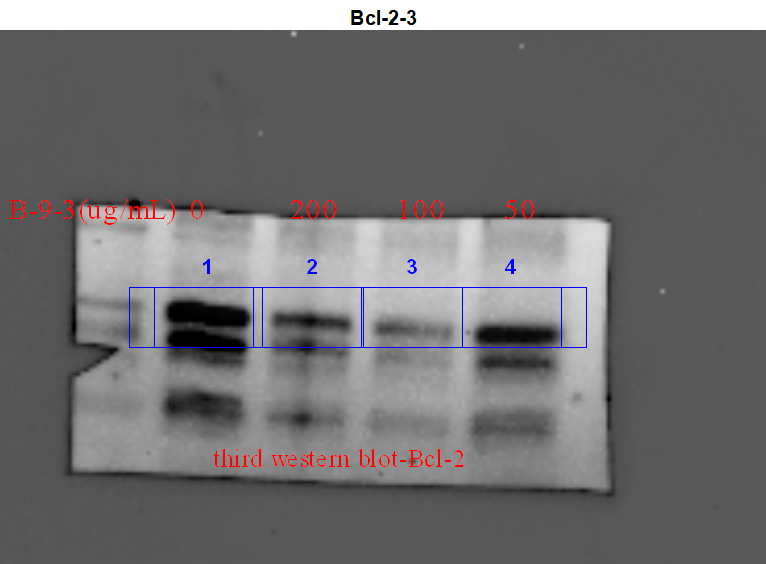

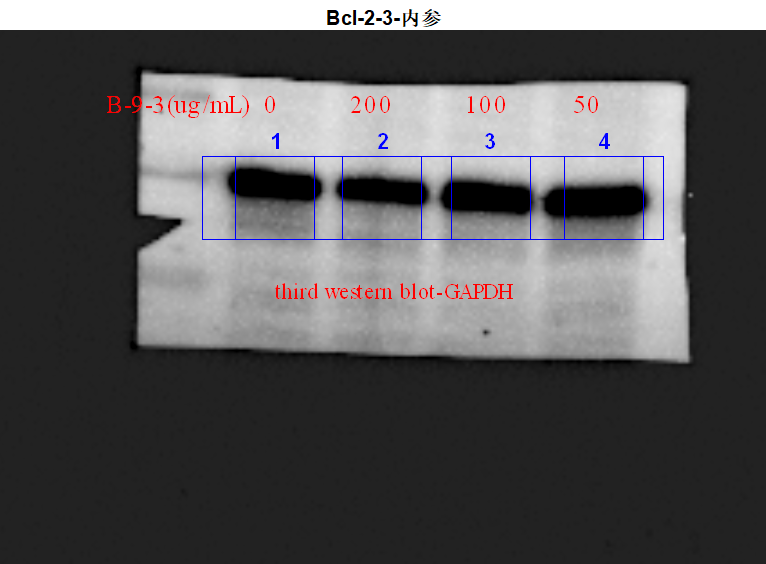


H226-Bcl-2


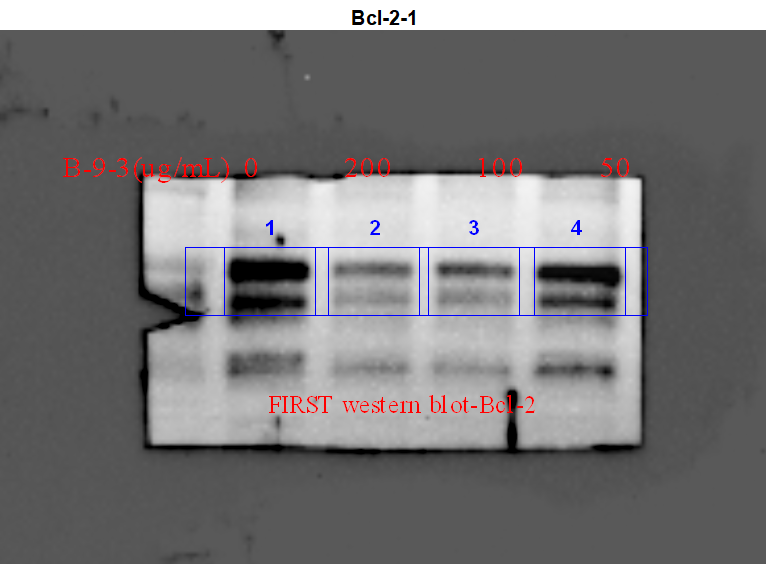

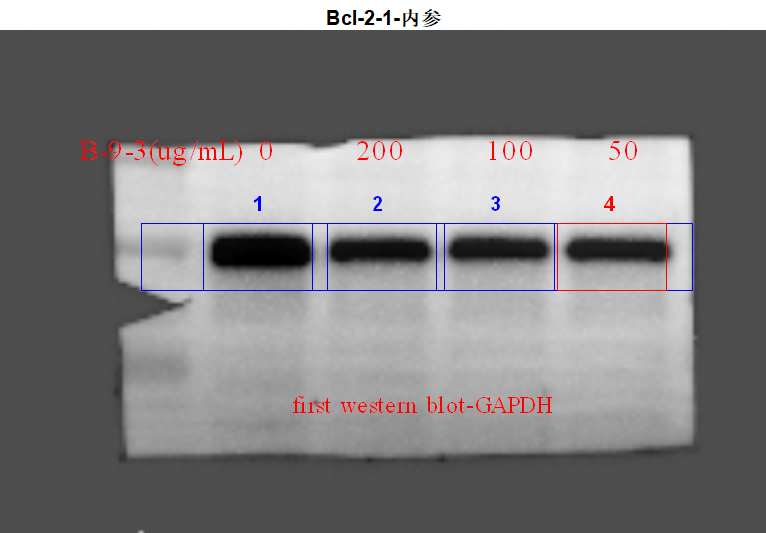


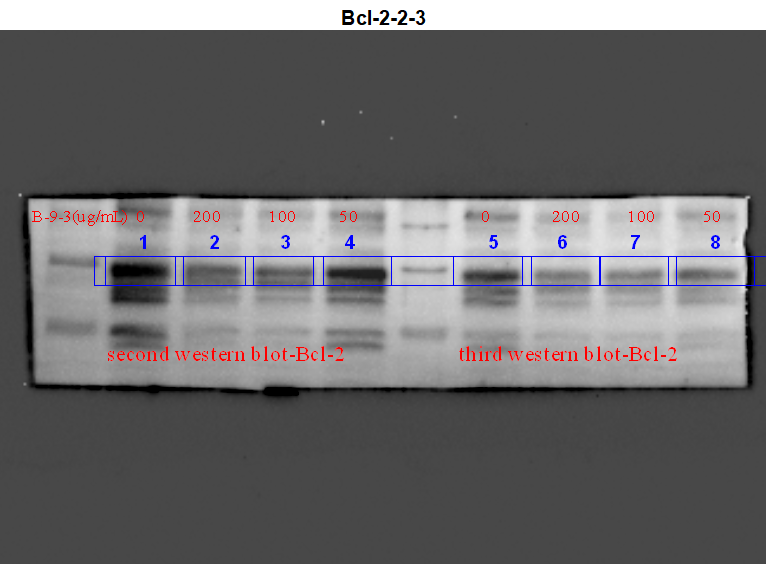


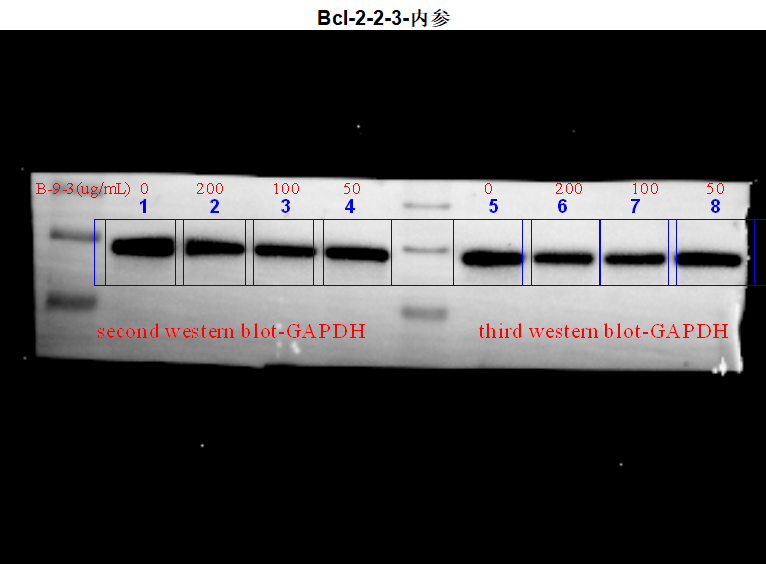


H460-Bcl-2


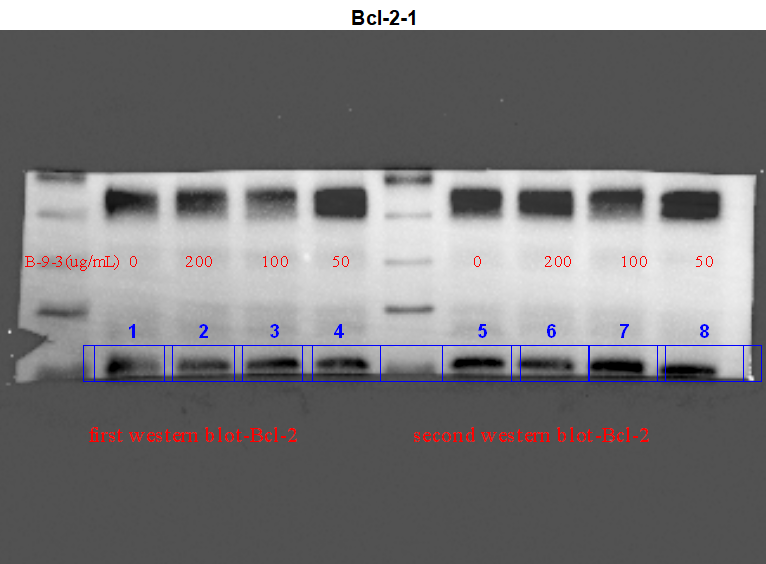

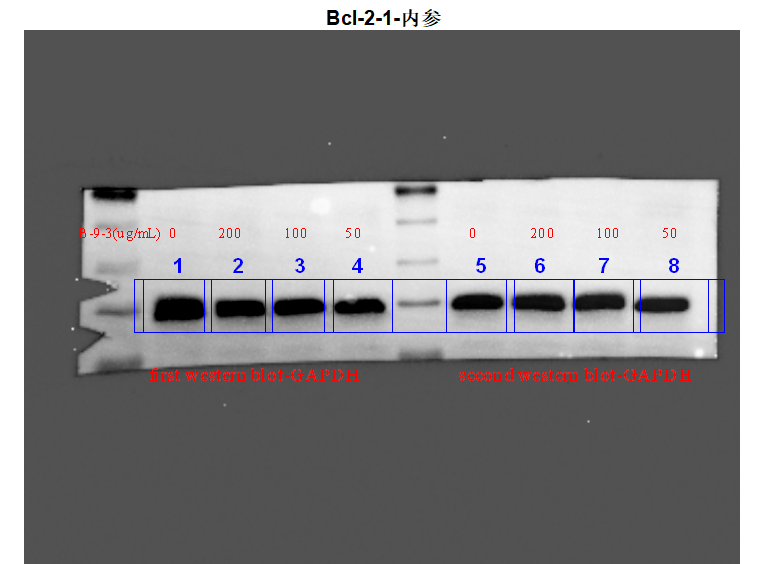


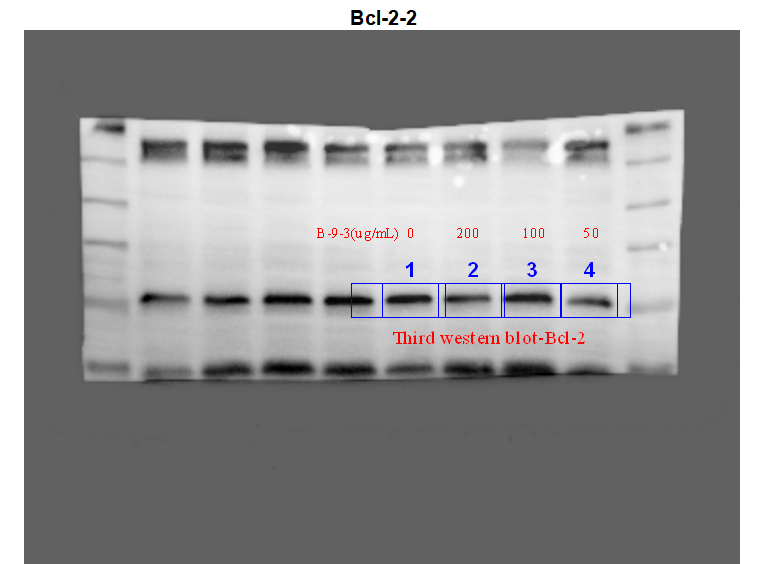

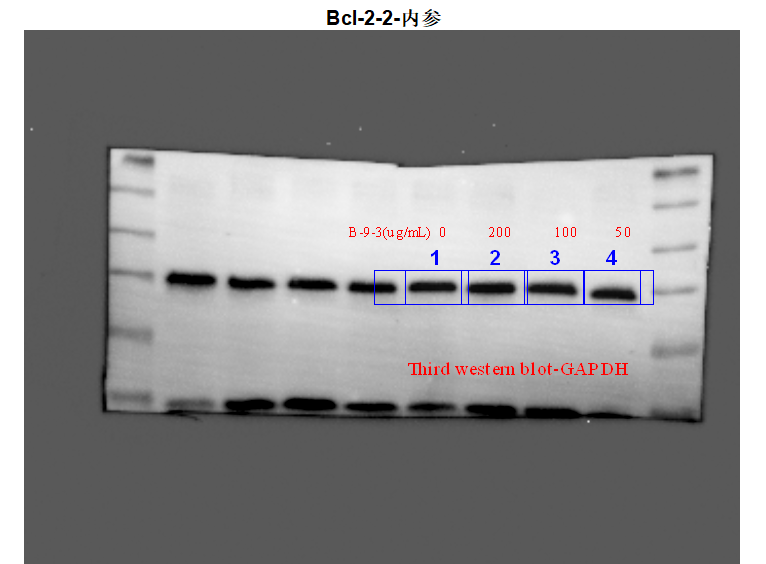


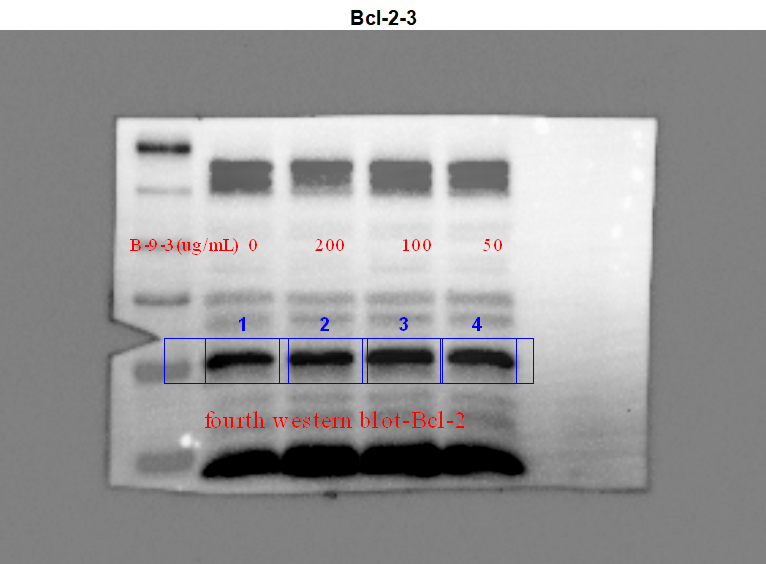

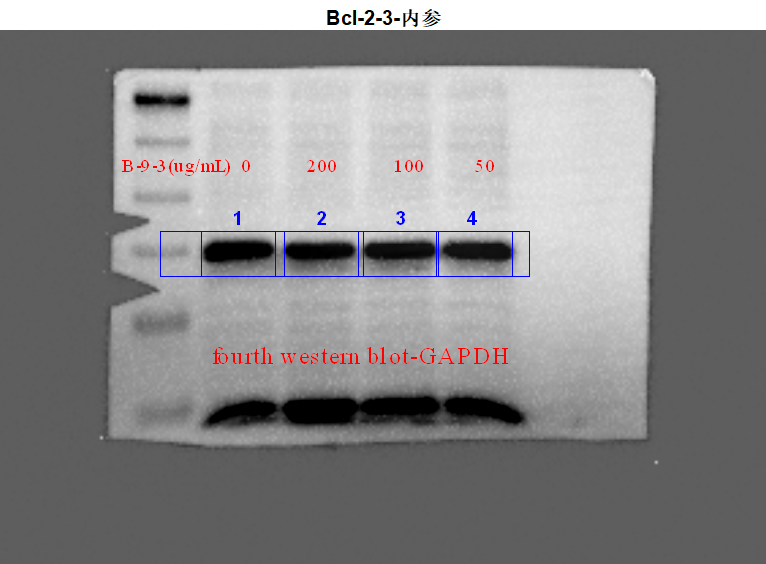


A549-Caspase-3


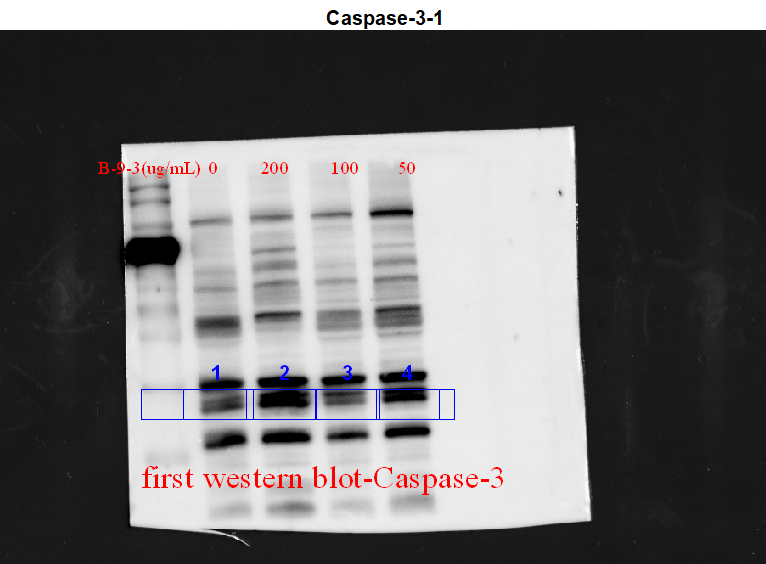

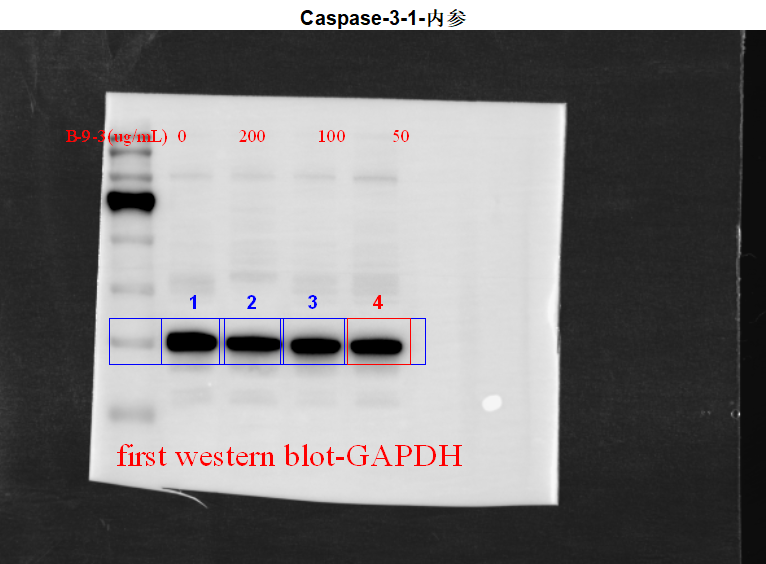


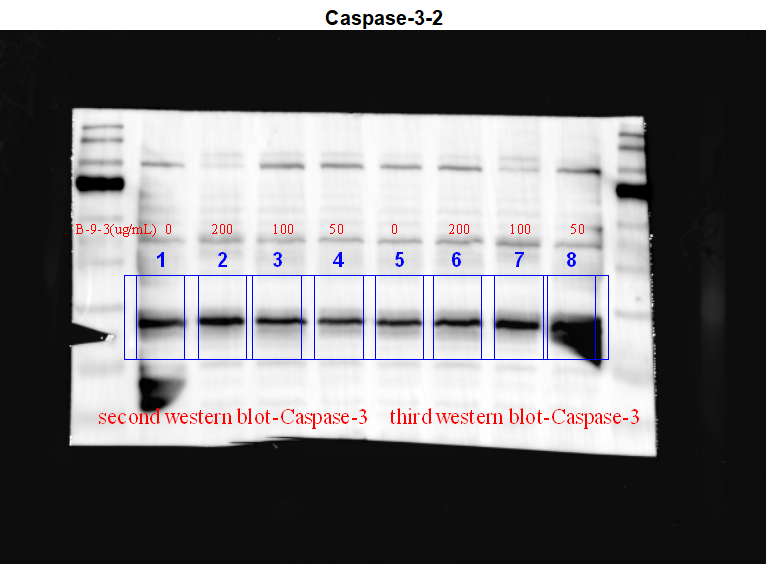

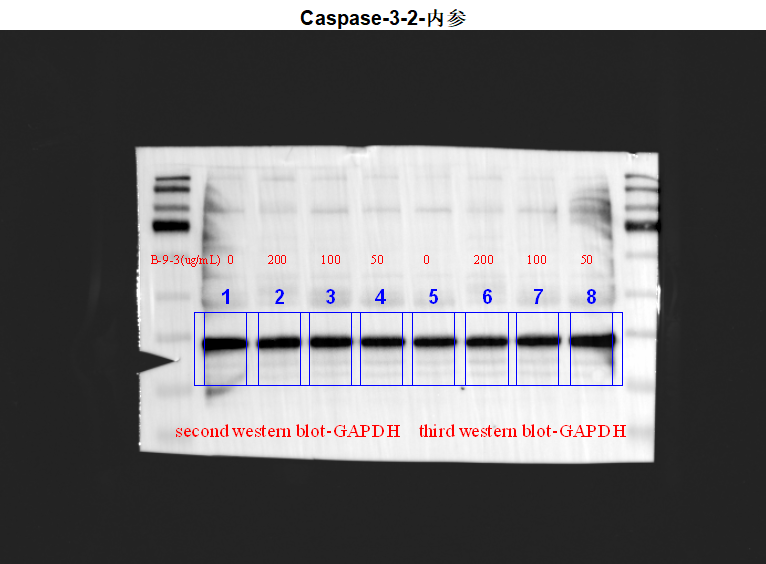


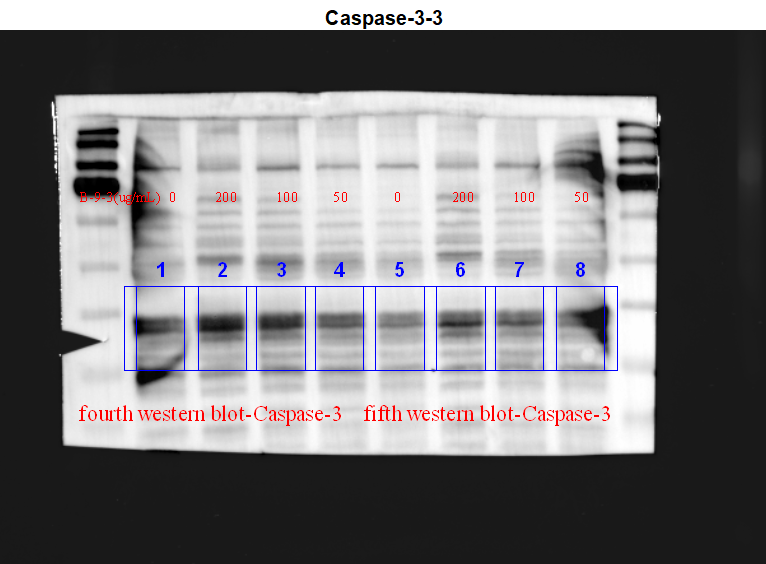

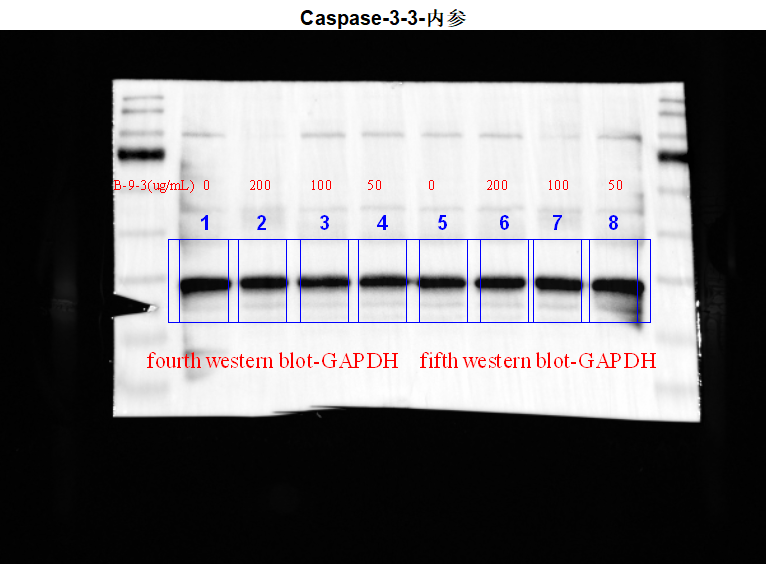


H226-Caspase-3

H460-Caspase-3
